# Processing of *Plasmodium falciparum* Merozoite Surface Protein MSP1 Activates a Spectrin-Binding Function Enabling Parasite Egress from RBCs

**DOI:** 10.1016/j.chom.2015.09.007

**Published:** 2015-10-14

**Authors:** Sujaan Das, Nadine Hertrich, Abigail J. Perrin, Chrislaine Withers-Martinez, Christine R. Collins, Matthew L. Jones, Jean M. Watermeyer, Elmar T. Fobes, Stephen R. Martin, Helen R. Saibil, Gavin J. Wright, Moritz Treeck, Christian Epp, Michael J. Blackman

**Affiliations:** 1 The Francis Crick Institute, Mill Hill Laboratory, Mill Hill, London, NW7 1AA, UK; 2 Department für Infektiologie, Parasitologie, Universitätsklinikum Heidelberg, D-69120 Heidelberg, Germany; 3 Wellcome Trust Sanger Institute, Hinxton, Cambridge, CB10 1HH, UK; 4 Department of Crystallography, Birkbeck College, London, WC1E 7HX, UK; 5 Department of Pathogen Molecular Biology, London School of Hygiene and Tropical Medicine, London, WC1E 7HT, UK

## Abstract

The malaria parasite *Plasmodium falciparum* replicates within erythrocytes, producing progeny merozoites that are released from infected cells via a poorly understood process called egress. The most abundant merozoite surface protein, MSP1, is synthesized as a large precursor that undergoes proteolytic maturation by the parasite protease SUB1 just prior to egress. The function of MSP1 and its processing are unknown. Here we show that SUB1-mediated processing of MSP1 is important for parasite viability. Processing modifies the secondary structure of MSP1 and activates its capacity to bind spectrin, a molecular scaffold protein that is the major component of the host erythrocyte cytoskeleton. Parasites expressing an inefficiently processed MSP1 mutant show delayed egress, and merozoites lacking surface-bound MSP1 display a severe egress defect. Our results indicate that interactions between SUB1-processed merozoite surface MSP1 and the spectrin network of the erythrocyte cytoskeleton facilitate host erythrocyte rupture to enable parasite egress.

## Highlights

• Merozoite surface protein MSP1 processing is important for *P. falciparum* viability
• Proteolytic processing activates MSP1’s heparin and spectrin-binding functions
• The rate of MSP1 processing governs the kinetics of parasite egress
• Loss of parasite surface MSP1 results in a severe egress defect

## Graphical Abstract

**Figure:**
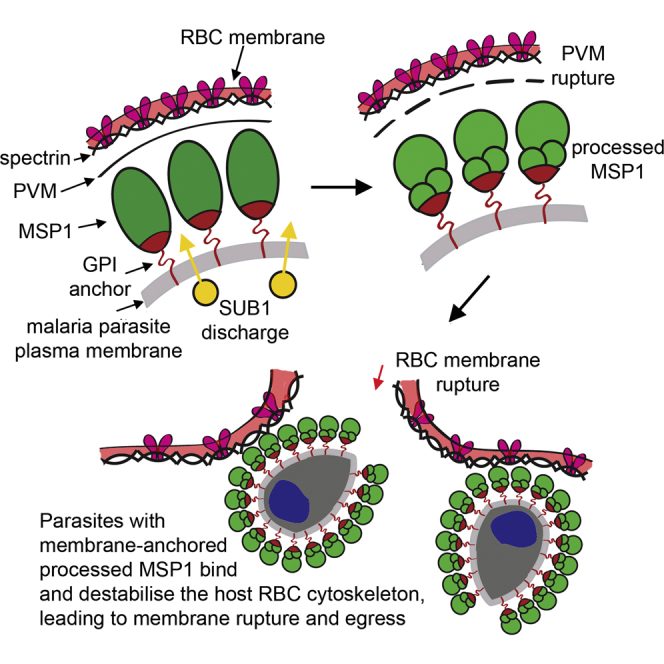

## Teaser

Egress from infected RBCs is a critical, but poorly understood, step in the malaria parasite’s lifecycle. Das et al. report that just prior to egress, proteolytic processing of parasite surface protein MSP1 activates a spectrin binding function, allowing the intracellular parasite to interact with the RBC cytoskeleton and enabling egress.

## Introduction

Malaria is a debilitating and often fatal infectious disease of tropical and subtropical regions. All associated pathology arises from intraerythrocytic replication of the protozoan parasite *Plasmodium*. For most of its erythrocytic life cycle, which lasts ∼48 hr in the most dangerous species, *P. falciparum*, the parasite resides within a parasitophorous vacuole (PV), sequestered from the host cell cytosol. Parasite growth leads to formation of a multinucleated schizont. Merozoites, polarized cells specialized for erythrocyte invasion, bud off from the mature schizont. Shortly thereafter, the PV membrane (PVM) ruptures, releasing the now freely mobile progeny merozoites into the residual erythrocyte cytosol. Within seconds, rupture of the host cell membrane allows egress of the merozoites to invade fresh erythrocytes (for a review of egress see Blackman and Carruthers, 2013).

At least 40 proteins localize to the merozoite surface (Cowman et al., 2012). Many of these traffic to the parasite plasma membrane during schizont development, where they are tethered via glycosyl phosphatidylinositol (GPI) anchors or through peripheral associations with GPI-anchored proteins. The most abundant merozoite surface component, a GPI-anchored protein called MSP1, is synthesized as an ∼200 kDa protein that in *P. falciparum* associates with at least two other peripheral proteins belonging to the MSP3 and MSP7 families (Kauth et al., 2006; Lin et al., 2014; Pachebat et al., 2001; Trucco et al., 2001). MSP1 is conserved throughout *Plasmodium* and has been scrutinized as a result of its capacity to induce antibody responses that inhibit parasite replication in vitro or protect in vivo (reviewed by Holder, 2009). Gene targeting experiments suggest that MSP1 is essential in the haploid blood stages (Combe et al., 2009; Drew et al., 2004; O’Donnell et al., 2000), but *msp1* null mutants could not be established so these studies provided little insight into MSP1 function. Bioinformatic analyses have been similarly uninformative, since MSP1 has no orthologs outside *Plasmodium* and structural information is sparse. The merozoite surface location of MSP1 has provoked speculation that it functions in erythrocyte invasion. Supporting this are reports that MSP1 binds to erythrocyte glycophorin A (Baldwin et al., 2015; Su et al., 1993), Band 3 (Goel et al., 2003; Li et al., 2004), and heparin-like molecules (Boyle et al., 2010; Zhang et al., 2013), while heparin and related polysaccharides block invasion by *P. falciparum* merozoites (Boyle et al., 2010; Clark et al., 1997; Crick et al., 2014; Kulane et al., 1992; Zhang et al., 2013). However, it remains to be demonstrated that MSP1 plays a primary role in invasion, and a mechanistic understanding of MSP1 function is lacking.

Minutes before egress, a serine protease called SUB1 is discharged from merozoite secretory organelles into the PV lumen, where it cleaves MSP1 and its partner proteins (Koussis et al., 2009; Silmon de Monerri et al., 2011; Yeoh et al., 2007). *P. falciparum* MSP1 is converted in this primary processing step into four fragments, which initially remain in a non-covalent complex on the merozoite surface (Holder et al., 1987; McBride and Heidrich, 1987). Following egress, MSP1 is further cleaved at a juxtamembrane site by a second parasite protease called SUB2 (Harris et al., 2005), shedding the bulk of the MSP1 complex (Blackman et al., 1991; Riglar et al., 2011). Spatiotemporal regulation of these processing steps is important for parasite viability (Child et al., 2010). Discharge of SUB1—and hence the timing of primary processing—is controlled by a parasite protein kinase (PKG), and inhibition of SUB1 discharge or activity prevents egress (Collins et al., 2013b; Taylor et al., 2010; Yeoh et al., 2007). Despite these insights, the role of MSP1 processing is unknown and a picture of how events following SUB1 discharge lead to rupture of the bounding membranes and erythrocyte cytoskeleton has yet to be established.

Here we show that processing by SUB1 enables MSP1 to interact with the host cell cytoskeleton to play a previously unsuspected role in egress.

## Results

### Alternative SUB1 Processing Sites in MSP1

**Figure 1:**
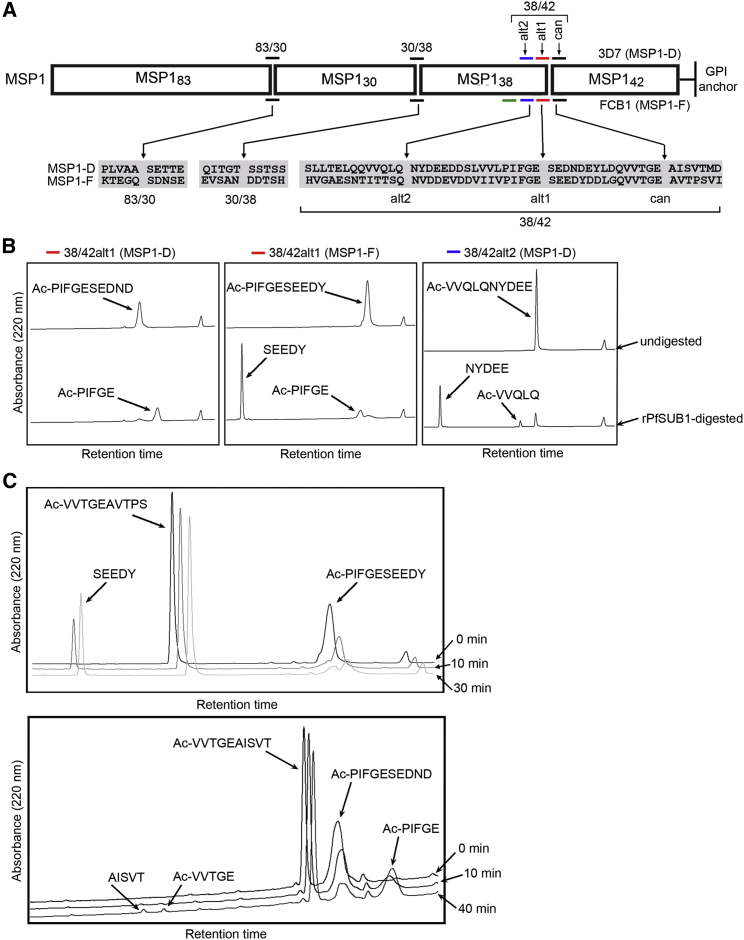
Alternative 38/42 Processing Sites in MSP1 (A) *P. falciparum* MSP1 and primary processing products. Known and predicted PfSUB1 cleavage sites in MSP1-D and MSP1-F (colored horizontal bars), above an alignment of flanking sequences, with experimentally confirmed cleavage sites arrowed and indicated by gaps. The 38/42 region contains the canonical cleavage site (can) as well as two additional sites (alt1 and alt2) confirmed in this work. MSP1-F contains a further predicted 38/42 site (HVGAE↓SNTIT; green bar) but this study found no evidence for cleavage at that site. (B) Cleavage by rPfSUB1 of peptides based on alternative 38/42 processing sites. RP-HPLC elution profiles of N-acetylated decapeptides before or after incubation with rPfSUB1. Parental peptide peaks diminished over time, with concomitant increase in the indicated products. In the case of Ac-PIFGESEDND the C-terminal cleavage product was too hydrophilic to bind to the RP-HPLC column. The small peak near the end of each chromatogram that does not alter with time represents elution of detergent from the digestion buffer. (C) The 38/42alt1 peptides are better substrates than the canonical 38/42 site peptides. Equimolar mixtures of peptides based on the canonical 38/42 and 38/42alt1 sites in MSP1-F and MSP1-D were incubated with rPfSUB1 and the peak area for substrate and product(s) monitored with time. Initial cleavage rates were compared after no more than 10% of the fastest cleaved peptide had been hydrolyzed, though for clarity extended digestions are also shown. Cleavage of both 38/42alt1 peptides occurred at least 6.7 times faster than cleavage of the corresponding canonical 38/42 site peptide. See also Figure S1.

*P. falciparum* MSP1 is a polymorphic protein that exists in two major isoforms, typified by those of the 3D7 and FCB1 parasite isolates. N-terminal sequencing has mapped three positionally conserved primary processing sites in each of these MSP1 isoforms (Blackman et al., 1991; Cooper and Bujard, 1992; Heidrich et al., 1989; Koussis et al., 2009; Stafford et al., 1994). The sites are referred to as 83/30, 30/38, and 38/42, after the approximate masses of the cleavage products (Figure 1A). While all are cleaved by *P. falciparum* SUB1 (PfSUB1), they are structurally distinct, consistent with evidence that PfSUB1 accommodates flexibility in its recognition motif (Withers-Martinez et al., 2012). Only the 38/42 site (i.e., that closest to the C terminus of MSP1) shows significant similarity between 3D7 MSP1 (MSP1-D) and FCB1 MSP1 (MSP1-F) (Figure 1A). Cleavage at the 38/42 site is a rate-limiting processing step (Child et al., 2010), implying special importance.

Since the identification of SUB1 as the enzyme responsible for MSP1 processing, the possibility of additional processing sites has not been explored. PfSUB1 substrate recognition is dominated by a preference for an aliphatic residue at the P4 position (numbering according to (Schechter and Berger, 1967), a small uncharged residue at P2, a polar residue at P1, and acidic residues at one or more of the P1′–P5′ positions (Withers-Martinez et al., 2012). In early work examining cleavage of recombinant MSP1-D in parasite extracts, Cooper and Bujard (1992) identified two additional cleavage sites adjacent to the canonical 38/42 site, suggesting redundancy. These motifs (VVQLQ↓NYDEE and PIFGE↓SEDND in MSP1-D), which are partially conserved in MSP1-F (Figure 1A), bear hallmarks of PfSUB1 sites, so we tested whether recombinant PfSUB1 (rPfSUB1) could cleave peptides based on them. All were cleaved at their central bond (Figure 1B), suggesting that both the alternative 38/42 sites in MSP1-D, and at least one of the alternative sites in MSP1-F, might be authentic processing sites. As both lie close to the canonical 38/42 site, they are referred to as the 38/42alt1 and 38/42alt2 sites (Figure 1A). In kinetic assays, the 38/42alt1 peptides from both MSP1 isoforms were cleaved ∼7-fold faster than the respective canonical 38/42 peptides (Figure 1C), an observation important for subsequent work.

### Mutation of MSP1 Prevents PfSUB1-Mediated Processing In Vitro

To begin to address the importance of MSP1 processing, mutations were introduced into a recombinant product called Fwt heterodimer (Kauth et al., 2003), which comprises the two “halves” of MSP1-F refolded into a stoichiometric complex. Substitution of the P2 and P2′ positions at the 83/30 site (mutant Fmut83/30; Figure S1A) ablated cleavage by rPfSUB1 at this position (Figure S1B). Similarly, a recombinant full-length MSP1-F called Fwt (Kauth et al., 2006) with P4 and P2 substitutions at the 30/38 site (mutant Fmut30/38; Figure S1A) was refractory to cleavage at this site (Figure S1C). This showed that appropriate mutations prevent processing and indicated an absence of alternative sites at the 83/30 and 30/38 positions in MSP1-F.

To examine the potential for preventing cleavage at the 38/42 sites, we produced further Fwt heterodimer mutants designed to block cleavage at one or more of these sites, and at a third putative alternative 38/42 site unique to MSP1-F (Figure 1A). Cleavage within the 38/42 region was abolished by simultaneous mutation of the canonical, alt1, alt2, and putative third alternative sites, but mutagenesis of only one site, or two sites together, or the canonical and alt1 sites plus the putative third alternative site, was insufficient to block cleavage (Figure S1D).

In view of the special importance of the 38/42 site, this analysis was extended using a full-length recombinant MSP1-D called rMSP1-DCD4wt. Simultaneous mutagenesis of the canonical, alt1, and alt2 sites completely blocked cleavage within the 38/42 region (Figure S1E). Together, these results confirmed the presence of alternative 38/42 sites in both MSP1 isoforms and identified mutations that prevent all PfSUB1-mediated cleavage.

### PfSUB1 Processing of an MSP1 Transgene Product Is Important for Parasite Viability

**Figure 2:**
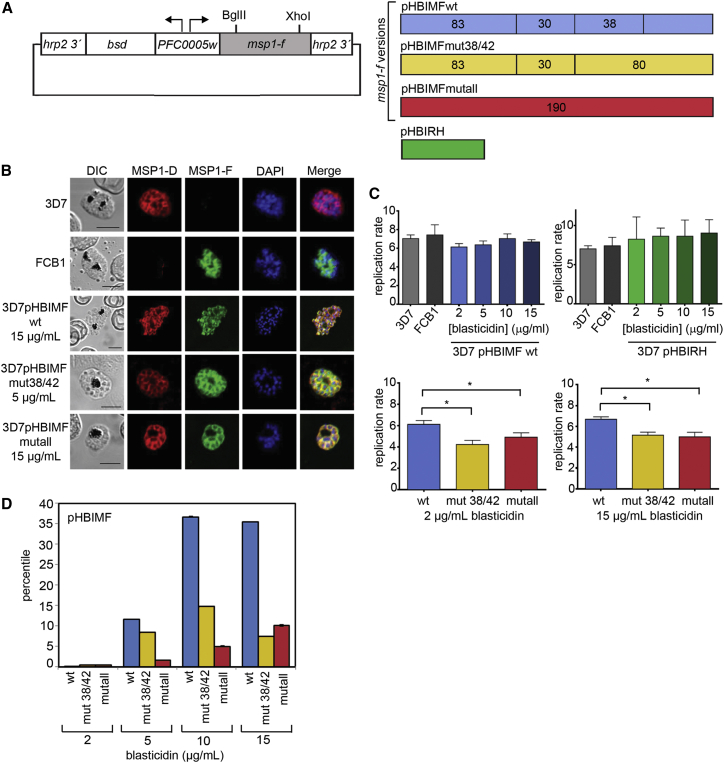
Episomal Expression of Cleavage-Resistant MSP1 Inhibits *P. falciparum* Growth (A) Blasticidin-regulated co-selection episome. A bi-directional *P. falciparum* promoter (the intron of PlasmoDB: PFC0005w) drives expression of the blasticidin-S-deaminase gene (*bsd*) and *msp1-f* transgene. The *hrp2* gene 3′ UTR controls transgene transcript termination and polyadenylation. Three variants were used, expressing wild-type *msp-1f* (pHBIMFwt, blue), or with mutations at all four known and putative 38/42 sites, (pHIBMFmut38/42, yellow; same mutations as Fmut38/42triple, Figure S1A) or mutations at all primary processing sites (pHBIMFmutall, red; same as mutant Fmutall, Figure S1A). All *msp1-f* sequences included the GPI anchor sequence. Increasing blasticidin concentration selects for parasites harboring multi-copy concatamers to maintain drug resistance, leading to increased *msp1-f* expression. A construct containing the *Renilla* luciferase gene (pHBIRH, green) was used as control. (B) Immunofluorescence analysis (IFA) of parental 3D7 and FCB1 schizonts, as well as 3D7 schizonts harboring the constructs in the indicated concentrations of blasticidin. Parasites were probed with MSP1 isoform-specific antibodies. Merged signals include that of the DNA dye 4,6-diamidino-2-phenylindole (DAPI, blue). Scale bar, 5 μm. (C) Quantification by FACS of parasite replication over a single erythrocytic cycle. Top: no significant differences between parental parasites and the transgenic 3D7pHBIMFwt and 3D7pHBIRH lines. Bottom: replication of the 3D7pHBIMFwt line compared to the 3D7pHIBMFmut38/42 and 3D7pHBIMFmutall lines expressing mutant MSP1-F, at similar blasticidin concentrations. Columns show mean values of >3 biological replicates. Error bars, SEM. Statistically different growth rates are indicated (^∗^p < 0.05; ^∗∗^p < 0.01. Kruskal-Wallis test). (D) Transgene RNA transcript levels measured by qRT-PCR, as a percentile of endogenous *msp1-d* transcript levels (100%). SEM values in all cases were <0.1%. See also Figure S2.

To test whether mutations that prevent processing are tolerated by *P. falciparum*, we adopted two complementary strategies. First, we exploited an episomal transgene expression system (Epp et al., 2008) that allows blasticidin-regulated control of expression levels. Constructs for expression of three forms of MSP1-F (Figure 2A; Figures S2A, and S2B) were transfected into 3D7 *P. falciparum*, then antibodies specific for MSP1-F used to examine transgene expression on the background of endogenous MSP1-D. Parasites harboring a wild-type *msp1-f* transgene (3D7pHBIMFwt), or the same gene with mutations at all putative 38/42 sites (3D7pHBIMFmut38/42), or at all primary processing sites (3D7pHBIMFmutall), correctly expressed the transgene product on developing merozoites at all blasticidin concentrations tested (Figure 2B; Figure S2C). Varying blasticidin levels from 2–15 μg ml^−1^ did not affect growth of the 3D7pHBIMFwt line or parasites harboring a control plasmid, pHBIRH (Figure 2C, top). However, parasites harboring mutant constructs pHBIMFmut38/42 and pHBIMFmutall showed significantly lower growth rates than the 3D7pHBIMFwt line (Figure 2C, bottom). Whereas the 3D7pHBIMFwt line responded to increases in blasticidin concentration by substantially upregulating *msp1-f* transcript levels (Figure 2D; Figure S2D), likely via increases in episome copy number (confirmed by copy number estimation, data not shown), much less upregulation was seen in the mutant lines, indicating an inability to respond to elevated drug concentrations. Since the episomes differed only at the *msp1-f* cleavage sites, these results suggested that expression of cleavage-resistant MSP1, even in the presence of endogenous MSP1, is deleterious.

### Processing of MSP1 in the 38/42 Region Is Important for Parasite Viability

**Figure 3:**
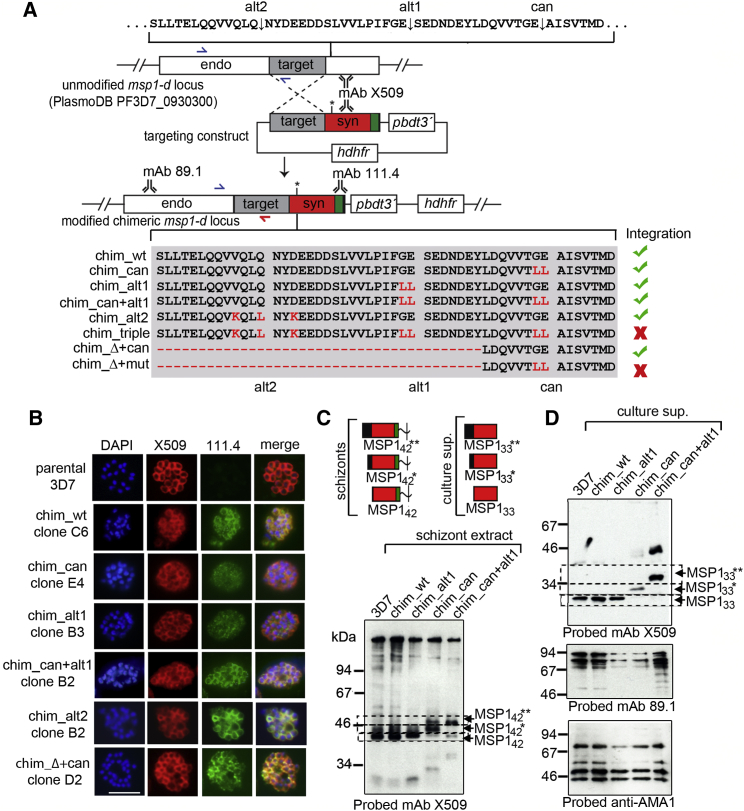
Mutations that Prevent All Processing in the 38/42 Region Are Deleterious (A) Top: modification of the *P. falciparum msp1-d* locus by single-crossover homologous recombination. The targeting region (gray) incorporated into the integration constructs was fused just upstream of the 38/42 region to recodonized sequence (red/green, syn) encoding the rest of the ORF. In all except pHH1MSP1chim_wt, this incorporated mutations and/or deletions to block processing at one or more 38/42 cleavage sites (asterisk). The 3′ end of the recodonized sequence (green) contained the MSP1-F-specific mAb 111.4 epitope. Positions of hybridization of primers used for diagnostic PCR are indicated (blue and red half arrows). Integration replaced the *msp1* 3′ UTR with that of the *P. berghei* dihydrofolate reductase gene (*pbdt*). The *hdhfr* cassette confers resistance to WR99210. Integration produces a modified locus encoding a chimeric MSP1 recognized by both mAb X509 and mAb 111.4. Bottom: substitutions or deletions (hyphens) introduced by the constructs are indicated (red). Green tick, successful integration. Red cross, no integration detected. (B) IFA of schizonts of the parental and transgenic *P. falciparum* clones. All transgenics reacted with mAb 111.4. Scale bar, 5 μm. (C) Top: schematic of the MSP1_42_ fragment (GPI anchored) and the slightly larger MSP1_42_^∗^ and MSP1_42_^∗∗^ species predicted to result from ablation of cleavage at the canonical and canonical plus 38/42alt1 sites, respectively. The MSP1_33_ and modified MSP1_33_^∗^ and MSP1_33_^∗∗^ fragments, derived from cleavage by SUB2 within MSP1_42_, MSP1_42_^∗^, and MSP1_42_^∗∗^, respectively, are also depicted. Bottom: western blot shows differences (highlighted, dotted lines) in migration of the wild-type and modified MSP1_42_ forms. (D) Western blot of culture supernatants shows differences in migration of MSP1_33_, MSP1_33_^∗^, and MSP1_33_^∗∗^ (highlighted as above). As controls, supernatants were probed with mAb 89.1, which recognizes MSP1_83_, or antibodies to an irrelevant shed parasite protein, AMA1. See also Figure S3.

In a second approach to evaluating the importance of PfSUB1-mediated MSP1 processing, we sought to modify the endogenous *msp1* locus using homologous recombination to introduce mutations that prevent processing within the 38/42 region (the importance of processing at the 83/30 and 30/38 sites was not further examined). Our approach used a previously described strategy (Child et al., 2010) in which we transfected 3D7 parasites with constructs containing targeting sequence fused to synthetic “recodonized” sequence encoding a chimeric MSP1 C-terminal domain (Figure 3A). Integration produces a chimeric gene, the product of which can be distinguished from unmodified MSP1-D by its reactivity with the MSP1-F-specific monoclonal antibody (mAb) 111.4. Integration thus epitope tags the gene.

Four integration constructs were initially generated (Figure 3A). Construct pHH1MSP1chim_wt was designed to replace the 3′ region of the *msp1* ORF with the chimeric sequence but leave the 38/42 processing sites unaltered. It thus acted as a control for all other genetic experiments. Constructs pHH1MSP1chim_can and pHH1MSP1chim_alt1 were identical to pHH1MSP1chim_wt except that they were designed to introduce di-leucine mutations at the P2 and P1 positions of the canonical 38/42 site or the 38/42alt1 site, respectively; these substitutions blocked processing of recombinant MSP1 (Figure S1). Construct pHH1MSP1chim_can+alt1 was designed to introduce both these sets of substitutions upon integration, thus blocking cleavage at both the canonical and 38/42alt1 sites. Parasites independently transfected with the constructs were subjected to drug cycling (growth in the absence then presence of WR99210) to select for integration. PCR analysis detected integration of all constructs by drug cycle 2 (data not shown), and clones derived from the drug-resistant lines were recognized by mAb 111.4, confirming correct integration (Figure 3B, top five rows). Sequencing of PCR products amplified from the modified *msp1* locus of the clones confirmed the presence of the mutations (data not shown).

The transgenic clones grew normally, indicating no effects of the mutations on viability (data not shown). To assess the impact on MSP1 processing, schizont extracts and culture supernatants containing shed MSP1 fragments were examined by western blot. This showed a shift in migration of the MSP1_42_ cleavage product in the chim_can and chim_can+alt1 clones (Figure 3C), with production of progressively larger fragments termed MSP1_42_^∗^ and MSP1_42_^∗∗^. This was consistent with blockade of cleavage at the canonical site (chim_can) or at both modified sites (chim_can+alt1), resulting instead in cleavage at the 38/42alt1 site or the 38/42alt2 site, respectively. Examination of culture supernatants (Figure 3D) showed increases in the mass of the shed MSP1_33_ fragment in the chim_can and chim_can+alt1 clones, again consistent with ablation of processing at the canonical 38/42 site, or both sites, respectively. To confirm the site of cleavage when processing at both the canonical and 38/42alt1 sites was prevented, we purified the MSP1_33_^∗∗^ species from culture medium of a chim_can+alt1 clone (Figure S3). Edman degradation identified its N terminus as NYDEE, confirming the 38/42alt2 cleavage site and proving the presence of alternative, redundant 38/42 cleavage sites in MSP1-D. The lack of a growth defect in the chim_can, chim_alt1, and chim_can+alt1 parasite clones proved that cleavage at the canonical or 38/42alt1 sites is not essential; blocking cleavage at one or both positions simply shifted cleavage to an alternative available 38/42 site.

To examine the effects of blocking processing at all three 38/42 positions, four additional transfection constructs were generated (Figure 3A). Constructs pHH1MSP1chim_alt2 and pHH1MSP1chim_triple were designed to introduce mutations that block cleavage at the 38/42alt2 site or all three 38/42 sites respectively (Figure S1). In addition, pHH1MSP1chim_Δ+can and pHH1MSP1chim_Δ+mut were designed to delete a 69-residue predicted unstructured (data not shown) segment of MSP1 sequence that encompasses both the 38/42alt1 and 38/42alt2 sites. Construct pHH1MSP1chim_Δ+can was designed to leave the canonical 38/42 site unaltered, whereas pHH1MSP1chim_Δ+mut would additionally render this site non-cleavable. Both pHH1MSP1chim_alt2 and pHH1MSP1chim_Δ+can rapidly integrated (data not shown), and the resulting parasite clones showed the expected reactivity with mAb 111.4 (Figure 3B, bottom 2 rows). In contrast, despite five independent transfection experiments, each with extended periods of drug cycling, integration of pHH1MSP1chim_triple and pHH1MSP1chim_Δ+mut was never detected. Since, aside from the mutations unique to these constructs, they were identical to the other six constructs that readily integrated, this result suggested that cleavage of at least one position within the 38/42 region of MSP1 is important for parasite viability.

### Processing of MSP1 Alters Its Secondary Structure and Activates Spectrin and Heparin-Binding Activity

**Figure 4:**
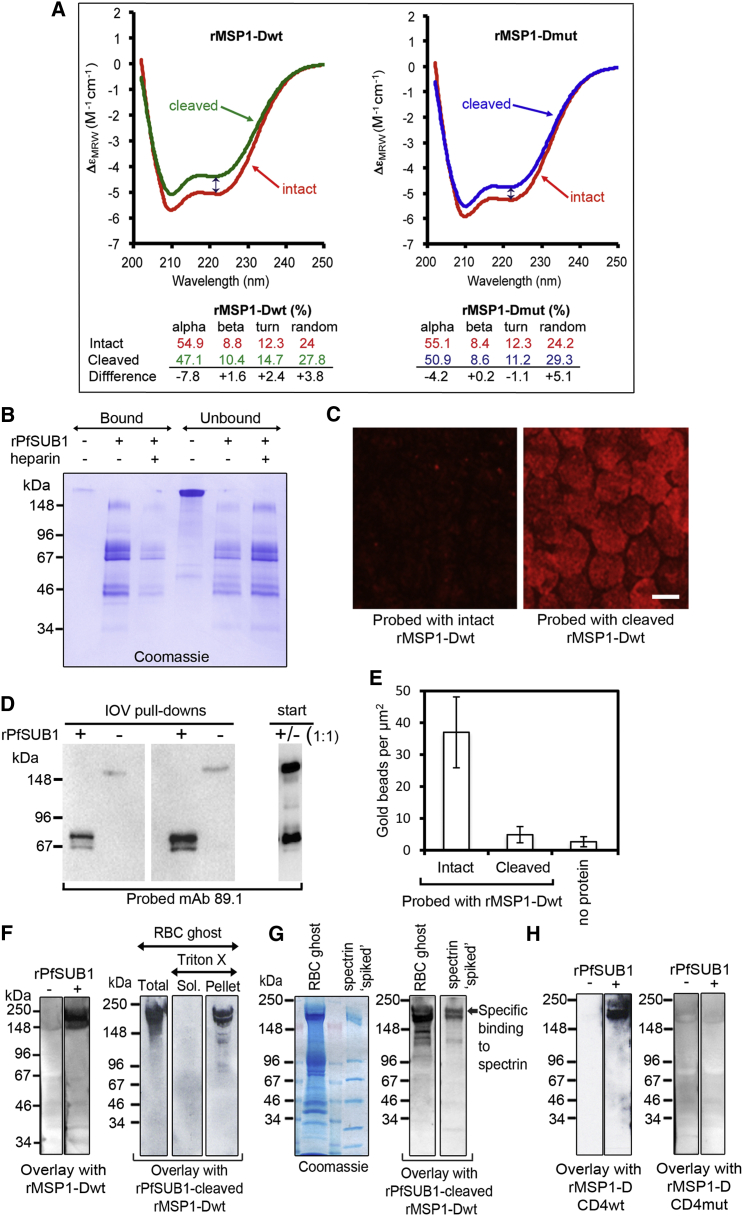
Processing Alters MSP1 Secondary Structure and Activates a Heparin and Spectrin-Binding Activity (A) Far-UV CD spectra of rMSP1-Dwt and rMSP1-Dmut as a function of molar absorptivity at 37°C. The vertical double arrow at 222 nm (negative minimum for the alpha helix spectrum) highlights the reduction in CD intensity following cleavage, which was 1.4-fold greater for rMSP1-Dwt than for rMSP1-Dmut. Below: secondary structure composition of the intact and processed proteins. (B) Processing enhances heparin binding. Intact or cleaved rMSP1-Dwt was incubated with heparin agarose ± soluble heparin (1 mg ml^−1^), then binding assessed by SDS PAGE. Quantification of band intensity (Image Lab) showed that 72.3% ± 6.5% of cleaved rMSP1-Dwt bound heparin agarose but only 18.3% ± 12.1% of intact rMSP1-Dwt (p = 0.006, Student’s t test). (C) Fixed, permeabilized erythrocytes probed with intact or rPfSUB1-cleaved rMSP1-Dwt. Binding was detected by IFA and imaged using equal exposure times. Mean pixel intensity (Adobe Photoshop Histogram tool) was 41.6 ± 4.7 (cleaved) and 12.6 ± 1.4 (uncleaved). Scale bar, 5 μm. (D) Processing enhances binding to IOVs. Vesicles (∼80 μg protein) incubated with intact or rPfSUB1-cleaved rMSP1-Dwt (4 μg) were washed then two different loadings analyzed by western blot in parallel with a 1:1 mixture of the starting protein preparations. (E) Processing enhances binding to the erythrocyte cytoskeleton. Mean density of bound gold beads following immunoEM of Triton X-100-treated erythrocyte ghosts incubated with intact or rPfSUB1-cleaved rMSP1-Dwt then probed with anti-MSP1 antibodies and 5 nm gold-conjugated secondary antibodies. Error bars, SD. (F) Overlay assay. Erythrocyte ghosts or Triton X-100-fractionated ghosts were separated by SDS PAGE, transferred to nitrocellulose, then probed with rPfSUB1-cleaved or intact rMSP1-Dwt and binding detected with anti-MSP1 antibodies. (G) Overlay assay. Erythrocyte ghosts, or purified erythrocyte spectrin (Sigma) mixed with molecular mass marker proteins (GE Healthcare), were subjected to SDS PAGE and either stained or transferred to nitrocellulose and probed as in (F) with rPfSUB1-cleaved rMSP1-Dwt. (H) Overlay assay. Erythrocyte ghosts probed as in (F) with intact or rPfSUB1-cleaved rMSP1-DCD4wt or rMSP1-DCD4mut. The latter, which is refractory to cleavage in the 38/42 region, did not bind spectrin. See also Figure S4.

Size-exclusion chromatography of rPfSUB1-cleaved rMSP1-DCD4wt as well as a similar protein lacking the CD4 tag (rMSP1-Dwt; Figure S1E) showed that, like parasite MSP1 (McBride and Heidrich, 1987), the processed products remain associated under non-denaturing conditions (Figure S4A). This encouraged us to use the recombinant proteins to examine the structural consequences of cleavage. Circular dichroism (CD) of intact and rPfSUB1-processed rMSP1-Dwt, as well as of a mutant (rMSP1-Dmut) that was refractory to cleavage in the 38/42 region (Figure S1E), showed that processing altered the secondary structure of both proteins (Figure 4A). These changes were less extensive in rMSP1-Dmut, indicating that cleavage at the 38/42 region contributed to the conformational rearrangements.

MSP1 has been implicated in interactions with erythrocyte surface heparin-like polysaccharides, so we compared the capacity of intact and rPfSUB1-cleaved rMSP1-Dwt to bind to immobilized heparin. Cleaved rMSP1-Dwt showed ∼4-fold higher binding than intact protein, which was reduced by soluble heparin (Figure 4B). We next examined the ability of rMSP1-Dwt to bind to intact erythrocytes. No binding was detected (data not shown). However, permeabilized erythrocytes incubated with cleaved rMSP1-Dwt showed an ∼3.3-fold more intense IFA signal than cells exposed to intact rMSP1-Dwt (Figure 4C), suggesting that cleavage enhanced binding to an intraerythrocytic component. This was confirmed in pull-down assays using inside-out erythrocyte ghost vesicles (IOVs) (Figure 4D), as well as by immunoEM analysis of erythrocyte cytoskeletons (Figure 4E; Figure S4D), which in both cases showed preferential binding of cleaved rMSP1-Dwt. To determine the target(s) of binding, we probed SDS PAGE-fractionated erythrocyte ghosts in overlay assays with intact or rPfSUB1-processed rMSP1-Dwt. Cleaved rMSP1-Dwt bound exclusively to a Triton X-100-insoluble doublet migrating at the positions of α- and β-spectrin, the dominant components of the cytoskeleton (Figure 4F). This was confirmed by probing purified spectrin “spiked” with irrelevant proteins (Figure 4G). No binding was observed for intact or rPfSUB1-treated rMSP1-DCD4mut (Figure 4H), showing that cleavage within the 38/42 region—already shown to be important for parasite viability—was required for binding to spectrin.

### PfSUB1-Mediated Processing of MSP1 Plays a Role in Egress

**Figure 5:**
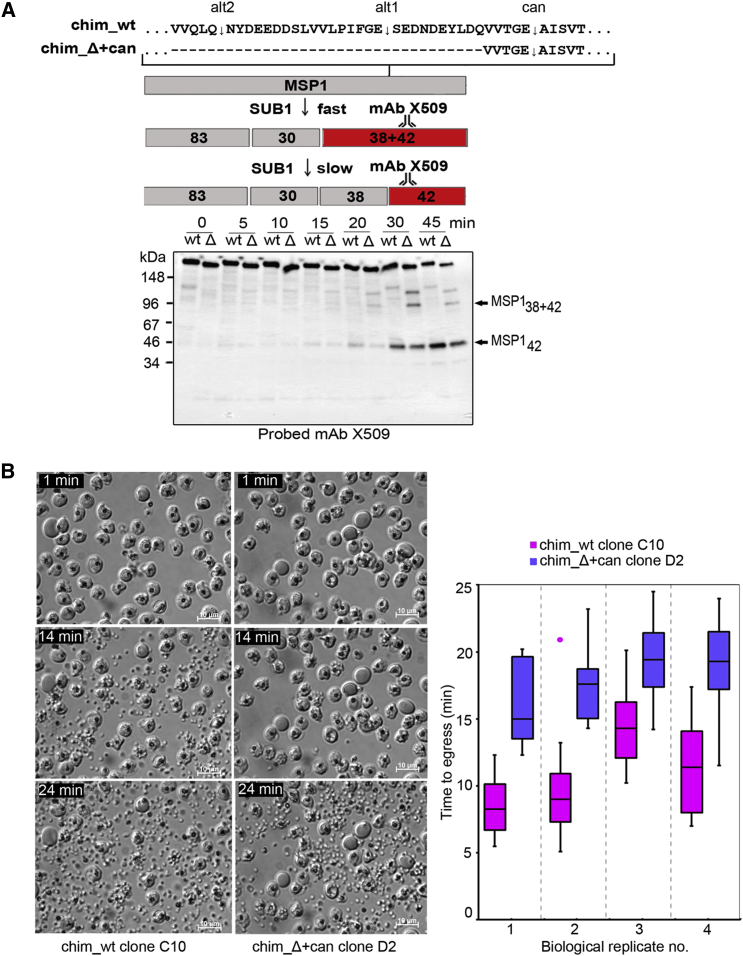
Rate of MSP1 Processing Regulates the Kinetics of Egress (A) Top: the 38/42 region in chim_Δ+can and chim_wt parasites and the MSP1 processing pathway (Child et al., 2010). Bottom: time course comparing processing of MSP1 from chim_Δ+can and chim_wt clones by western blot. Schizonts were sampled at the indicated times following removal of a C1 block. Note the slightly smaller full-length chim_Δ+can MSP1 due to the 69-residue deletion. (B) Left: stills from time-lapse microscopy of chim_Δ+can and chim_wt clones (imaging started 4 min 20 s after C1 removal). Right: box plot comparison of time to egress after C1 removal. Data are from 4 independent experiments each assessing 12–24 egress events per clone. Whiskers, range. A single outlier point (>1.5× the interquartile range) indicated. The chim_Δ+can clones showed a mean egress delay of 7.5 ± 1.4 min (p < 0.005, Student’s t test). Similar results were obtained with two other chim_Δ+can and chim_wt clones (data not shown). See also Figure S5 and Movies S1 and S2.

The erythrocyte cytoskeleton lies beneath the cell membrane so merozoites are unlikely to contact it during invasion. However, intracellular merozoites impinge on the inner face of the host cell membrane in the brief period between PVM rupture and egress (e.g., Glushakova et al., 2009), so we explored the possibility that direct interactions between processed merozoite surface MSP1 and host cell spectrin might play a part in egress. For this, we returned to the chim_Δ+can mutant (Figure 3) in which an MSP1 segment had been deleted to remove the 38/42alt1 and 38/42alt2 sites entirely, leaving just the canonical 38/42 cleavage site. Since this is a relatively poor substrate for PfSUB1 (Figure 1C), we predicted that cleavage within the 38/42 region in the chim_Δ+can mutant should be less efficient than in wild-type parasites. To test this, we compared the kinetics of processing in chim_Δ+can parasites with that in chim_wt parasites, which expressed the same chimeric MSP1 but retained all three 38/42 cleavage sites. For these experiments, schizonts were treated with the reversible PKG inhibitor compound 1 (C1), which prevents PfSUB1 discharge, stalling schizont development at the final stage of maturation. MSP1 processing and egress occur within minutes of washing away the inhibitor (Collins et al., 2013b). As shown in Figure 5A, processing of MSP1 in the chim_Δ+can mutant was delayed relative to chim_wt parasites and was characterized by an unusually prominent MSP1_38+42_ processing intermediate. Comparison of the kinetics of chim_Δ+can and chim_wt egress by time-lapse microscopy showed a reproducible delay in egress in the chim_Δ+can parasites following C1 removal (Figure 5B; Movie S1), mirroring the delay in MSP1 processing. This was confirmed in further experiments in which the clones were imaged simultaneously following fluorescent labeling of one population to identify it (Movie S2; Figure S5). Since the chim_Δ+can and chim_wt parasites differed only by the presence or absence of an MSP1 segment encompassing the 38/42alt1 and alt2 cleavage sites, these results showed that processing of MSP1 regulates the kinetics of egress.

### Truncation of MSP1 to Remove Its Merozoite Surface Anchor Produces an Egress Defect

**Figure 6:**
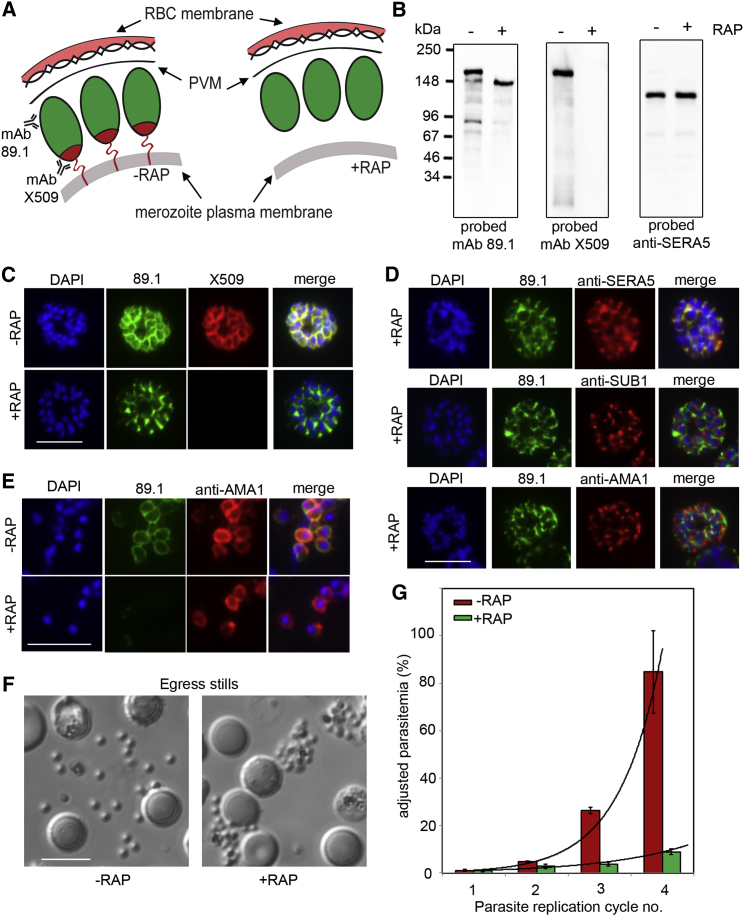
Truncation of MSP1 Produces an Egress Defect (A) Predicted RAP-induced MSP1 truncation in the 3D7MSP1flox42C clones, showing loss of the GPI anchor and C-terminal domain containing the mAb X509 epitope. (B) MSP1 truncation confirmed by western blot of 3D7MSP1flox42C1 clone E3 schizonts, 44 hr following treatment ± RAP. The PV protein SERA5 was used as a loading control. (C) RAP treatment produces a loss of mAb X509 reactivity and a shift in the IFA pattern of MSP1 to one typical of PV proteins, consistent with the predicted truncation. Numbers of DAPI-stained nuclei did not differ between control and RAP-treated schizonts (mean values: 21.2 ± 3.4 and 20.6 ± 4.0 nuclei per schizont, respectively, n = 24). (D) IFA showing co-localization of truncated MSP1 with SERA5 indicating a PV location. The punctate localization of SUB1 and the microneme protein AMA1 indicates normal organelle biogenesis. (E) IFA showing lack of surface-bound MSP1 on merozoites of RAP-treated 3D7MSP1flox42C1 clone E3. Antibodies to AMA1 (which is expressed on free merozoites) were used as a control. (F) Stills from time-lapse DIC microscopic imaging of egress in control and RAP-treated 3D7MSP1flox42C1 clone E3. Scale bar, 10 μm. (G) Replication rates of RAP- or control-treated 3D7MSP1flox42C1 clone E3. Cultures were passaged at intervals by 10-fold dilution into fresh medium plus erythrocytes as described in Supplemental Experimental Procedures. Observed parasitaemia values were adjusted for these dilutions and are displayed as adjusted values. The plot shows mean values of three biological replicate experiments. Error bars, SEM. The RAP-treated cultures showed an ∼2.1-fold reduction in replication rate per cycle, but this was an over-estimate of mutant viability due to rapid expansion of the few (∼1%) non-excised parasites in the RAP-treated cultures. See also Figure S6 and Movies S3 and S4.

MSP1 is tethered to the merozoite surface via a C-terminal GPI anchor. To further test our model that direct interactions between merozoite-bound MSP1 and the erythrocyte cytoskeleton facilitates egress, we used a recently published conditional strategy to generate *P. falciparum* transgenics in which a 3′ segment of the *msp1* gene could be deleted by rapamycin (RAP)-inducible, Cre recombinase-mediated excision (Figure 6A; Figure S6A) (Collins et al., 2013a). This was predicted to generate a truncated MSP1 that lacked a GPI anchor and so would not be bound to the merozoite surface. Analysis of RAP-treated 3D7MSP1flox42C parasites showed highly efficient excision, resulting in exclusive expression of truncated MSP1 in mature schizonts at the end of the same erythrocytic cycle (Figures 6B and 6C). No effects on merozoite development were discernible. The modified MSP1 was trafficked to the PV as expected for a non-membrane-bound merozoite surface protein (Figures 6C–6D; Figure S6B) but was not present on the surface of free merozoites (Figure 6E). Video microscopy of the RAP-treated 3D7MSP1flox42C schizonts revealed a dramatic egress defect characterized by abortive erythrocyte membrane rupture and trapping of the merozoites in the partially ruptured cell (Figure 6F; Movies S3 and S4). Consistent with this, the RAP-treated mutants displayed a substantially reduced replication rate (Figure 6G and Figure S6C). These results show that MSP1 functions at egress and that this role requires it to be tethered to the merozoite surface. Collectively, our findings support the model that processing of MSP1 facilitates host cell membrane rupture, probably through interactions between the mature merozoite surface and the erythrocyte cytoskeleton.

## Discussion

**Figure 7:**
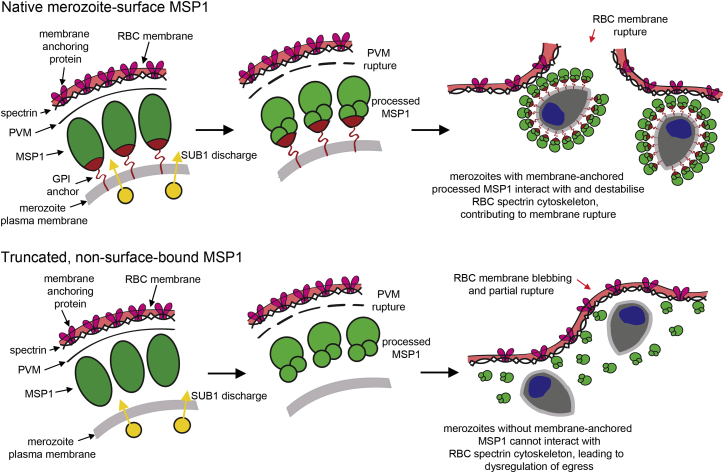
Model for the Role of MSP1 Processing in Egress See also Movie S5.

We have combined genetic, structural, and functional analysis with microscopic observation of egress to produce evidence that: (1) proteolytic maturation of MSP1 by SUB1 is important for parasite viability; (2) proteolysis alters MSP1 secondary structure, conferring upon it a capacity to bind to both heparin and erythrocyte spectrin; and (3) these functional alterations regulate egress, probably as a result of interactions between MSP1 and the host cell cytoskeleton. The resistance of the erythrocyte membrane to mechanical shear stress is dependent on the structural integrity of its cytoskeleton and in particular its underlying lattice of spectrin tetramers formed by the head-to-head association of pairs of αβ spectrin heterodimers. The spectrin network is dynamic, accommodating reversible breakage and reformation of the dimer-dimer bonds in response to even moderate shear stress (e.g., Salomao et al., 2006). Shear forces can also result in unfolding of the triple-helical repeat units that comprise α- and β-spectrin, providing additional flexibility (Randles et al., 2007). This dynamic state allows peptides and other small molecules that interfere with tetramer stability (Salomao et al., 2006) or that perturb interactions between spectrin and other cytoskeletal components such as ankyrin (Blanc et al., 2010), protein 4.1R, and actin (An et al., 2007) to destabilize the membrane. SUB1-processed MSP1 may perform an analogous role. We speculate that following PVM breakdown, the diffusive movement of intracellular merozoites impinging upon the inner face of the erythrocyte membrane—well documented by both time-lapse and diffraction phase microscopy (Chandramohanadas et al., 2011; Gilson and Crabb, 2009; Glushakova et al., 2010; Glushakova et al., 2009) (see also Movie S5)—enables merozoite surface-bound MSP1 to bind the spectrin lattice, producing internal shear forces that disrupt the cytoskeleton (Figure 7). This is likely aided by protease activity, perhaps involving host cell calpain-1 (Chandramohanadas et al., 2009) and/or the PfSUB1 substrate SERA6 (Ruecker et al., 2012), since the cysteine protease inhibitor E64 selectively inhibits host cell membrane rupture (e.g., Glushakova et al., 2009). Even localized destabilization of the cytoskeleton may be sufficient to allow egress, since high-speed video microscopy has shown that erythrocyte membrane rupture initiates at a single site; subsequent elastic inversion of the membrane promotes its rapid disintegration (Abkarian et al., 2011; Crick et al., 2013). Interestingly, Herrera et al. (1993) reported spectrin-binding activity for a recombinant MSP1 polypeptide, suggested by those authors as being important for intracellular parasite development. We do not favor that model, since parasites replicate within the PVM, which shields them from the host cytoskeleton. In contrast, the egress delay observed in the chim_Δ+can mutant, and the egress defect (with no effect on schizont development) when MSP1 is conditionally converted to a non merozoite-bound form, implies a role for processed merozoite-bound MSP1 in host cell rupture. Our model explains the defect associated with episomal expression of cleavage-resistant MSP1 (Figure 2), which presumably reduces egress efficiency by reducing the proportion of MSP1 at the merozoite surface able to interact with the host cell cytoskeleton. These data implicate a surface protein in the egress of an intracellular non-viral pathogen. They also provide a plausible mechanistic rationale for the timing of MSP1 processing by SUB1, which “prepares” the merozoites for partaking in their own release.

We do not rule out additional roles for MSP1. A previous report (Combe et al., 2009) showed that knockdown of MSP1 expression in parasite liver stages ablated merozoite formation, suggesting a role in merozoite budding. Additionally, our observation that processing enhances binding to heparin tempts speculation that SUB1 may activate MSP1 to perform a function at invasion. However, the fact that RAP-treated 3D7MSP1flox42C parasites lacking surface-bound MSP1 produce normal numbers of merozoites and replicate in vitro (albeit at a very reduced rate) shows that merozoite surface MSP1 is dispensable for merozoite development and invasion in blood stages. Compounds that inhibit MSP1 processing or that block interactions with spectrin may form the basis of antimalarial drugs that interfere with this key step in the malarial life cycle.

## Experimental Procedures

### Parasite Culture, Transfection, and Growth Assays

*P. falciparum* clones FCB1, 3D7, and 1G5DC (Collins et al., 2013a) were maintained in RPMI 1640 medium with Albumax (Invitrogen) and synchronized using standard procedures (Blackman, 1994). Transfection, selection with WR99210 (Jacobus Pharmaceuticals), and cloning was as described (Collins et al., 2013a; Harris et al., 2005). Growth rates were determined by microscopy or fluorescence-activated cell sorting (FACS) as described (Stallmach et al., 2015). Details of transfection constructs based on the pHBIRH episome (Epp et al., 2008) and integration plasmid pMSP1chimWT (Child et al., 2010) are provided in Supplemental Experimental Procedures, as are details of the construct used to flank a segment of the 1G5DC *msp1* ORF with *loxP* sites. For conditional truncation of MSP1 in the 3D7MSP1flox42C clones, synchronous ring-stage parasites were treated for 4 hr with 100 nM RAP (Collins et al., 2013a).

### Recombinant Proteins and Antibodies

Monoclonal antibodies 89.1, X509, and 111.4, rabbit polyclonal antibodies and their use in western blot and IFA analysis have been described (Blackman et al., 1991; Child et al., 2010; Ruecker et al., 2012). Production and purification of rPfSUB1, Fwt, and Fwt heterodimer was as described (Kauth et al., 2003, 2006; Withers-Martinez et al., 2012). Mutants of Fwt and Fwt heterodimer were produced using QuikChange II (Agilent) site-directed mutagenesis of parent plasmids. For rMSP1-Dwt, MSP1-D (minus its GPI anchor) was expressed in HEK293E cells (Crosnier et al., 2013); for rMSP1-DCD4wt, it was fused to domains 3 and 4 of rat CD4. Cleavage site mutants were produced by replacing segments of the expression constructs with synthetic gene fragments containing substitutions. The proteins were purified by nickel chelate and size-exclusion chromatography.

### Peptide Cleavage Assays and N-Terminal Sequencing

Synthetic peptides were from Biomatik (http://www.biomatik.com). Peptide cleavage assays and product identification by RP-HPLC and mass spectrometry were as described (Koussis et al., 2009; Withers-Martinez et al., 2012). To purify shed MSP1 fragments, 3D7 or chim_can+alt1 schizonts were allowed to undergo egress in protein-free medium then the supernatants fractionated on a Vydac 4.6 × 150 mm 214TP C4 RP-HPLC column. The MSP1_33_ and MSP1_33_^∗∗^ species were identified by western blot then the proteins transferred to PVDF membrane for N-terminal sequencing (PNAC).

### Quantitative Real-Time PCR

First strand cDNA synthesis was performed using a SuperScript II First-Strand Synthesis Kit (Invitrogen) according to the manufacturer’s instructions. Quantitative real-time PCR (qRT-PCR) was performed using the ABI 7500 sequence detection system and a SensiFASTSYBR Lo-ROX kit (Bioline). Data were analyzed with SDS 1.3.1 software (Applied Biosystems). Transgene expression was displayed as a percentile of endogenous *msp1-d* expression (100%).

### Circular Dichroism and Secondary Structure Predictions

Purified rMSP1-Dwt and rMSP1-Dmut (0.156 mg ml^−1^ in 500 μl 25 mM HEPES [pH 7.4], 150 mM NaCl, 15 mM CaCl_2_) were monitored on a Jasco J-715 spectropolarimeter for 5 hr at 37°C with or without added rPfSUB1 (6 μl at 0.84 mg ml^−1^). Secondary structure composition was averaged using CONTINLL, SELCON3, and CDSSTR (Sreerama and Woody, 2004). Secondary structure predictions were performed with JPred (http://www.compbio.dundee.ac.uk/www-jpred/).

### Heparin-Binding, Overlay Assays, IOV Pulldown Assays and immunoEM

Heparin-agarose beads (Sigma) in assay buffer (25 mM HEPES [pH 7.4], 15 mM NaCl, 0.07% Tween 20) were incubated with intact or cleaved rMSP1-Dwt (50 μl at 0.1 μg μl^−1^). Control samples were additionally supplemented with heparin sodium salt (1 mg ml^−1^, Sigma). Following incubation for 20 min at room temperature, supernatants containing unbound proteins were recovered and the beads washed five times with assay buffer. Bound proteins were eluted into 50 μl SDS sample buffer then all samples subjected to reducing SDS-PAGE on a 4%–16% gradient gel. The gel was stained with Coomassie blue, imaged using a BioRad Chemidoc MP system and band intensities estimated using Image Lab software.

Overlay assays to detect binding to SDS-PAGE fractionated human erythrocyte ghost proteins were as described by Herrera et al. (1993). IOVs were prepared using standard procedures from erythrocyte ghosts (see Supplemental Experimental Procedures) and incubated in PBS with intact or rPfSUB1-cleaved rMSP1-Dwt, rMSP1-DCD4wt, or rMSP1-DCD4mut before washing and analysis by western blot, detecting bound proteins with mAb 89.1. For immunoEM analysis, TX-100-treated cytoskeletons immobilized on grid grids were incubated with intact or cleaved rMSP1-Dwt (0.1 μg ml^−1^) then washed and probed with anti-MSP1 antibodies followed by 5 nm gold-conjugated anti-rabbit IgG, before staining with sodium silicotungstate.

### Time-Lapse Microscopy

*P. falciparum* egress was imaged as described (Collins et al., 2013b), using C1 to synchronize egress. Microscopic DIC images were routinely collected at 5 s intervals for up to 30 min. For comparison of 3D7 chim_Δ+can and 3D7 chim_wt parasites, populations were either alternately imaged or combined in the same microscopy chamber after labeling one mutant with Hoechst 33342 prior to washing away C1. An initial fluorescence image was collected prior to starting the time-lapse DIC imaging, then the fluorescence and first DIC images overlayed to identify labeled cells. Image files were exported as AVI movies using Axiovision 3.1 software. Time to individual egress events was recorded by visual examination of movie frames.

## Author Contributions

S.D., N.H., and E.T.F. performed the experiments. A.J.P. produced recombinant MSP1. C.W.-M. and S.R.M. performed biophysical analyses. M.L.J. and C.R.C. developed conditional methodologies. H.R.S., G.J.W., M.T., C.E., and M.J.B. supervised the work. S.D., N.H., C.E., and M.J.B. wrote the manuscript.

## Supplemental Information

Document S1. Supplemental Experimental Procedures, Figures S1–S6, and Tables S1 and S2

Movie S1, Related to Figure 5. chim_Δ+can Parasites Display an Egress Delay Compared to chim_wt Parasites Synchronous schizonts of chim_wt clone C10 (left) and chim_Δ+can clone D2 (right) were Percoll enriched and then returned to culture and allowed to mature for 4–5 hr in the presence of the PKG inhibitor compound 1 (C1, 2 μM). The parasites were washed in fresh warm medium without C1 and observed by time-lapse DIC microscopy, taking images at 5 s intervals. Imaging commenced precisely 4 min 20 s following C1 removal. Time after start of microscopy is indicated (top left). The mean delay in time to egress for chim_Δ+can clone D2 schizonts relative to chim_wt clone C10 in this experiment was 5.2 min (p < 0.001, Student’s t test) (biological replicate no. 3 in Figure 5). Similar results were obtained for separate distinct clones (derived from a separate transfection) of each transgenic parasite line (chim_wt clone C6 and chim_Δ+can clone E6; not shown).

Movie S2, Related to Figure 5. chim_Δ+can Parasites Display an Egress Delay Compared to chim_wt Parasites Synchronous schizonts of chim_wt clone C6 and chim_Δ+can clone E6 were Percoll enriched and then returned to culture and allowed to mature for 4–5 hr in the presence of C1. Just before use, the chim_Δ+can clone E6 parasites were treated for 5 min with Hoechst 33342 (1 μg ml^−1^). The labeled parasites were then washed in warm medium containing C1 to remove excess dye prior to mixing at a 1:1 ratio with the chim_wt parasites. The schizont mixture was washed in fresh warm medium without C1 and observed by time-lapse DIC microscopy (left), taking images at 5 s intervals. Imaging commenced exactly 5 min following C1 removal. A single fluorescence image together with a DIC image (right) was recorded just prior to starting the time-lapse imaging, enabling the chim_wt (red circles) and chim_Δ+can (blue circles) schizonts to be identified by overlaying the images. Time after start of microscopy is indicated (top left). The mean delay in time to egress for the chim_Δ+can clone E6 schizonts in this video relative to chim_wt clone C10 was 5.7 min (p < 0.002) (Figure S5). A similar delay was observed in chim_Δ+can parasites in reciprocal experiments in which the chim_wt schizonts were instead labeled with Hoechst 33342 (not shown), showing that the delay in egress was not caused by the labeling.

Movie S3, Related to Figure 6. Dysregulation of Egress in Parasites Expressing Truncated, Non-Merozoite-Bound MSP1 Synchronized, newly invaded forms of 3D7MSP1flox42C1 clone E3 were treated with RAP (100 nM) or vehicle only (DMSO, 1% v/v) for 4 hr, then washed and cultured for a further ∼44 hr to allow schizont development. The schizonts were Percoll-enriched, returned to culture and allowed to mature for 4–5 hr in the presence of C1, then washed in warm medium without C1 and immediately observed by time-lapse DIC microscopy, taking images at 5 s intervals. Imaging commenced precisely 4 min 20 s following C1 removal. Control-treated parasites (left) underwent normal “explosive” egress with rapid dissemination of daughter merozoites. In contrast, RAP-treated parasites (right) appeared to undergo normal PVM rupture (e.g., arrowed schizont) but abortive RBC membrane rupture, with inefficient release of merozoites. Identical results were observed for 3DMSP1flox42C2 clone B51 (not shown).

Movie S4, Related to Figure 6. Dysregulation of Egress in Parasites Expressing Truncated, Non-Merozoite-Bound MSP1 Synchronized, newly invaded forms of 3D7MSP1flox42C1 clone E3 were treated with RAP (100 nM) or vehicle only (DMSO, 1% v/v) for 4 hr, then washed and cultured for a further ∼44 hr to allow schizont development. The schizonts were Percoll-enriched, returned to culture and allowed to mature for 4–5 hr in the presence of C1, then washed in warm medium without C1 and immediately observed by time-lapse DIC microscopy, taking images at 5 s intervals. Imaging commenced precisely 4 min 20 s following C1 removal. Control-treated parasites (left) underwent normal “explosive” egress with rapid dissemination of daughter merozoites. In contrast, RAP-treated parasites (right) appeared to undergo normal PVM rupture but abortive RBC membrane rupture, with inefficient release of merozoites. Identical results were observed for 3DMSP1flox42C2 clone B51 (not shown).

Movie S5, Related to Figure 7. P. *falciparum* Egress May Involve Shear Forces Induced by Movement of Intracellular Merozoites Time-lapse DIC video microscopy of wild-type 3D7 *P. falciparum* showing a single egress event selected because it clearly shows that egress is a two-step process. Rupture of the PVM at ∼14.1 min is followed by transient intracellular movement of the merozoites. This allows repeated impingement of the free merozoites upon the inner face of the host erythrocyte membrane before final membrane rupture and egress. Images were taken at 5 s intervals and the movie frame rate is 10 frames/s.

Document S2. Article plus Supplemental Information
