# Supplementary figures and images for: Processing of Plasmodium falciparum Merozoite Surface Protein MSP1 Activates a Spectrin-Binding Function Enabling Parasite Egress from RBCs
Source: Cell Host Microbe. 2015 Oct 14;18(4):433–44. doi: 10.1016/j.chom.2015.09.007 (PMC4608996; doi:10.1016/j.chom.2015.09.007)

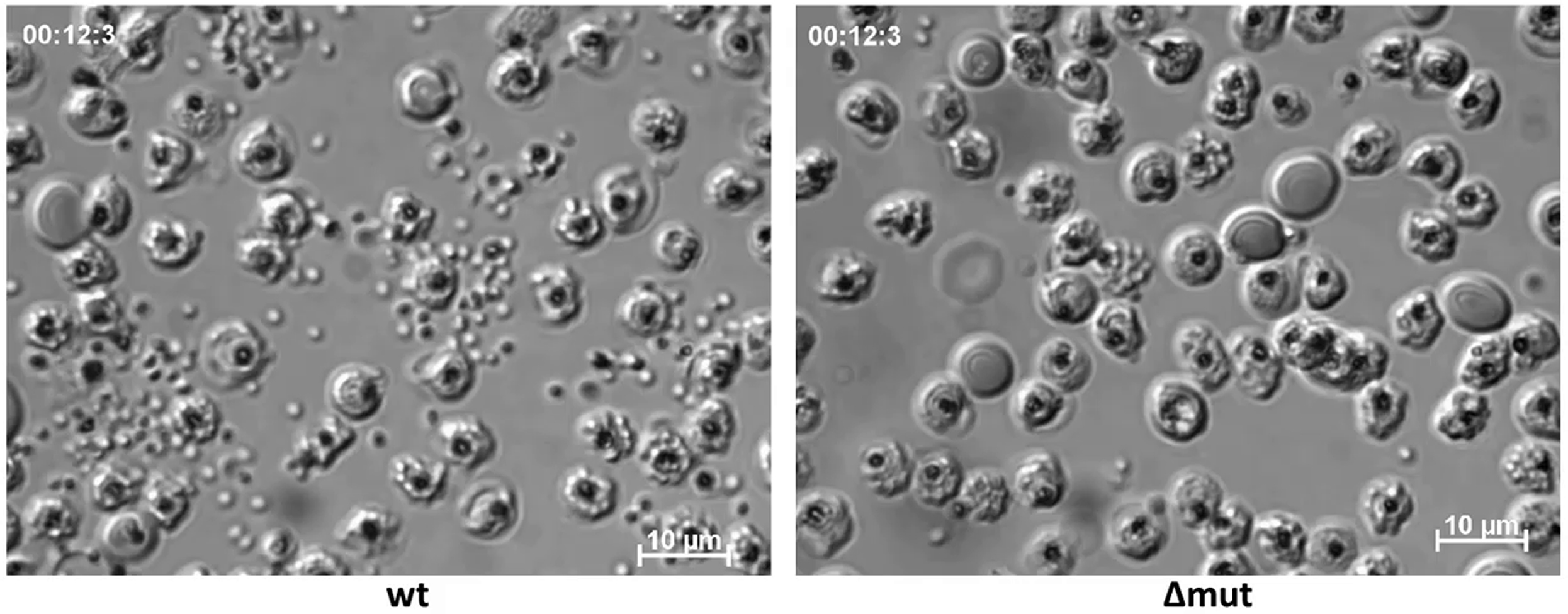

Supplement: Movie S1, Related to Figure 5. chim_Δ+can Parasites Display an Egress Delay Compared to chim_wt Parasites — Synchronous schizonts of chim_wt clone C10 (left) and chim_Δ+can clone D2 (right) were Percoll enriched and then returned to culture and allowed to mature for 4–5 hr in the presence of the PKG inhibitor compound 1 (C1, 2 μM). The parasites were washed in fresh warm medium without C1 and observed by time-lapse DIC microscopy, taking images at 5 s intervals. Imaging commenced precisely 4 min 20 s following C1 removal. Time after start of microscopy is indicated (top left). The mean delay in time to egress for chim_Δ+can clone D2 schizonts relative to chim_wt clone C10 in this experiment was 5.2 min (p < 0.001, Student’s t test) (biological replicate no. 3 in Figure 5). Similar results were obtained for separate distinct clones (derived from a separate transfection) of each transgenic parasite line (chim_wt clone C6 and chim_Δ+can clone E6; not shown). [file mmc2.jpg]

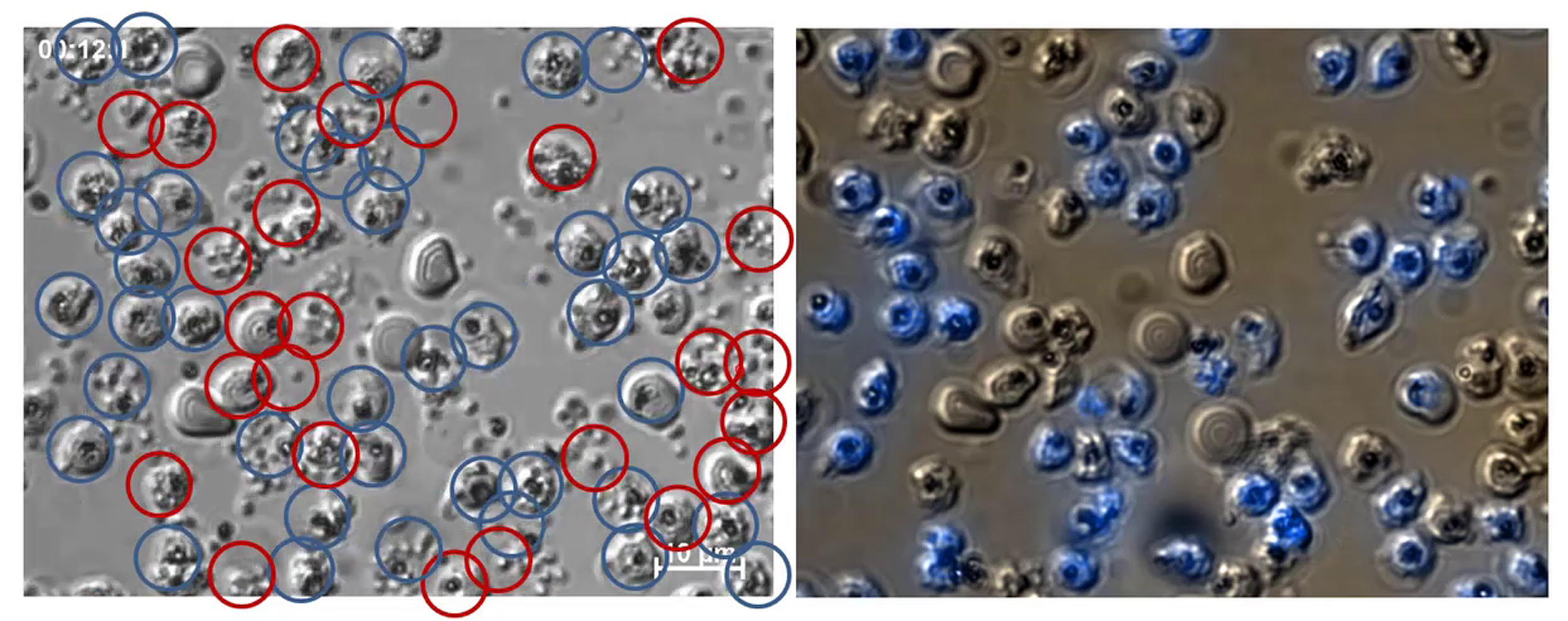

Supplement: Movie S2, Related to Figure 5. chim_Δ+can Parasites Display an Egress Delay Compared to chim_wt Parasites — Synchronous schizonts of chim_wt clone C6 and chim_Δ+can clone E6 were Percoll enriched and then returned to culture and allowed to mature for 4–5 hr in the presence of C1. Just before use, the chim_Δ+can clone E6 parasites were treated for 5 min with Hoechst 33342 (1 μg ml−1). The labeled parasites were then washed in warm medium containing C1 to remove excess dye prior to mixing at a 1:1 ratio with the chim_wt parasites. The schizont mixture was washed in fresh warm medium without C1 and observed by time-lapse DIC microscopy (left), taking images at 5 s intervals. Imaging commenced exactly 5 min following C1 removal. A single fluorescence image together with a DIC image (right) was recorded just prior to starting the time-lapse imaging, enabling the chim_wt (red circles) and chim_Δ+can (blue circles) schizonts to be identified by overlaying the images. Time after start of microscopy is indicated (top left). The mean delay in time to egress for the chim_Δ+can clone E6 schizonts in this video relative to chim_wt clone C10 was 5.7 min (p < 0.002) (Figure S5). A similar delay was observed in chim_Δ+can parasites in reciprocal experiments in which the chim_wt schizonts were instead labeled with Hoechst 33342 (not shown), showing that the delay in egress was not caused by the labeling. [file mmc3.jpg]

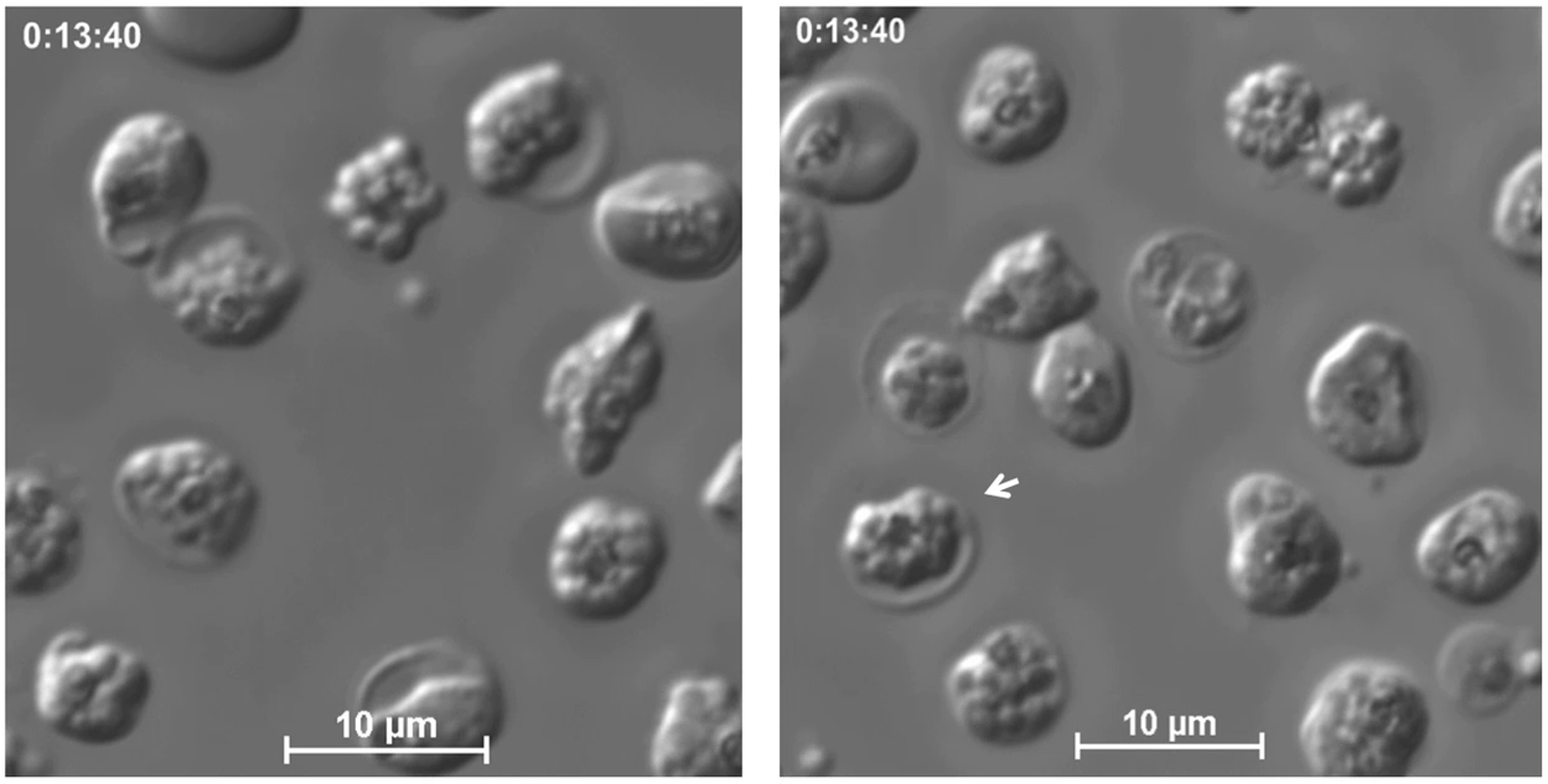

Supplement: Movie S3, Related to Figure 6. Dysregulation of Egress in Parasites Expressing Truncated, Non-Merozoite-Bound MSP1 — Synchronized, newly invaded forms of 3D7MSP1flox42C1 clone E3 were treated with RAP (100 nM) or vehicle only (DMSO, 1% v/v) for 4 hr, then washed and cultured for a further ∼44 hr to allow schizont development. The schizonts were Percoll-enriched, returned to culture and allowed to mature for 4–5 hr in the presence of C1, then washed in warm medium without C1 and immediately observed by time-lapse DIC microscopy, taking images at 5 s intervals. Imaging commenced precisely 4 min 20 s following C1 removal. Control-treated parasites (left) underwent normal “explosive” egress with rapid dissemination of daughter merozoites. In contrast, RAP-treated parasites (right) appeared to undergo normal PVM rupture (e.g., arrowed schizont) but abortive RBC membrane rupture, with inefficient release of merozoites. Identical results were observed for 3DMSP1flox42C2 clone B51 (not shown). [file mmc4.jpg]

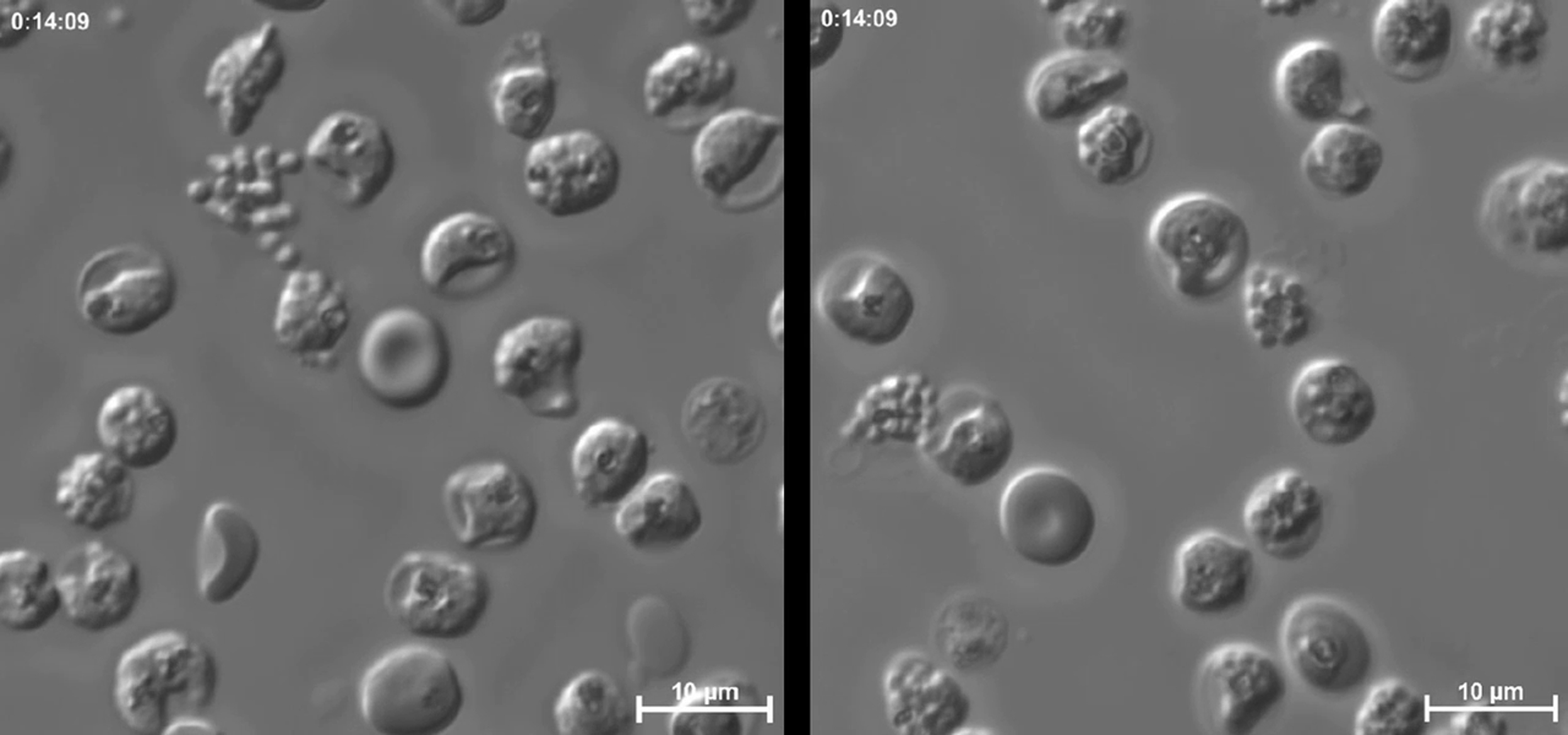

Supplement: Movie S4, Related to Figure 6. Dysregulation of Egress in Parasites Expressing Truncated, Non-Merozoite-Bound MSP1 — Synchronized, newly invaded forms of 3D7MSP1flox42C1 clone E3 were treated with RAP (100 nM) or vehicle only (DMSO, 1% v/v) for 4 hr, then washed and cultured for a further ∼44 hr to allow schizont development. The schizonts were Percoll-enriched, returned to culture and allowed to mature for 4–5 hr in the presence of C1, then washed in warm medium without C1 and immediately observed by time-lapse DIC microscopy, taking images at 5 s intervals. Imaging commenced precisely 4 min 20 s following C1 removal. Control-treated parasites (left) underwent normal “explosive” egress with rapid dissemination of daughter merozoites. In contrast, RAP-treated parasites (right) appeared to undergo normal PVM rupture but abortive RBC membrane rupture, with inefficient release of merozoites. Identical results were observed for 3DMSP1flox42C2 clone B51 (not shown). [file mmc5.jpg]

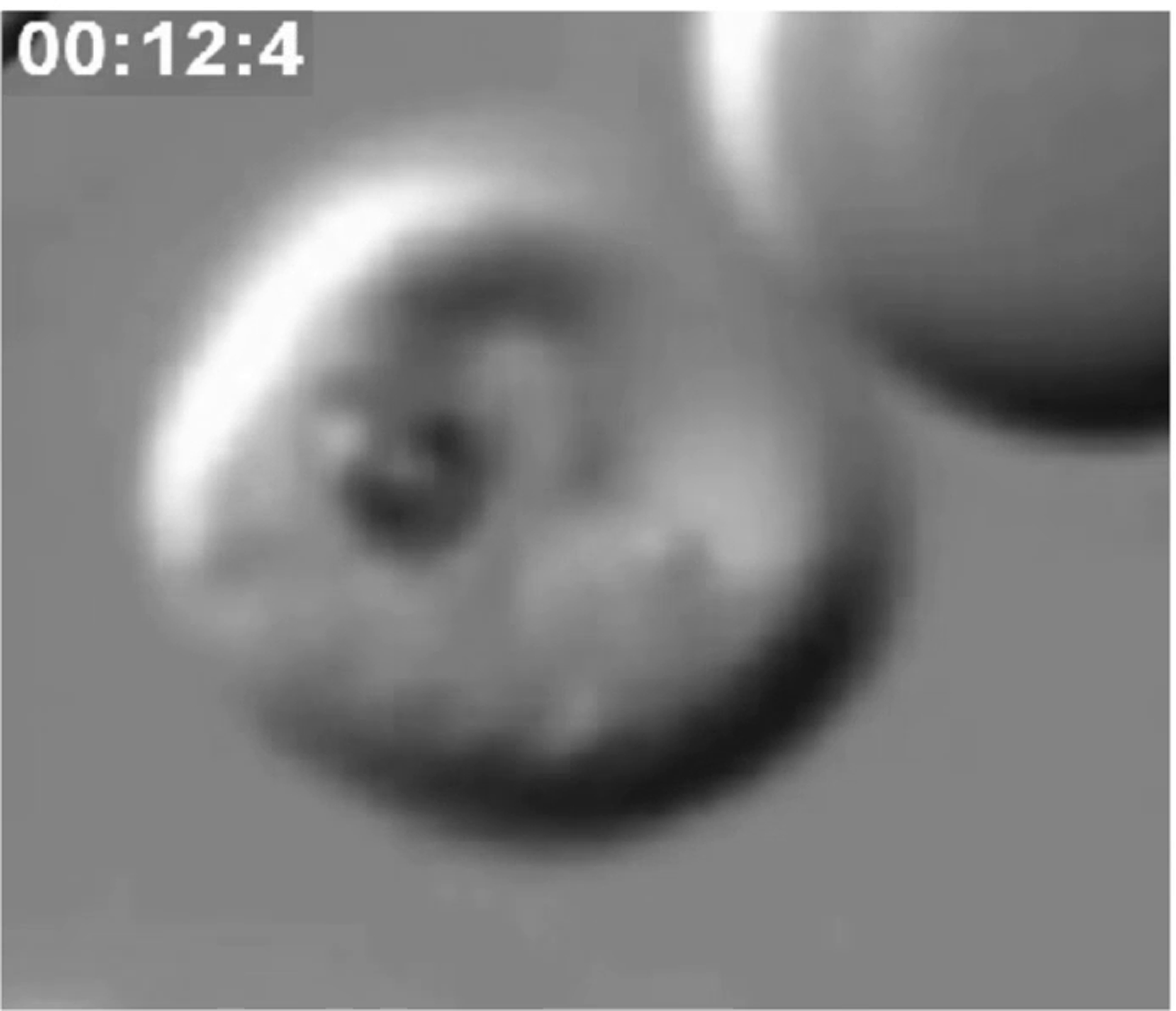

Supplement: Movie S5, Related to Figure 7. P. falciparum Egress May Involve Shear Forces Induced by Movement of Intracellular Merozoites — Time-lapse DIC video microscopy of wild-type 3D7 P. falciparum showing a single egress event selected because it clearly shows that egress is a two-step process. Rupture of the PVM at ∼14.1 min is followed by transient intracellular movement of the merozoites. This allows repeated impingement of the free merozoites upon the inner face of the host erythrocyte membrane before final membrane rupture and egress. Images were taken at 5 s intervals and the movie frame rate is 10 frames/s. [file mmc6.jpg]
