# Supplementary material for: Processing of Plasmodium falciparum Merozoite Surface Protein MSP1 Activates a Spectrin-Binding Function Enabling Parasite Egress from RBCs
Source: Cell Host Microbe. 2015 Oct 14;18(4):433–44. doi: 10.1016/j.chom.2015.09.007 (PMC4608996; doi:10.1016/j.chom.2015.09.007)
Supplement: Document S2. Article plus Supplemental Information [file mmc7.pdf]

Article

Cell Host & Microbe

Processing of *Plasmodium falciparum* Merozoite Surface Protein MSP1 Activates a Spectrin-Binding Function Enabling Parasite Egress from RBCs

Graphical Abstract

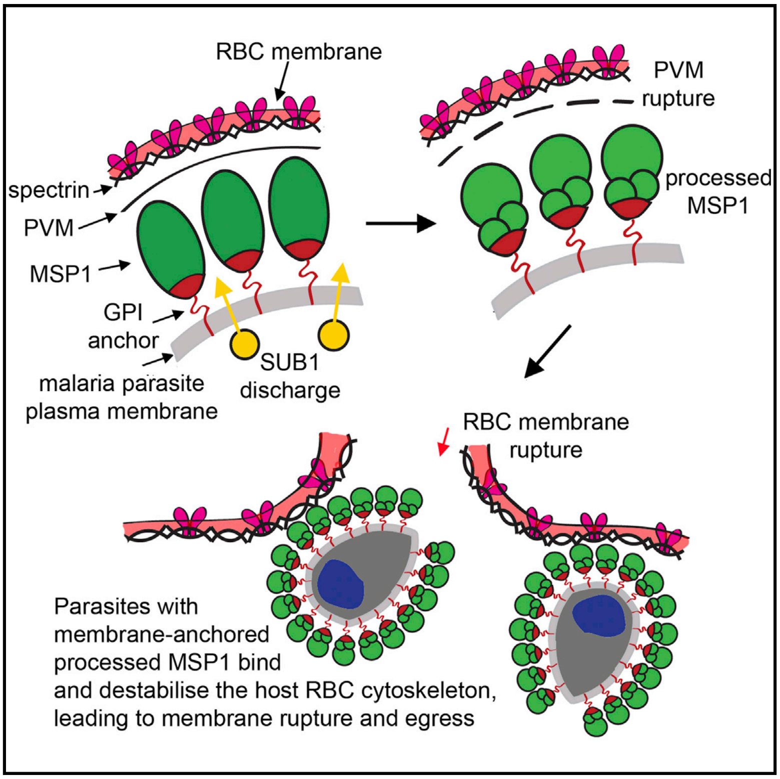

Authors

Sujaan Das, Nadine Hertrich, Abigail J. Perrin, ..., Moritz Treeck, Christian Epp, Michael J. Blackman

Correspondence

mike.blackman@crick.ac.uk

In Brief

Egress from infected RBCs is a critical, but poorly understood, step in the malaria parasite's lifecycle. Das et al. report that just prior to egress, proteolytic processing of parasite surface protein MSP1 activates a spectrin binding function, allowing the intracellular parasite to interact with the RBC cytoskeleton and enabling egress.

Highlights

- Merozoite surface protein MSP1 processing is important for *P. falciparum* viability
- Proteolytic processing activates MSP1's heparin and spectrin-binding functions
- The rate of MSP1 processing governs the kinetics of parasite egress
- Loss of parasite surface MSP1 results in a severe egress defect

Das et al., 2015, Cell Host & Microbe *18*, 433–444
October 14, 2015 ©2015 The Authors
http://dx.doi.org/10.1016/j.chom.2015.09.007

Cell Host & Microbe
Article

Processing of *Plasmodium falciparum* Merozoite Surface Protein MSP1 Activates a Spectrin-Binding Function Enabling Parasite Egress from RBCs

Sujaan Das,[1,6] Nadine Hertrich,[2] Abigail J. Perrin,[3] Chrislaine Withers-Martinez,[1] Christine R. Collins,[1] Matthew L. Jones,[1] Jean M. Watermeyer,[4] Elmar T. Fobes,[2] Stephen R. Martin,[1] Helen R. Saibil,[4] Gavin J. Wright,[3] Moritz Treeck,[1] Christian Epp,[2] and Michael J. Blackman[1,5,*]

[1]The Francis Crick Institute, Mill Hill Laboratory, Mill Hill, London, NW7 1AA, UK
[2]Department für Infektiologie, Parasitologie, Universitätsklinikum Heidelberg, D-69120 Heidelberg, Germany
[3]Wellcome Trust Sanger Institute, Hinxton, Cambridge, CB10 1HH, UK
[4]Department of Crystallography, Birkbeck College, London, WC1E 7HX, UK
[5]Department of Pathogen Molecular Biology, London School of Hygiene and Tropical Medicine, London, WC1E 7HT, UK
[6]Present address: Wellcome Trust Centre for Molecular Parasitology, Glasgow, G12 8TA, UK
*Correspondence: mike.blackman@crick.ac.uk
http://dx.doi.org/10.1016/j.chom.2015.09.007
This is an open access article under the CC BY license (http://creativecommons.org/licenses/by/4.0/).

SUMMARY

The malaria parasite *Plasmodium falciparum* replicates within erythrocytes, producing progeny merozoites that are released from infected cells via a poorly understood process called egress. The most abundant merozoite surface protein, MSP1, is synthesized as a large precursor that undergoes proteolytic maturation by the parasite protease SUB1 just prior to egress. The function of MSP1 and its processing are unknown. Here we show that SUB1-mediated processing of MSP1 is important for parasite viability. Processing modifies the secondary structure of MSP1 and activates its capacity to bind spectrin, a molecular scaffold protein that is the major component of the host erythrocyte cytoskeleton. Parasites expressing an inefficiently processed MSP1 mutant show delayed egress, and merozoites lacking surface-bound MSP1 display a severe egress defect. Our results indicate that interactions between SUB1-processed merozoite surface MSP1 and the spectrin network of the erythrocyte cytoskeleton facilitate host erythrocyte rupture to enable parasite egress.

INTRODUCTION

Malaria is a debilitating and often fatal infectious disease of tropical and subtropical regions. All associated pathology arises from intraerythrocytic replication of the protozoan parasite *Plasmodium*. For most of its erythrocytic life cycle, which lasts ~48 hr in the most dangerous species, *P. falciparum*, the parasite resides within a parasitophorous vacuole (PV), sequestered from the host cell cytosol. Parasite growth leads to formation of a multinucleated schizont. Merozoites, polarized cells specialized for erythrocyte invasion, bud off from the mature schizont. Shortly thereafter, the PV membrane (PVM) ruptures, releasing the now freely mobile progeny merozoites into the residual erythrocyte cytosol. Within seconds, rupture of the host cell membrane allows egress of the merozoites to invade fresh erythrocytes (for a review of egress see Blackman and Carruthers, 2013).

At least 40 proteins localize to the merozoite surface (Cowman et al., 2012). Many of these traffic to the parasite plasma membrane during schizont development, where they are tethered via glycosyl phosphatidylinositol (GPI) anchors or through peripheral associations with GPI-anchored proteins. The most abundant merozoite surface component, a GPI-anchored protein called MSP1, is synthesized as an ~200 kDa protein that in *P. falciparum* associates with at least two other peripheral proteins belonging to the MSP3 and MSP7 families (Kauth et al., 2006; Lin et al., 2014; Pachebat et al., 2001; Trucco et al., 2001). MSP1 is conserved throughout *Plasmodium* and has been scrutinized as a result of its capacity to induce antibody responses that inhibit parasite replication in vitro or protect in vivo (reviewed by Holder, 2009). Gene targeting experiments suggest that MSP1 is essential in the haploid blood stages (Combe et al., 2009; Drew et al., 2004; O'Donnell et al., 2000), but *msp1* null mutants could not be established so these studies provided little insight into MSP1 function. Bioinformatic analyses have been similarly uninformative, since MSP1 has no orthologs outside *Plasmodium* and structural information is sparse. The merozoite surface location of MSP1 has provoked speculation that it functions in erythrocyte invasion. Supporting this are reports that MSP1 binds to erythrocyte glycophorin A (Baldwin et al., 2015; Su et al., 1993), Band 3 (Goel et al., 2003; Li et al., 2004), and heparin-like molecules (Boyle et al., 2010; Zhang et al., 2013), while heparin and related polysaccharides block invasion by *P. falciparum* merozoites (Boyle et al., 2010; Clark et al., 1997; Crick et al., 2014; Kulane et al., 1992; Zhang et al., 2013). However, it remains to be demonstrated that MSP1 plays a primary role in invasion, and a mechanistic understanding of MSP1 function is lacking.

Minutes before egress, a serine protease called SUB1 is discharged from merozoite secretory organelles into the PV lumen, where it cleaves MSP1 and its partner proteins (Koussis et al., 2009; Silmon de Monerri et al., 2011; Yeoh et al.,

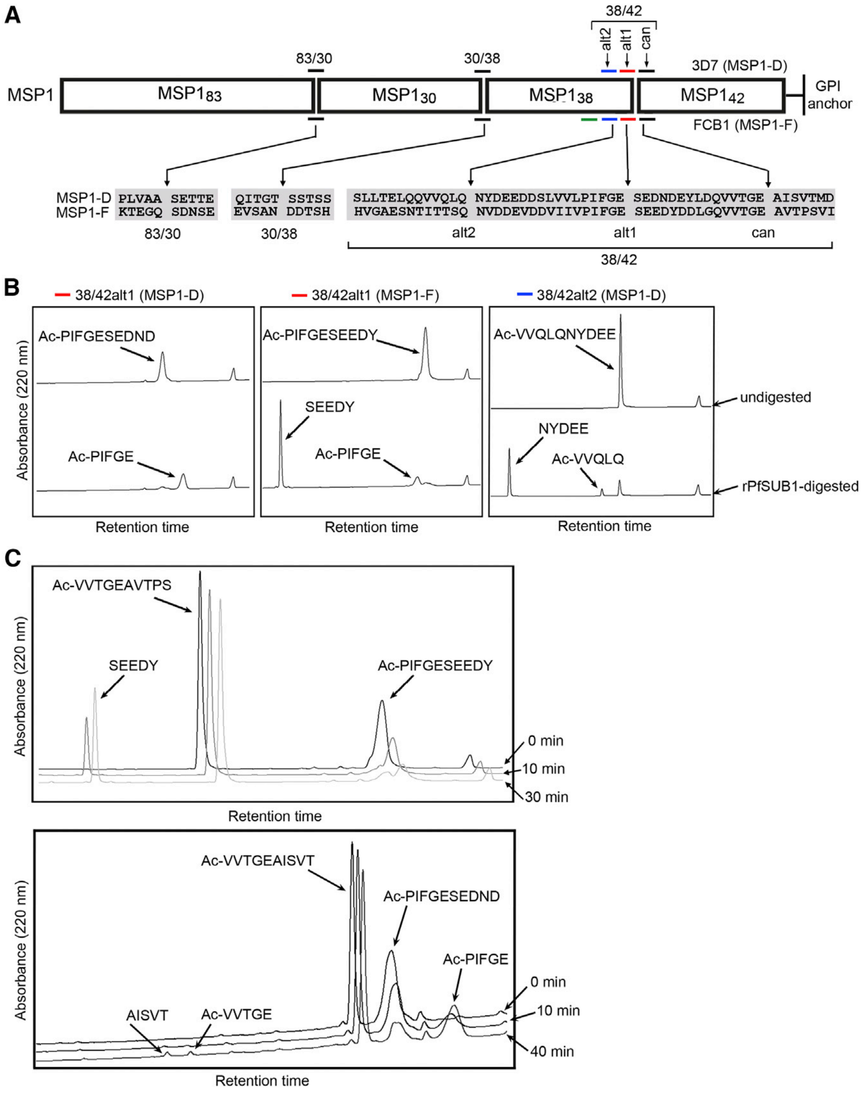

Figure 1. Alternative 38/42 Processing Sites in MSP1

(A) *P. falciparum* MSP1 and primary processing products. Known and predicted PfSUB1 cleavage sites in MSP1-D and MSP1-F (colored horizontal bars), above an alignment of flanking sequences, with experimentally confirmed cleavage sites arrowed and indicated by gaps. The 38/42 region contains the canonical cleavage site (can) as well as two additional sites (alt1 and alt2) confirmed in this work. MSP1-F contains a further predicted 38/42 site (HVGAE↓SNTIT; green bar) but this study found no evidence for cleavage at that site.

(B) Cleavage by rPfSUB1 of peptides based on alternative 38/42 processing sites. RP-HPLC elution profiles of N-acetylated decapeptides before or after incubation with rPfSUB1. Parental peptide peaks diminished over time, with concomitant increase in the indicated products. In the case of Ac-PIFGESEDND the C-terminal cleavage product was too hydrophilic to bind to the RP-HPLC column. The small peak near the end of each chromatogram that does not alter with time represents elution of detergent from the digestion buffer.

(C) The 38/42alt1 peptides are better substrates than the canonical 38/42 site peptides. Equimolar mixtures of peptides based on the canonical 38/42 and 38/42alt1 sites in MSP1-F and MSP1-D were incubated with rPfSUB1 and the peak area for substrate and product(s) monitored with time. Initial cleavage rates were compared after no more than 10% of the fastest cleaved peptide had been hydrolyzed, though for clarity extended digestions are also shown. Cleavage of both 38/42alt1 peptides occurred at least 6.7 times faster than cleavage of the corresponding canonical 38/42 site peptide. See also Figure S1.

2007). *P. falciparum* MSP1 is converted in this primary processing step into four fragments, which initially remain in a non-covalent complex on the merozoite surface (Holder et al., 1987; McBride and Heidrich, 1987). Following egress, MSP1 is further cleaved at a juxtamembrane site by a second parasite protease called SUB2 (Harris et al., 2005), shedding the bulk of the MSP1 complex (Blackman et al., 1991; Riglar et al., 2011). Spatiotemporal regulation of these processing steps is important for parasite viability (Child et al., 2010). Discharge of SUB1—and hence the timing of primary processing—is controlled by a parasite protein kinase (PKG), and inhibition of SUB1 discharge or activity prevents egress (Collins et al., 2013b; Taylor et al., 2010; Yeoh et al., 2007). Despite these insights, the role of MSP1 processing is unknown and a picture of how events following SUB1 discharge lead to rupture of the bounding membranes and erythrocyte cytoskeleton has yet to be established.

Here we show that processing by SUB1 enables MSP1 to interact with the host cell cytoskeleton to play a previously unsuspected role in egress.

RESULTS

Alternative SUB1 Processing Sites in MSP1

P. falciparum MSP1 is a polymorphic protein that exists in two major isoforms, typified by those of the 3D7 and FCB1 parasite isolates. N-terminal sequencing has mapped three positionally conserved primary processing sites in each of these MSP1 isoforms (Blackman et al., 1991; Cooper and Bujard, 1992; Heidrich et al., 1989; Koussis et al., 2009; Stafford et al., 1994). The sites are referred to as 83/30, 30/38, and 38/42, after the approximate masses of the cleavage products (Figure 1A). While all are cleaved by *P. falciparum* SUB1 (PfSUB1), they are structurally distinct, consistent with evidence that PfSUB1 accommodates flexibility in its recognition motif (Withers-Martinez et al., 2012). Only the 38/42 site (i.e., that closest to the C terminus of MSP1) shows significant similarity between 3D7 MSP1 (MSP1-D) and FCB1 MSP1 (MSP1-F) (Figure 1A). Cleavage at the 38/42 site is a rate-limiting processing step (Child et al., 2010), implying special importance.

Since the identification of SUB1 as the enzyme responsible for MSP1 processing, the possibility of additional processing sites

has not been explored. PfSUB1 substrate recognition is dominated by a preference for an aliphatic residue at the P4 position (numbering according to (Schechter and Berger, 1967), a small uncharged residue at P2, a polar residue at P1, and acidic residues at one or more of the P1′–P5′ positions (Withers-Martinez et al., 2012). In early work examining cleavage of recombinant MSP1-D in parasite extracts, Cooper and Bujard (1992) identified two additional cleavage sites adjacent to the canonical 38/42 site, suggesting redundancy. These motifs (VVQLQ↓NYDEE and PIFGE↓SEDND in MSP1-D), which are partially conserved in MSP1-F (Figure 1A), bear hallmarks of PfSUB1 sites, so we tested whether recombinant PfSUB1 (rPfSUB1) could cleave peptides based on them. All were cleaved at their central bond (Figure 1B), suggesting that both the alternative 38/42 sites in MSP1-D, and at least one of the alternative sites in MSP1-F, might be authentic processing sites. As both lie close to the canonical 38/42 site, they are referred to as the 38/42alt1 and 38/42alt2 sites (Figure 1A). In kinetic assays, the 38/42alt1 peptides from both MSP1 isoforms were cleaved ~7-fold faster than the respective canonical 38/42 peptides (Figure 1C), an observation important for subsequent work.

Mutation of MSP1 Prevents PfSUB1-Mediated Processing In Vitro

To begin to address the importance of MSP1 processing, mutations were introduced into a recombinant product called Fwt heterodimer (Kauth et al., 2003), which comprises the two "halves" of MSP1-F refolded into a stoichiometric complex. Substitution of the P2 and P2′ positions at the 83/30 site (mutant Fmut83/30; Figure S1A) ablated cleavage by rPfSUB1 at this position (Figure S1B). Similarly, a recombinant full-length MSP1-F called Fwt (Kauth et al., 2006) with P4 and P2 substitutions at the 30/38 site (mutant Fmut30/38; Figure S1A) was refractory to cleavage at this site (Figure S1C). This showed that appropriate mutations prevent processing and indicated an absence of alternative sites at the 83/30 and 30/38 positions in MSP1-F.

To examine the potential for preventing cleavage at the 38/42 sites, we produced further Fwt heterodimer mutants designed to block cleavage at one or more of these sites, and at a third putative alternative 38/42 site unique to MSP1-F (Figure 1A). Cleavage within the 38/42 region was abolished by simultaneous mutation of the canonical, alt1, alt2, and putative third alternative sites, but mutagenesis of only one site, or two sites together, or the canonical and alt1 sites plus the putative third alternative site, was insufficient to block cleavage (Figure S1D).

In view of the special importance of the 38/42 site, this analysis was extended using a full-length recombinant MSP1-D called rMSP1-DCD4wt. Simultaneous mutagenesis of the canonical, alt1, and alt2 sites completely blocked cleavage within the 38/42 region (Figure S1E). Together, these results confirmed the presence of alternative 38/42 sites in both MSP1 isoforms and identified mutations that prevent all PfSUB1-mediated cleavage.

PfSUB1 Processing of an MSP1 Transgene Product Is Important for Parasite Viability

To test whether mutations that prevent processing are tolerated by *P. falciparum*, we adopted two complementary strategies. First, we exploited an episomal transgene expression system (Epp et al., 2008) that allows blasticidin-regulated control of expression levels. Constructs for expression of three forms of MSP1-F (Figure 2A; Figures S2A, and S2B) were transfected into 3D7 *P. falciparum*, then antibodies specific for MSP1-F used to examine transgene expression on the background of endogenous MSP1-D. Parasites harboring a wild-type *msp1-f* transgene (3D7pHBIMFwt), or the same gene with mutations at all putative 38/42 sites (3D7pHBIMFmut38/42), or at all primary processing sites (3D7pHBIMFmutall), correctly expressed the transgene product on developing merozoites at all blasticidin concentrations tested (Figure 2B; Figure S2C). Varying blasticidin levels from 2–15 μg ml^{-1} did not affect growth of the 3D7pHBIMFwt line or parasites harboring a control plasmid, pHBIRH (Figure 2C, top). However, parasites harboring mutant constructs pHBIMFmut38/42 and pHBIMFmutall showed significantly lower growth rates than the 3D7pHBIMFwt line (Figure 2C, bottom). Whereas the 3D7pHBIMFwt line responded to increases in blasticidin concentration by substantially upregulating *msp1-f* transcript levels (Figure 2D; Figure S2D), likely via increases in episome copy number (confirmed by copy number estimation, data not shown), much less upregulation was seen in the mutant lines, indicating an inability to respond to elevated drug concentrations. Since the episomes differed only at the *msp1-f* cleavage sites, these results suggested that expression of cleavage-resistant MSP1, even in the presence of endogenous MSP1, is deleterious.

Processing of MSP1 in the 38/42 Region Is Important for Parasite Viability

In a second approach to evaluating the importance of PfSUB1-mediated MSP1 processing, we sought to modify the endogenous *msp1* locus using homologous recombination to introduce mutations that prevent processing within the 38/42 region (the importance of processing at the 83/30 and 30/38 sites was not further examined). Our approach used a previously described strategy (Child et al., 2010) in which we transfected 3D7 parasites with constructs containing targeting sequence fused to synthetic "recodonized" sequence encoding a chimeric MSP1 C-terminal domain (Figure 3A). Integration produces a chimeric gene, the product of which can be distinguished from unmodified MSP1-D by its reactivity with the MSP1-F-specific monoclonal antibody (mAb) 111.4. Integration thus epitope tags the gene.

Four integration constructs were initially generated (Figure 3A). Construct pHH1MSP1chim_wt was designed to replace the 3′ region of the *msp1* ORF with the chimeric sequence but leave the 38/42 processing sites unaltered. It thus acted as a control for all other genetic experiments. Constructs pHH1MSP1chim_can and pHH1MSP1chim_alt1 were identical to pHH1MSP1chim_wt except that they were designed to introduce di-leucine mutations at the P2 and P1 positions of the canonical 38/42 site or the 38/42alt1 site, respectively; these substitutions blocked processing of recombinant MSP1 (Figure S1). Construct pHH1MSP1chim_can+alt1 was designed to introduce both these sets of substitutions upon integration, thus blocking cleavage at both the canonical and 38/42alt1 sites. Parasites independently transfected with the constructs were subjected to drug cycling (growth in the absence then presence of WR99210) to select for integration. PCR analysis detected integration of all constructs by drug cycle 2 (data not shown),

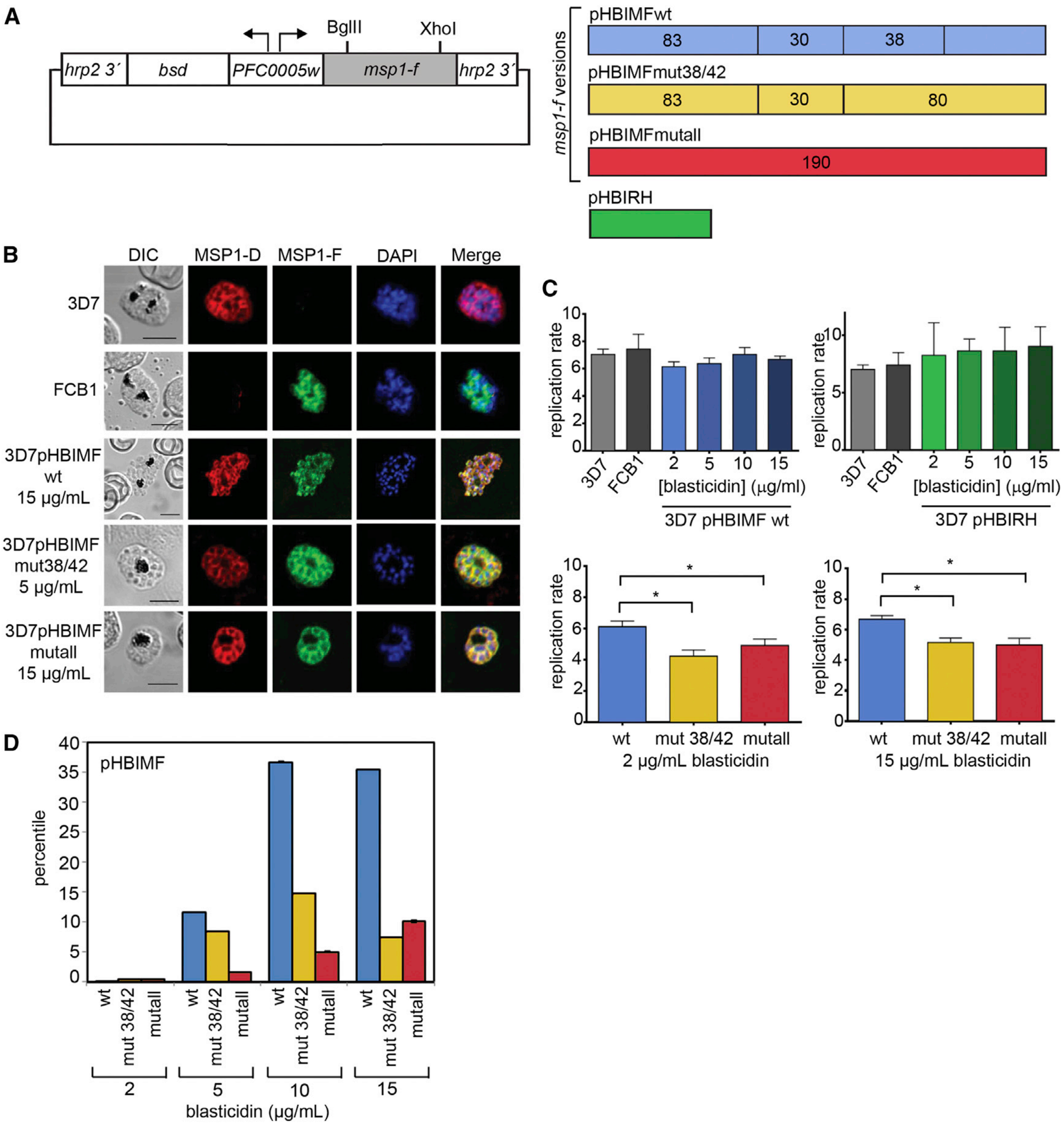

Figure 2. Episomal Expression of Cleavage-Resistant MSP1 Inhibits *P. falciparum* Growth

(A) Blasticidin-regulated co-selection episome. A bi-directional *P. falciparum* promoter (the intron of PlasmoDB: PFC0005w) drives expression of the blasticidin-S-deaminase gene (*bsd*) and *msp1-f* transgene. The *hrp2* gene 3′ UTR controls transgene transcript termination and polyadenylation. Three variants were used, expressing wild-type *msp-1f* (pHBIMFwt, blue), or with mutations at all four known and putative 38/42 sites, (pHIBMFmut38/42, yellow; same mutations as Fmut38/42triple, Figure S1A) or mutations at all primary processing sites (pHBIMFmutall, red; same as mutant Fmutall, Figure S1A). All *msp1-f* sequences included the GPI anchor sequence. Increasing blasticidin concentration selects for parasites harboring multi-copy concatamers to maintain drug resistance, leading to increased *msp1-f* expression. A construct containing the *Renilla* luciferase gene (pHBIRH, green) was used as control.

(B) Immunofluorescence analysis (IFA) of parental 3D7 and FCB1 schizonts, as well as 3D7 schizonts harboring the constructs in the indicated concentrations of blasticidin. Parasites were probed with MSP1 isoform-specific antibodies. Merged signals include that of the DNA dye 4,6-diamidino-2-phenylindole (DAPI, blue). Scale bar, 5 μm.

(C) Quantification by FACS of parasite replication over a single erythrocytic cycle. Top: no significant differences between parental parasites and the transgenic 3D7pHBIMFwt and 3D7pHBIRH lines. Bottom: replication of the 3D7pHBIMFwt line compared to the 3D7pHIBMFmut38/42 and 3D7pHBIMFmutall lines expressing mutant MSP1-F, at similar blasticidin concentrations. Columns show mean values of >3 biological replicates. Error bars, SEM. Statistically different growth rates are indicated (*$p < 0.05$; **$p < 0.01$. Kruskal-Wallis test).

(D) Transgene RNA transcript levels measured by qRT-PCR, as a percentile of endogenous *msp1-d* transcript levels (100%). SEM values in all cases were <0.1%. See also Figure S2.

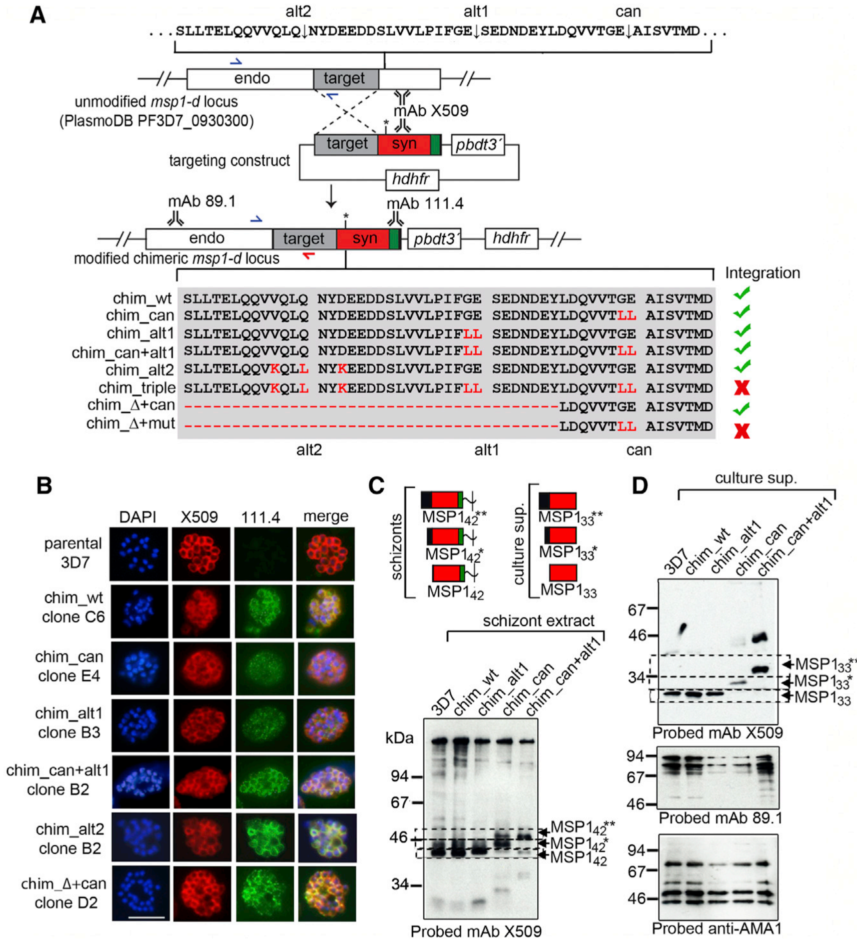

Figure 3. Mutations that Prevent All Processing in the 38/42 Region Are Deleterious

(A) Top: modification of the *P. falciparum msp1-d* locus by single-crossover homologous recombination. The targeting region (gray) incorporated into the integration constructs was fused just upstream of the 38/42 region to recodonized sequence (red/green, syn) encoding the rest of the ORF. In all except pHH1MSP1chim_wt, this incorporated mutations and/or deletions to block processing at one or more 38/42 cleavage sites (asterisk). The 3′ end of the recodonized sequence (green) contained the MSP1-F-specific mAb 111.4 epitope. Positions of hybridization of primers used for diagnostic PCR are indicated (blue and red half arrows). Integration replaced the *msp1* 3′ UTR with that of the *P. berghei* dihydrofolate reductase gene (*pbdt*). The *hdhfr* cassette confers resistance to WR99210. Integration produces a modified locus encoding a chimeric MSP1 recognized by both mAb X509 and mAb 111.4. Bottom: substitutions or deletions (hyphens) introduced by the constructs are indicated (red). Green tick, successful integration. Red cross, no integration detected.

(B) IFA of schizonts of the parental and transgenic *P. falciparum* clones. All transgenics reacted with mAb 111.4. Scale bar, 5 μm.

(C) Top: schematic of the $MSP1_{42}$ fragment (GPI anchored) and the slightly larger $MSP1_{42}{}^{*}$ and $MSP1_{42}{}^{**}$ species predicted to result from ablation of cleavage at the canonical and canonical plus 38/42alt1 sites, respectively. The $MSP1_{33}$ and modified $MSP1_{33}{}^{*}$ and $MSP1_{33}{}^{**}$ fragments, derived from cleavage by SUB2 within $MSP1_{42}$, $MSP1_{42}{}^{*}$, and $MSP1_{42}{}^{**}$, respectively, are also depicted. Bottom: western blot shows differences (highlighted, dotted lines) in migration of the wild-type and modified $MSP1_{42}$ forms.

(D) Western blot of culture supernatants shows differences in migration of $MSP1_{33}$, $MSP1_{33}{}^{*}$, and $MSP1_{33}{}^{**}$ (highlighted as above). As controls, supernatants were probed with mAb 89.1, which recognizes $MSP1_{83}$, or antibodies to an irrelevant shed parasite protein, AMA1. See also Figure S3.

and clones derived from the drug-resistant lines were recognized by mAb 111.4, confirming correct integration (Figure 3B, top five rows). Sequencing of PCR products amplified from the modified *msp1* locus of the clones confirmed the presence of the mutations (data not shown).

The transgenic clones grew normally, indicating no effects of the mutations on viability (data not shown). To assess the impact on MSP1 processing, schizont extracts and culture supernatants containing shed MSP1 fragments were examined by western blot. This showed a shift in migration of the $MSP1_{42}$ cleavage product in the chim_can and chim_can+alt1 clones (Figure 3C), with production of progressively larger fragments termed $MSP1_{42}{}^{*}$ and $MSP1_{42}{}^{**}$. This was consistent with blockade of cleavage at the canonical site (chim_can) or at both modified sites (chim_can+alt1), resulting instead in cleavage at the 38/42alt1 site or the 38/42alt2 site, respectively. Examination of culture supernatants (Figure 3D) showed increases in the mass of the shed $MSP1_{33}$ fragment in the chim_can and chim_can+alt1 clones, again consistent with ablation of processing at the canonical 38/42 site, or both sites, respectively. To confirm the site of cleavage when processing at both the canonical and 38/42alt1 sites was prevented, we purified the $MSP1_{33}{}^{**}$ species from culture medium of a chim_can+alt1 clone (Figure S3). Edman degradation identified its N terminus as NYDEE, confirming the 38/42alt2 cleavage site and proving the presence of alternative, redundant 38/42 cleavage sites in MSP1-D. The lack of a growth defect in the chim_can, chim_alt1, and chim_can+alt1 parasite clones proved that cleavage at the canonical or 38/42alt1 sites is not essential; blocking cleavage at one or both positions simply shifted cleavage to an alternative available 38/42 site.

To examine the effects of blocking processing at all three 38/42 positions, four additional transfection constructs were generated (Figure 3A). Constructs pHH1MSP1chim_alt2 and pHH1MSP1chim_triple were designed to introduce mutations that block cleavage at the 38/42alt2 site or all three 38/42 sites respectively (Figure S1). In addition, pHH1MSP1chim_Δ+can and pHH1MSP1chim_Δ+mut were designed to delete a 69-residue predicted unstructured (data not shown) segment of MSP1 sequence that encompasses both the 38/42alt1 and 38/42alt2 sites. Construct pHH1MSP1chim_Δ+can was designed to leave the canonical 38/42 site unaltered, whereas pHH1MSP1chim_Δ+mut would additionally render this site non-cleavable. Both pHH1MSP1chim_alt2 and pHH1MSP1chim_Δ+can rapidly integrated (data not shown), and the resulting

parasite clones showed the expected reactivity with mAb 111.4 (Figure 3B, bottom 2 rows). In contrast, despite five independent transfection experiments, each with extended periods of drug cycling, integration of pHH1MSP1chim_triple and pHH1MSP1chim_Δ+mut was never detected. Since, aside from the mutations unique to these constructs, they were identical to the other six constructs that readily integrated, this result suggested that cleavage of at least one position within the 38/42 region of MSP1 is important for parasite viability.

Processing of MSP1 Alters Its Secondary Structure and Activates Spectrin and Heparin-Binding Activity

Size-exclusion chromatography of rPfSUB1-cleaved rMSP1-DCD4wt as well as a similar protein lacking the CD4 tag (rMSP1-Dwt; Figure S1E) showed that, like parasite MSP1 (McBride and Heidrich, 1987), the processed products remain associated under non-denaturing conditions (Figure S4A). This encouraged us to use the recombinant proteins to examine the structural consequences of cleavage. Circular dichroism (CD) of intact and rPfSUB1-processed rMSP1-Dwt, as well as of a mutant (rMSP1-Dmut) that was refractory to cleavage in the 38/42 region (Figure S1E), showed that processing altered the secondary structure of both proteins (Figure 4A). These changes were less extensive in rMSP1-Dmut, indicating that cleavage at the 38/42 region contributed to the conformational rearrangements.

MSP1 has been implicated in interactions with erythrocyte surface heparin-like polysaccharides, so we compared the capacity of intact and rPfSUB1-cleaved rMSP1-Dwt to bind to immobilized heparin. Cleaved rMSP1-Dwt showed ~4-fold higher binding than intact protein, which was reduced by soluble heparin (Figure 4B). We next examined the ability of rMSP1-Dwt to bind to intact erythrocytes. No binding was detected (data not shown). However, permeabilized erythrocytes incubated with cleaved rMSP1-Dwt showed an ~3.3-fold more intense IFA signal than cells exposed to intact rMSP1-Dwt (Figure 4C), suggesting that cleavage enhanced binding to an intraerythrocytic component. This was confirmed in pull-down assays using inside-out erythrocyte ghost vesicles (IOVs) (Figure 4D), as well as by immunoEM analysis of erythrocyte cytoskeletons (Figure 4E; Figure S4D), which in both cases showed preferential binding of cleaved rMSP1-Dwt. To determine the target(s) of binding, we probed SDS PAGE-fractionated erythrocyte ghosts in overlay assays with intact or rPfSUB1-processed rMSP1-Dwt. Cleaved rMSP1-Dwt bound exclusively to a Triton X-100-insoluble doublet migrating at the positions of α- and β-spectrin, the dominant components of the cytoskeleton (Figure 4F). This was confirmed by probing purified spectrin "spiked" with irrelevant proteins (Figure 4G). No binding was observed for intact or rPfSUB1-treated rMSP1-DCD4mut (Figure 4H), showing that cleavage within the 38/42 region—already shown to be important for parasite viability—was required for binding to spectrin.

PfSUB1-Mediated Processing of MSP1 Plays a Role in Egress

The erythrocyte cytoskeleton lies beneath the cell membrane so merozoites are unlikely to contact it during invasion. However, intracellular merozoites impinge on the inner face of the host cell membrane in the brief period between PVM rupture and egress (e.g., Glushakova et al., 2009), so we explored the possibility that direct interactions between processed merozoite surface MSP1 and host cell spectrin might play a part in egress. For this, we returned to the chim_Δ+can mutant (Figure 3) in which an MSP1 segment had been deleted to remove the 38/42alt1 and 38/42alt2 sites entirely, leaving just the canonical 38/42 cleavage site. Since this is a relatively poor substrate for PfSUB1 (Figure 1C), we predicted that cleavage within the 38/42 region in the chim_Δ+can mutant should be less efficient than in wild-type parasites. To test this, we compared the kinetics of processing in chim_Δ+can parasites with that in chim_wt parasites, which expressed the same chimeric MSP1 but retained all three 38/42 cleavage sites. For these experiments, schizonts were treated with the reversible PKG inhibitor compound 1 (C1), which prevents PfSUB1 discharge, stalling schizont development at the final stage of maturation. MSP1 processing and egress occur within minutes of washing away the inhibitor (Collins et al., 2013b). As shown in Figure 5A, processing of MSP1 in the chim_Δ+can mutant was delayed relative to chim_wt parasites and was characterized by an unusually prominent $MSP1_{38+42}$ processing intermediate. Comparison of the kinetics of chim_Δ+can and chim_wt egress by time-lapse microscopy showed a reproducible delay in egress in the chim_Δ+can parasites following C1 removal (Figure 5B; Movie S1), mirroring the delay in MSP1 processing. This was confirmed in further experiments in which the clones were imaged simultaneously following fluorescent labeling of one population to identify it (Movie S2; Figure S5). Since the chim_Δ+can and chim_wt parasites differed only by the presence or absence of an MSP1 segment encompassing the 38/42alt1 and alt2 cleavage sites, these results showed that processing of MSP1 regulates the kinetics of egress.

Truncation of MSP1 to Remove Its Merozoite Surface Anchor Produces an Egress Defect

MSP1 is tethered to the merozoite surface via a C-terminal GPI anchor. To further test our model that direct interactions between merozoite-bound MSP1 and the erythrocyte cytoskeleton facilitates egress, we used a recently published conditional strategy to generate *P. falciparum* transgenics in which a 3′ segment of the *msp1* gene could be deleted by rapamycin (RAP)-inducible, Cre recombinase-mediated excision (Figure 6A; Figure S6A) (Collins et al., 2013a). This was predicted to generate a truncated MSP1 that lacked a GPI anchor and so would not be bound to the merozoite surface. Analysis of RAP-treated 3D7MSP1flox42C parasites showed highly efficient excision, resulting in exclusive expression of truncated MSP1 in mature schizonts at the end of the same erythrocytic cycle (Figures 6B and 6C). No effects on merozoite development were discernible. The modified MSP1 was trafficked to the PV as expected for a non-membrane-bound merozoite surface protein (Figures 6C–6D; Figure S6B) but was not present on the surface of free merozoites (Figure 6E). Video microscopy of the RAP-treated 3D7MSP1flox42C schizonts revealed a dramatic egress defect characterized by abortive erythrocyte membrane rupture and trapping of the merozoites in the partially ruptured cell (Figure 6F; Movies S3 and S4). Consistent with this, the RAP-treated mutants displayed a substantially reduced replication rate (Figure 6G and Figure S6C). These results show that MSP1 functions at egress and that this role requires it to be tethered to the merozoite surface.

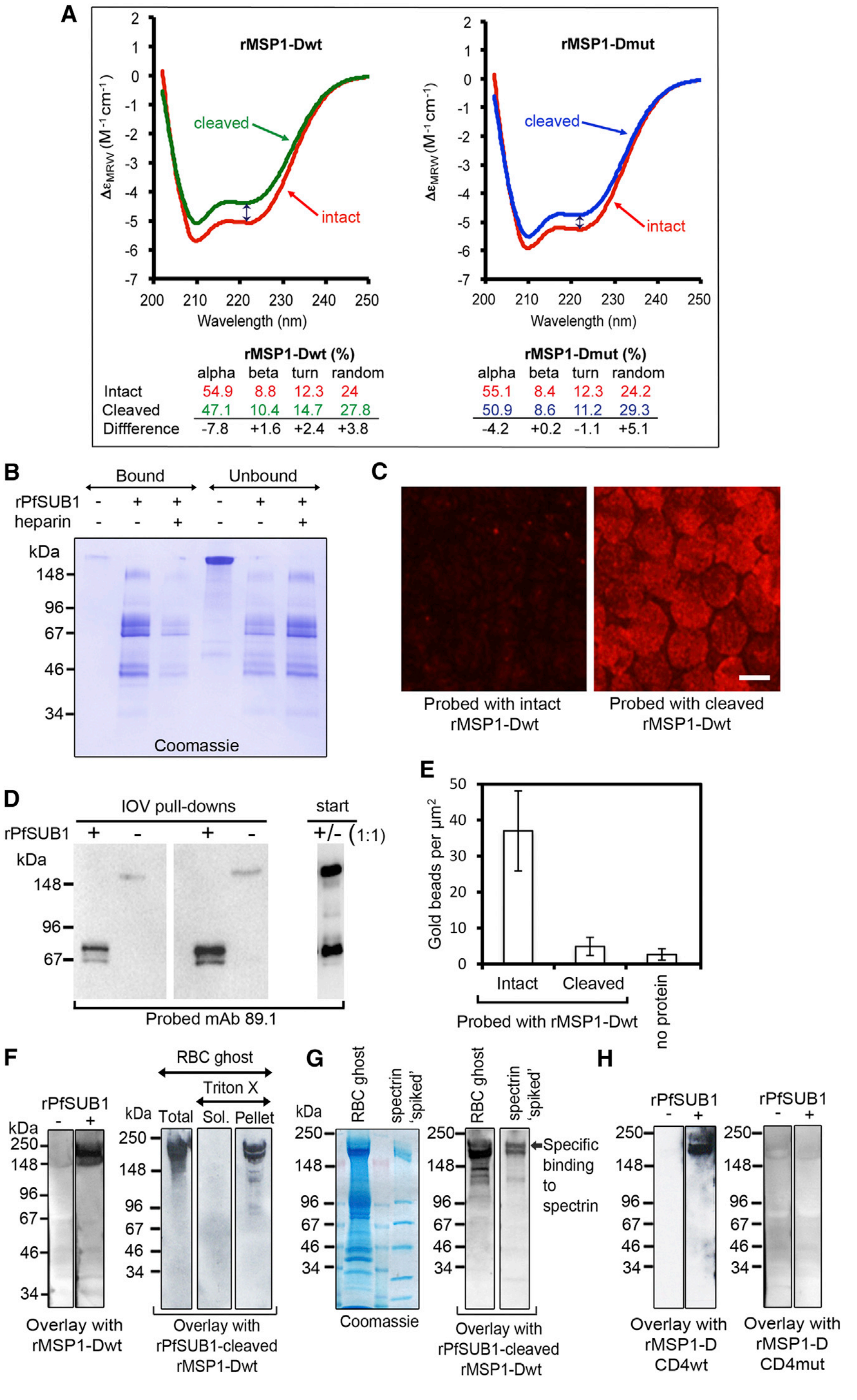

Figure 4. Processing Alters MSP1 Secondary Structure and Activates a Heparin and Spectrin-Binding Activity

(A) Far-UV CD spectra of rMSP1-Dwt and rMSP1-Dmut as a function of molar absorptivity at 37°C. The vertical double arrow at 222 nm (negative minimum for the alpha helix spectrum) highlights the reduction in CD intensity following cleavage, which was 1.4-fold greater for rMSP1-Dwt than for rMSP1-Dmut. Below: secondary structure composition of the intact and processed proteins.

(B) Processing enhances heparin binding. Intact or cleaved rMSP1-Dwt was incubated with heparin agarose ± soluble heparin (1 mg ml^{-1}), then binding assessed by SDS PAGE. Quantification of band intensity (Image Lab) showed that 72.3% ± 6.5% of cleaved rMSP1-Dwt bound heparin agarose but only 18.3% ± 12.1% of intact rMSP1-Dwt (p = 0.006, Student's t test).

(C) Fixed, permeabilized erythrocytes probed with intact or rPfSUB1-cleaved rMSP1-Dwt. Binding was detected by IFA and imaged using equal exposure times. Mean pixel intensity (Adobe Photoshop Histogram tool) was 41.6 ± 4.7 (cleaved) and 12.6 ± 1.4 (uncleaved). Scale bar, 5 μm.

(D) Processing enhances binding to IOVs. Vesicles (~80 μg protein) incubated with intact or rPfSUB1-cleaved rMSP1-Dwt (4 μg) were washed then two different loadings analyzed by western blot in parallel with a 1:1 mixture of the starting protein preparations.

(E) Processing enhances binding to the erythrocyte cytoskeleton. Mean density of bound gold beads following immunoEM of Triton X-100-treated erythrocyte ghosts incubated with intact or rPfSUB1-cleaved rMSP1-Dwt then probed with anti-MSP1 antibodies and 5 nm gold-conjugated secondary antibodies. Error bars, SD.

(F) Overlay assay. Erythrocyte ghosts or Triton X-100-fractionated ghosts were separated by SDS PAGE, transferred to nitrocellulose, then probed with rPfSUB1-cleaved or intact rMSP1-Dwt and binding detected with anti-MSP1 antibodies.

(G) Overlay assay. Erythrocyte ghosts, or purified erythrocyte spectrin (Sigma) mixed with molecular mass marker proteins (GE Healthcare), were subjected to SDS PAGE and either stained or transferred to nitrocellulose and probed as in (F) with rPfSUB1-cleaved rMSP1-Dwt.

(H) Overlay assay. Erythrocyte ghosts probed as in (F) with intact or rPfSUB1-cleaved rMSP1-DCD4wt or rMSP1-DCD4mut. The latter, which is refractory to cleavage in the 38/42 region, did not bind spectrin. See also Figure S4.

Collectively, our findings support the model that processing of MSP1 facilitates host cell membrane rupture, probably through interactions between the mature merozoite surface and the erythrocyte cytoskeleton.

DISCUSSION

We have combined genetic, structural, and functional analysis with microscopic observation of egress to produce evidence that: (1) proteolytic maturation of MSP1 by SUB1 is important for parasite viability; (2) proteolysis alters MSP1 secondary structure, conferring upon it a capacity to bind to both heparin and erythrocyte spectrin; and (3) these functional alterations regulate egress, probably as a result of interactions between MSP1 and the host cell cytoskeleton. The resistance of the erythrocyte membrane to mechanical shear stress is dependent on the structural integrity of its cytoskeleton and in particular its underlying lattice of spectrin tetramers formed by the head-to-head

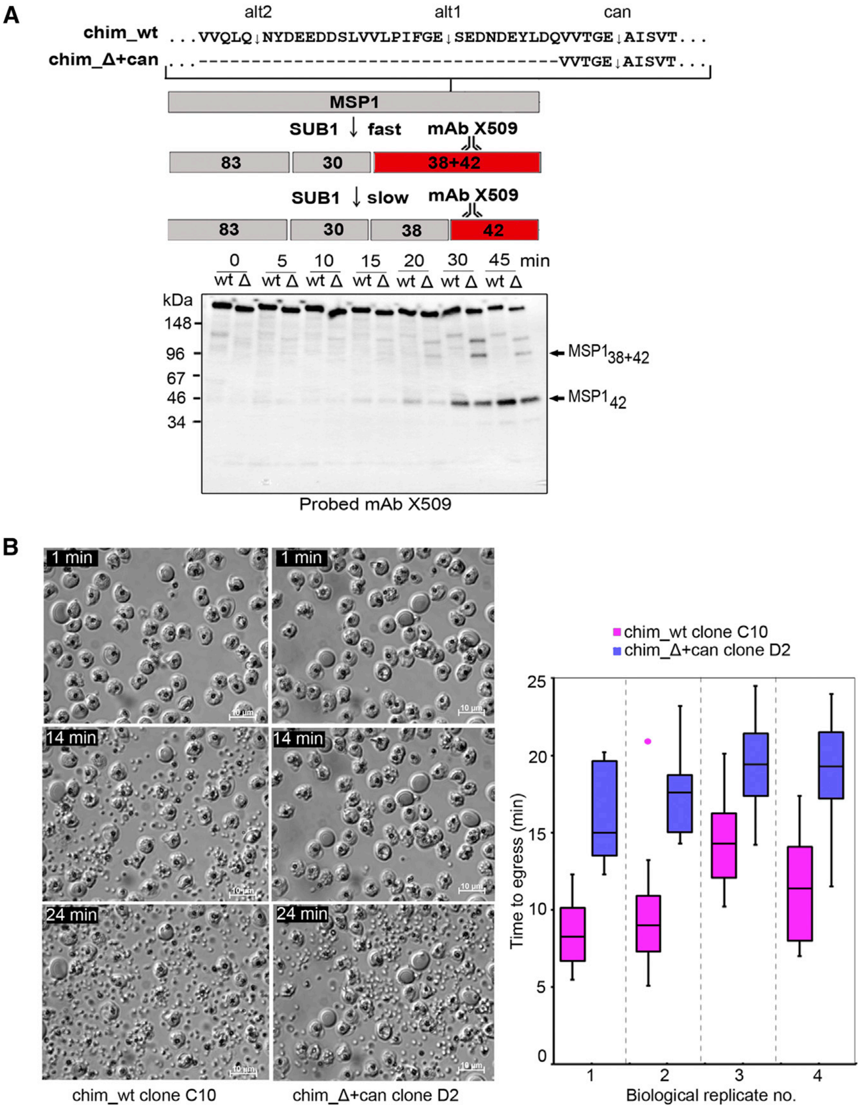

Figure 5. Rate of MSP1 Processing Regulates the Kinetics of Egress

(A) Top: the 38/42 region in chim_Δ+can and chim_wt parasites and the MSP1 processing pathway (Child et al., 2010). Bottom: time course comparing processing of MSP1 from chim_Δ+can and chim_wt clones by western blot. Schizonts were sampled at the indicated times following removal of a C1 block. Note the slightly smaller full-length chim_Δ+can MSP1 due to the 69-residue deletion.

(B) Left: stills from time-lapse microscopy of chim_Δ+can and chim_wt clones (imaging started 4 min 20 s after C1 removal). Right: box plot comparison of time to egress after C1 removal. Data are from 4 independent experiments each assessing 12–24 egress events per clone. Whiskers, range. A single outlier point (>1.5× the interquartile range) indicated. The chim_Δ+can clones showed a mean egress delay of 7.5 ± 1.4 min ($p < 0.005$, Student's t test). Similar results were obtained with two other chim_Δ+can and chim_wt clones (data not shown). See also Figure S5 and Movies S1 and S2.

association of pairs of αβ spectrin heterodimers. The spectrin network is dynamic, accommodating reversible breakage and reformation of the dimer-dimer bonds in response to even moderate shear stress (e.g., Salomao et al., 2006). Shear forces can also result in unfolding of the triple-helical repeat units that comprise α- and β-spectrin, providing additional flexibility (Randles et al., 2007). This dynamic state allows peptides and other small molecules that interfere with tetramer stability (Salomao et al., 2006) or that perturb interactions between spectrin and other cytoskeletal components such as ankyrin (Blanc et al., 2010), protein 4.1R, and actin (An et al., 2007) to destabilize the membrane. SUB1-processed MSP1 may perform an analogous role. We speculate that following PVM breakdown, the diffusive movement of intracellular merozoites impinging upon the inner face of the erythrocyte membrane—well documented by both time-lapse and diffraction phase microscopy (Chandramohanadas et al., 2011; Gilson and Crabb, 2009; Glushakova et al., 2010; Glushakova et al., 2009) (see also Movie S5)—enables merozoite surface-bound MSP1 to bind the spectrin lattice, producing internal shear forces that disrupt the cytoskeleton (Figure 7). This is likely aided by protease activity, perhaps involving host cell calpain-1 (Chandramohanadas et al., 2009) and/or the PfSUB1 substrate SERA6 (Ruecker et al., 2012), since the cysteine protease inhibitor E64 selectively inhibits host cell membrane rupture (e.g., Glushakova et al., 2009). Even localized destabilization of the cytoskeleton may be sufficient to allow egress, since high-speed video microscopy has shown that erythrocyte membrane rupture initiates at a single site; subsequent elastic inversion of the membrane promotes its rapid disintegration (Abkarian et al., 2011; Crick et al., 2013). Interestingly, Herrera et al. (1993) reported spectrin-binding activity for a recombinant MSP1 polypeptide, suggested by those authors as being important for intracellular parasite development. We do not favor that model, since parasites replicate within the PVM, which shields them from the host cytoskeleton. In contrast, the egress delay observed in the chim_Δ+can mutant, and the egress defect (with no effect on schizont development) when MSP1 is conditionally converted to a non merozoite-bound form, implies a role for processed merozoite-bound MSP1 in host cell rupture. Our model explains the defect associated with episomal expression of cleavage-resistant MSP1 (Figure 2), which presumably reduces egress efficiency by reducing the proportion of MSP1 at the merozoite surface able to interact with the host cell cytoskeleton. These data implicate a surface protein in the egress of an intracellular non-viral pathogen. They also provide a plausible mechanistic rationale for the timing of MSP1 processing by SUB1, which "prepares" the merozoites for partaking in their own release.

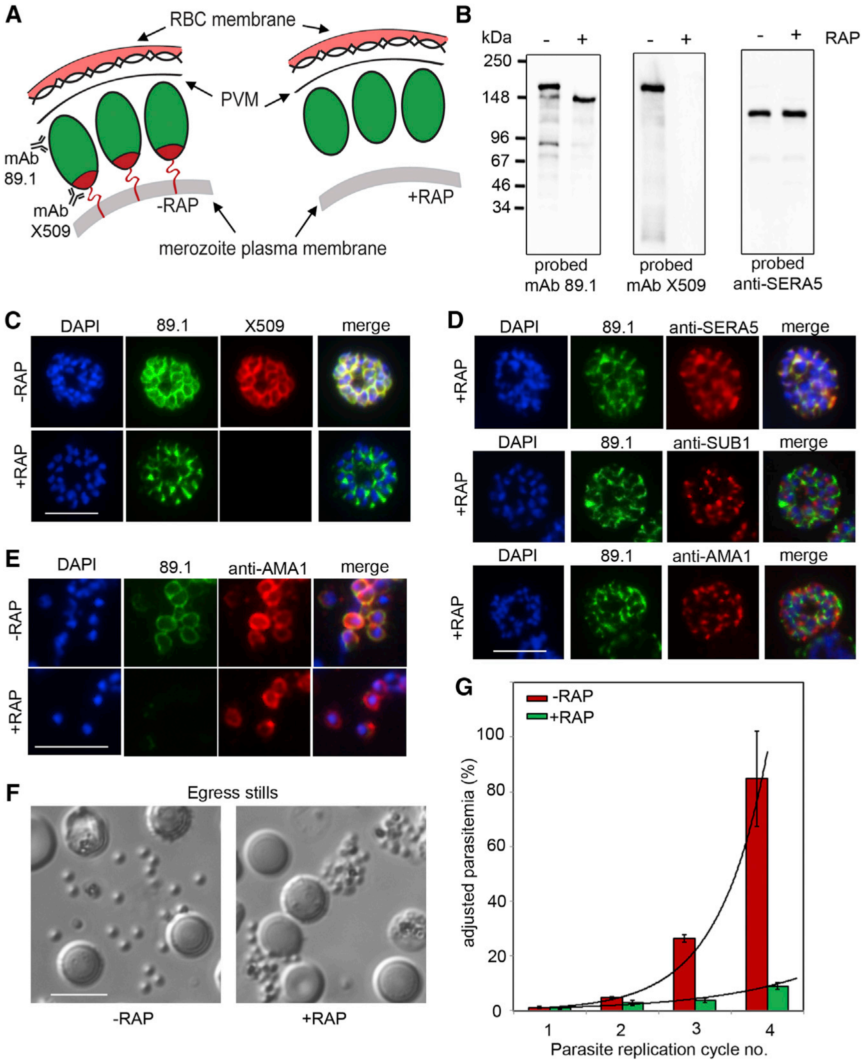

Figure 6. Truncation of MSP1 Produces an Egress Defect

(A) Predicted RAP-induced MSP1 truncation in the 3D7MSP1flox42C clones, showing loss of the GPI anchor and C-terminal domain containing the mAb X509 epitope.

(B) MSP1 truncation confirmed by western blot of 3D7MSP1flox42C1 clone E3 schizonts, 44 hr following treatment ± RAP. The PV protein SERA5 was used as a loading control.

(C) RAP treatment produces a loss of mAb X509 reactivity and a shift in the IFA pattern of MSP1 to one typical of PV proteins, consistent with the predicted truncation. Numbers of DAPI-stained nuclei did not differ between control and RAP-treated schizonts (mean values: 21.2 ± 3.4 and 20.6 ± 4.0 nuclei per schizont, respectively, n = 24).

(D) IFA showing co-localization of truncated MSP1 with SERA5 indicating a PV location. The punctate localization of SUB1 and the microneme protein AMA1 indicates normal organelle biogenesis.

(E) IFA showing lack of surface-bound MSP1 on merozoites of RAP-treated 3D7MSP1flox42C1 clone E3. Antibodies to AMA1 (which is expressed on free merozoites) were used as a control.

(F) Stills from time-lapse DIC microscopic imaging of egress in control and RAP-treated 3D7MSP1flox42C1 clone E3. Scale bar, 10 μm.

(G) Replication rates of RAP- or control-treated 3D7MSP1flox42C1 clone E3. Cultures were passaged at intervals by 10-fold dilution into fresh medium plus erythrocytes as described in Supplemental Experimental Procedures. Observed parasitaemia values were adjusted for these dilutions and are displayed as adjusted values. The plot shows mean values of three biological replicate experiments. Error bars, SEM. The RAP-treated cultures showed an ~2.1-fold reduction in replication rate per cycle, but this was an over-estimate of mutant viability due to rapid expansion of the few (~1%) non-excised parasites in the RAP-treated cultures. See also Figure S6 and Movies S3 and S4.

We do not rule out additional roles for MSP1. A previous report (Combe et al., 2009) showed that knockdown of MSP1 expression in parasite liver stages ablated merozoite formation, suggesting a role in merozoite budding. Additionally, our observation that processing enhances binding to heparin tempts speculation that SUB1 may activate MSP1 to perform a function at invasion. However, the fact that RAP-treated 3D7MSP1flox42C parasites lacking surface-bound MSP1 produce normal numbers of merozoites and replicate in vitro (albeit at a very reduced rate) shows that merozoite surface MSP1 is dispensable for merozoite development and invasion in blood stages. Compounds that inhibit MSP1 processing or that block interactions with spectrin may form the basis of antimalarial drugs that interfere with this key step in the malarial life cycle.

EXPERIMENTAL PROCEDURES

Parasite Culture, Transfection, and Growth Assays

P. falciparum clones FCB1, 3D7, and 1G5DC (Collins et al., 2013a) were maintained in RPMI 1640 medium with Albumax (Invitrogen) and synchronized using standard procedures (Blackman, 1994). Transfection, selection with WR99210 (Jacobus Pharmaceuticals), and cloning was as described (Collins et al., 2013a; Harris et al., 2005). Growth rates were determined by microscopy or fluorescence-activated cell sorting (FACS) as described (Stallmach et al., 2015). Details of transfection constructs based on the pHBIRH episome (Epp et al., 2008) and integration plasmid pMSP1chimWT (Child et al., 2010) are provided in Supplemental Experimental Procedures, as are details of the construct used to flank a segment of the 1G5DC *msp1* ORF with *loxP* sites. For conditional truncation of MSP1 in the 3D7MSP1flox42C clones, synchronous ring-stage parasites were treated for 4 hr with 100 nM RAP (Collins et al., 2013a).

Recombinant Proteins and Antibodies

Monoclonal antibodies 89.1, X509, and 111.4, rabbit polyclonal antibodies and their use in western blot and IFA analysis have been described (Blackman et al., 1991; Child et al., 2010; Ruecker et al., 2012). Production and purification of rPfSUB1, Fwt, and Fwt heterodimer was as described (Kauth et al., 2003, 2006; Withers-Martinez et al., 2012). Mutants of Fwt and Fwt heterodimer were produced using QuikChange II (Agilent) site-directed mutagenesis of parent plasmids. For rMSP1-Dwt, MSP1-D (minus its GPI anchor) was expressed in HEK293E cells (Crosnier et al., 2013); for rMSP1-DCD4wt, it was fused to domains 3 and 4 of rat CD4. Cleavage site mutants were produced by replacing segments of the expression constructs with synthetic gene fragments containing substitutions. The proteins were purified by nickel chelate and size-exclusion chromatography.

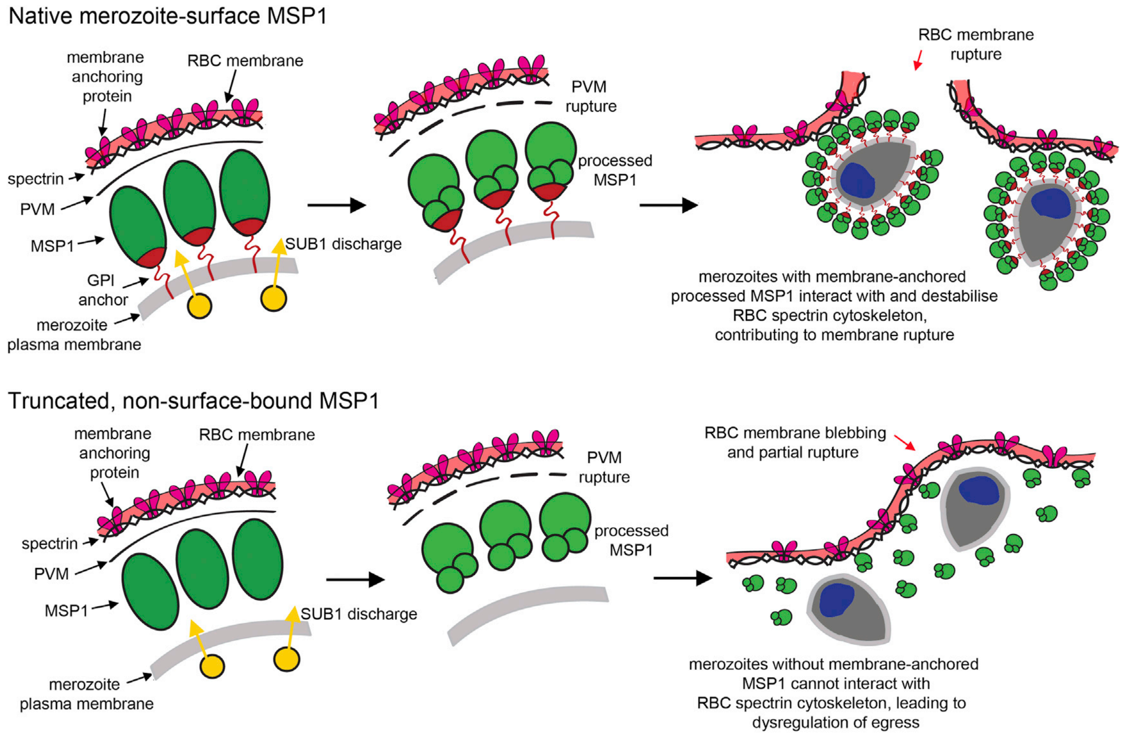

Figure 7. Model for the Role of MSP1 Processing in Egress
See also Movie S5.

Peptide Cleavage Assays and N-Terminal Sequencing

Synthetic peptides were from Biomatik (http://www.biomatik.com). Peptide cleavage assays and product identification by RP-HPLC and mass spectrometry were as described (Koussis et al., 2009; Withers-Martinez et al., 2012). To purify shed MSP1 fragments, 3D7 or chim_can+alt1 schizonts were allowed to undergo egress in protein-free medium then the supernatants fractionated on a Vydac 4.6 × 150 mm 214TP C4 RP-HPLC column. The $MSP1_{33}$ and $MSP1_{33}$** species were identified by western blot then the proteins transferred to PVDF membrane for N-terminal sequencing (PNAC).

Quantitative Real-Time PCR

First strand cDNA synthesis was performed using a SuperScript II First-Strand Synthesis Kit (Invitrogen) according to the manufacturer's instructions. Quantitative real-time PCR (qRT-PCR) was performed using the ABI 7500 sequence detection system and a SensiFASTSYBR Lo-ROX kit (Bioline). Data were analyzed with SDS 1.3.1 software (Applied Biosystems). Transgene expression was displayed as a percentile of endogenous *msp1-d* expression (100%).

Circular Dichroism and Secondary Structure Predictions

Purified rMSP1-Dwt and rMSP1-Dmut (0.156 mg ml^{-1} in 500 μl 25 mM HEPES [pH 7.4], 150 mM NaCl, 15 mM $CaCl_2$) were monitored on a Jasco J-715 spectropolarimeter for 5 hr at 37°C with or without added rPfSUB1 (6 μl at 0.84 mg ml^{-1}). Secondary structure composition was averaged using CONTINLL, SELCON3, and CDSSTR (Sreerama and Woody, 2004). Secondary structure predictions were performed with JPred (http://www.compbio.dundee.ac.uk/www-jpred/).

Heparin-Binding, Overlay Assays, IOV Pulldown Assays and immunoEM

Heparin-agarose beads (Sigma) in assay buffer (25 mM HEPES [pH 7.4], 15 mM NaCl, 0.07% Tween 20) were incubated with intact or cleaved rMSP1-Dwt (50 μl at 0.1 μg μl^{-1}). Control samples were additionally supplemented with heparin sodium salt (1 mg ml^{-1}, Sigma). Following incubation for 20 min at room temperature, supernatants containing unbound proteins were recovered and the beads washed five times with assay buffer. Bound proteins were eluted into 50 μl SDS sample buffer then all samples subjected to reducing SDS-PAGE on a 4%–16% gradient gel. The gel was stained with Coomassie blue, imaged using a BioRad Chemidoc MP system and band intensities estimated using Image Lab software.

Overlay assays to detect binding to SDS-PAGE fractionated human erythrocyte ghost proteins were as described by Herrera et al. (1993). IOVs were prepared using standard procedures from erythrocyte ghosts (see Supplemental Experimental Procedures) and incubated in PBS with intact or rPfSUB1-cleaved rMSP1-Dwt, rMSP1-DCD4wt, or rMSP1-DCD4mut before washing and analysis by western blot, detecting bound proteins with mAb 89.1. For immunoEM analysis, TX-100-treated cytoskeletons immobilized on grid grids were incubated with intact or cleaved rMSP1-Dwt (0.1 μg ml^{-1}) then washed and probed with anti-MSP1 antibodies followed by 5 nm gold-conjugated anti-rabbit IgG, before staining with sodium silicotungstate.

Time-Lapse Microscopy

P. falciparum egress was imaged as described (Collins et al., 2013b), using C1 to synchronize egress. Microscopic DIC images were routinely collected at 5 s intervals for up to 30 min. For comparison of 3D7 chim_Δ+can and 3D7 chim_wt parasites, populations were either alternately imaged or combined in the same microscopy chamber after labeling one mutant with Hoechst 33342 prior to washing away C1. An initial fluorescence image was collected prior to starting the time-lapse DIC imaging, then the fluorescence and first DIC images overlayed to identify labeled cells. Image files were exported as AVI movies using Axiovision 3.1 software. Time to individual egress events was recorded by visual examination of movie frames.

SUPPLEMENTAL INFORMATION

Supplemental Information includes Supplemental Experimental Procedures, six figures, two tables, and five movies and can be found with this article online at http://dx.doi.org/10.1016/j.chom.2015.09.007.

AUTHOR CONTRIBUTIONS

S.D., N.H., and E.T.F. performed the experiments. A.J.P. produced recombinant MSP1. C.W.-M. and S.R.M. performed biophysical analyses. M.L.J. and C.R.C. developed conditional methodologies. H.R.S., G.J.W., M.T., C.E., and M.J.B. supervised the work. S.D., N.H., C.E., and M.J.B. wrote the manuscript.

ACKNOWLEDGMENTS

We are indebted to Fiona Hackett for excellent support with *P. falciparum* culture. This work was supported by the Francis Crick Institute, the MRC (U117532063 to M.J.B. and G1100013 to H.R.S.), the Wellcome Trust (grant no. 098051 to G.J.W.), the German Centre for Infection Research (DZIF) (to C.E.), and EC FP7 contract no. 242095 (EviMalAR). S.D. was in receipt of an EviMalAR PhD studentship. N.H. was supported by a ZMBH fellowship sponsored by the H. Bujard fund. We thank Dominique Soldati-Favre and Tony Holder for membership of S.D.'s Thesis Committee and for invaluable discussions.

Received: August 12, 2015
Revised: September 17, 2015
Accepted: September 18, 2015
Published: October 14, 2015

REFERENCES

Abkarian, M., Massiera, G., Berry, L., Roques, M., and Braun-Breton, C. (2011). A novel mechanism for egress of malarial parasites from red blood cells. Blood *117*, 4118–4124.

An, X., Salomao, M., Guo, X., Gratzer, W., and Mohandas, N. (2007). Tropomyosin modulates erythrocyte membrane stability. Blood *109*, 1284–1288.

Baldwin, M.R., Li, X., Hanada, T., Liu, S.C., and Chishti, A.H. (2015). Merozoite surface protein 1 recognition of host glycophorin A mediates malaria parasite invasion of red blood cells. Blood *125*, 2704–2711.

Blackman, M.J. (1994). Purification of Plasmodium falciparum merozoites for analysis of the processing of merozoite surface protein-1. Methods Cell Biol. *45*, 213–220.

Blackman, M.J., and Carruthers, V.B. (2013). Recent insights into apicomplexan parasite egress provide new views to a kill. Curr. Opin. Microbiol. *16*, 459–464.

Blackman, M.J., Whittle, H., and Holder, A.A. (1991). Processing of the Plasmodium falciparum major merozoite surface protein-1: identification of a 33-kilodalton secondary processing product which is shed prior to erythrocyte invasion. Mol. Biochem. Parasitol. *49*, 35–44.

Blanc, L., Salomao, M., Guo, X., An, X., Gratzer, W., and Mohandas, N. (2010). Control of erythrocyte membrane-skeletal cohesion by the spectrin-membrane linkage. Biochemistry *49*, 4516–4523.

Boyle, M.J., Richards, J.S., Gilson, P.R., Chai, W., and Beeson, J.G. (2010). Interactions with heparin-like molecules during erythrocyte invasion by Plasmodium falciparum merozoites. Blood *115*, 4559–4568.

Chandramohanadas, R., Davis, P.H., Beiting, D.P., Harbut, M.B., Darling, C., Velmourougane, G., Lee, M.Y., Greer, P.A., Roos, D.S., and Greenbaum, D.C. (2009). Apicomplexan parasites co-opt host calpains to facilitate their escape from infected cells. Science *324*, 794–797.

Chandramohanadas, R., Park, Y., Lui, L., Li, A., Quinn, D., Liew, K., Diez-Silva, M., Sung, Y., Dao, M., Lim, C.T., et al. (2011). Biophysics of malarial parasite exit from infected erythrocytes. PLoS ONE *6*, e20869.

Child, M.A., Epp, C., Bujard, H., and Blackman, M.J. (2010). Regulated maturation of malaria merozoite surface protein-1 is essential for parasite growth. Mol. Microbiol. *78*, 187–202.

Clark, D.L., Su, S., and Davidson, E.A. (1997). Saccharide anions as inhibitors of the malaria parasite. Glycoconj. J. *14*, 473–479.

Collins, C.R., Das, S., Wong, E.H., Andenmatten, N., Stallmach, R., Hackett, F., Herman, J.P., Müller, S., Meissner, M., and Blackman, M.J. (2013a). Robust inducible Cre recombinase activity in the human malaria parasite Plasmodium falciparum enables efficient gene deletion within a single asexual erythrocytic growth cycle. Mol. Microbiol. *88*, 687–701.

Collins, C.R., Hackett, F., Strath, M., Penzo, M., Withers-Martinez, C., Baker, D.A., and Blackman, M.J. (2013b). Malaria parasite cGMP-dependent protein kinase regulates blood stage merozoite secretory organelle discharge and egress. PLoS Pathog. *9*, e1003344.

Combe, A., Giovannini, D., Carvalho, T.G., Spath, S., Boisson, B., Loussert, C., Thiberge, S., Lacroix, C., Gueirard, P., and Ménard, R. (2009). Clonal conditional mutagenesis in malaria parasites. Cell Host Microbe *5*, 386–396.

Cooper, J.A., and Bujard, H. (1992). Membrane-associated proteases process Plasmodium falciparum merozoite surface antigen-1 (MSA1) to fragment gp41. Mol. Biochem. Parasitol. *56*, 151–160.

Cowman, A.F., Berry, D., and Baum, J. (2012). The cellular and molecular basis for malaria parasite invasion of the human red blood cell. J. Cell Biol. *198*, 961–971.

Crick, A.J., Tiffert, T., Shah, S.M., Kotar, J., Lew, V.L., and Cicuta, P. (2013). An automated live imaging platform for studying merozoite egress-invasion in malaria cultures. Biophys. J. *104*, 997–1005.

Crick, A.J., Theron, M., Tiffert, T., Lew, V.L., Cicuta, P., and Rayner, J.C. (2014). Quantitation of malaria parasite-erythrocyte cell-cell interactions using optical tweezers. Biophys. J. *107*, 846–853.

Crosnier, C., Wanaguru, M., McDade, B., Osier, F.H., Marsh, K., Rayner, J.C., and Wright, G.J. (2013). A library of functional recombinant cell-surface and secreted P. falciparum merozoite proteins. Mol. Cell. Proteomics *12*, 3976–3986.

Drew, D.R., O'Donnell, R.A., Smith, B.J., and Crabb, B.S. (2004). A common cross-species function for the double epidermal growth factor-like modules of the highly divergent plasmodium surface proteins MSP-1 and MSP-8. J. Biol. Chem. *279*, 20147–20153.

Epp, C., Raskolnikov, D., and Deitsch, K.W. (2008). A regulatable transgene expression system for cultured Plasmodium falciparum parasites. Malar. J. *7*, 86.

Gilson, P.R., and Crabb, B.S. (2009). Morphology and kinetics of the three distinct phases of red blood cell invasion by Plasmodium falciparum merozoites. Int. J. Parasitol. *39*, 91–96.

Glushakova, S., Mazar, J., Hohmann-Marriott, M.F., Hama, E., and Zimmerberg, J. (2009). Irreversible effect of cysteine protease inhibitors on the release of malaria parasites from infected erythrocytes. Cell. Microbiol. *11*, 95–105.

Glushakova, S., Humphrey, G., Leikina, E., Balaban, A., Miller, J., and Zimmerberg, J. (2010). New stages in the program of malaria parasite egress imaged in normal and sickle erythrocytes. Curr. Biol. *20*, 1117–1121.

Goel, V.K., Li, X., Chen, H., Liu, S.C., Chishti, A.H., and Oh, S.S. (2003). Band 3 is a host receptor binding merozoite surface protein 1 during the Plasmodium falciparum invasion of erythrocytes. Proc. Natl. Acad. Sci. USA *100*, 5164–5169.

Harris, P.K., Yeoh, S., Dluzewski, A.R., O'Donnell, R.A., Withers-Martinez, C., Hackett, F., Bannister, L.H., Mitchell, G.H., and Blackman, M.J. (2005). Molecular identification of a malaria merozoite surface sheddase. PLoS Pathog. *1*, 241–251.

Heidrich, H.G., Miettinen-Baumann, A., Eckerskorn, C., and Lottspeich, F. (1989). The N-terminal amino acid sequences of the Plasmodium falciparum (FCB1) merozoite surface antigens of 42 and 36 kilodalton, both derived from the 185-195-kilodalton precursor. Mol. Biochem. Parasitol. *34*, 147–154.

Herrera, S., Rudin, W., Herrera, M., Clavijo, P., Mancilla, L., de Plata, C., Matile, H., and Certa, U. (1993). A conserved region of the MSP-1 surface protein of Plasmodium falciparum contains a recognition sequence for erythrocyte spectrin. EMBO J. *12*, 1607–1614.

Holder, A.A. (2009). The carboxy-terminus of merozoite surface protein 1: structure, specific antibodies and immunity to malaria. Parasitology *136*, 1445–1456.

Holder, A.A., Sandhu, J.S., Hillman, Y., Davey, L.S., Nicholls, S.C., Cooper, H., and Lockyer, M.J. (1987). Processing of the precursor to the major merozoite surface antigens of Plasmodium falciparum. Parasitology *94*, 199–208.

Kauth, C.W., Epp, C., Bujard, H., and Lutz, R. (2003). The merozoite surface protein 1 complex of human malaria parasite Plasmodium falciparum: interactions and arrangements of subunits. J. Biol. Chem. *278*, 22257–22264.

Kauth, C.W., Woehlbier, U., Kern, M., Mekonnen, Z., Lutz, R., Mücke, N., Langowski, J., and Bujard, H. (2006). Interactions between merozoite surface proteins 1, 6, and 7 of the malaria parasite Plasmodium falciparum. J. Biol. Chem. *281*, 31517–31527.

Koussis, K., Withers-Martinez, C., Yeoh, S., Child, M., Hackett, F., Knuepfer, E., Juliano, L., Woehlbier, U., Bujard, H., and Blackman, M.J. (2009). A multifunctional serine protease primes the malaria parasite for red blood cell invasion. EMBO J. *28*, 725–735.

Kulane, A., Ekre, H.P., Perlmann, P., Rombo, L., Wahlgren, M., and Wahlin, B. (1992). Effect of different fractions of heparin on Plasmodium falciparum merozoite invasion of red blood cells in vitro. Am. J. Trop. Med. Hyg. *46*, 589–594.

Li, X., Chen, H., Oo, T.H., Daly, T.M., Bergman, L.W., Liu, S.C., Chishti, A.H., and Oh, S.S. (2004). A co-ligand complex anchors Plasmodium falciparum merozoites to the erythrocyte invasion receptor band 3. J. Biol. Chem. *279*, 5765–5771.

Lin, C.S., Uboldi, A.D., Marapana, D., Czabotar, P.E., Epp, C., Bujard, H., Taylor, N.L., Perugini, M.A., Hodder, A.N., and Cowman, A.F. (2014). The merozoite surface protein 1 complex is a platform for binding to human erythrocytes by Plasmodium falciparum. J. Biol. Chem. *289*, 25655–25669.

McBride, J.S., and Heidrich, H.G. (1987). Fragments of the polymorphic Mr 185,000 glycoprotein from the surface of isolated Plasmodium falciparum merozoites form an antigenic complex. Mol. Biochem. Parasitol. *23*, 71–84.

O'Donnell, R.A., Saul, A., Cowman, A.F., and Crabb, B.S. (2000). Functional conservation of the malaria vaccine antigen MSP-119across distantly related Plasmodium species. Nat. Med. *6*, 91–95.

Pachebat, J.A., Ling, I.T., Grainger, M., Trucco, C., Howell, S., Fernandez-Reyes, D., Gunaratne, R., and Holder, A.A. (2001). The 22 kDa component of the protein complex on the surface of Plasmodium falciparum merozoites is derived from a larger precursor, merozoite surface protein 7. Mol. Biochem. Parasitol. *117*, 83–89.

Randles, L.G., Rounsevell, R.W., and Clarke, J. (2007). Spectrin domains lose cooperativity in forced unfolding. Biophys. J. *92*, 571–577.

Riglar, D.T., Richard, D., Wilson, D.W., Boyle, M.J., Dekiwadia, C., Turnbull, L., Angrisano, F., Marapana, D.S., Rogers, K.L., Whitchurch, C.B., et al. (2011). Super-resolution dissection of coordinated events during malaria parasite invasion of the human erythrocyte. Cell Host Microbe *9*, 9–20.

Ruecker, A., Shea, M., Hackett, F., Suarez, C., Hirst, E.M., Milutinovic, K., Withers-Martinez, C., and Blackman, M.J. (2012). Proteolytic activation of the essential parasitophorous vacuole cysteine protease SERA6 accompanies malaria parasite egress from its host erythrocyte. J. Biol. Chem. *287*, 37949–37963.

Salomao, M., An, X., Guo, X., Gratzer, W.B., Mohandas, N., and Baines, A.J. (2006). Mammalian alpha I-spectrin is a neofunctionalized polypeptide adapted to small highly deformable erythrocytes. Proc. Natl. Acad. Sci. USA *103*, 643–648.

Schechter, I., and Berger, A. (1967). On the size of the active site in proteases. I. Papain. Biochem. Biophys. Res. Commun. *27*, 157–162.

Silmon de Monerri, N.C., Flynn, H.R., Campos, M.G., Hackett, F., Koussis, K., Withers-Martinez, C., Skehel, J.M., and Blackman, M.J. (2011). Global identification of multiple substrates for Plasmodium falciparum SUB1, an essential malarial processing protease. Infect. Immun. *79*, 1086–1097.

Sreerama, N., and Woody, R.W. (2004). Computation and analysis of protein circular dichroism spectra. Methods Enzymol. *383*, 318–351.

Stafford, W.H., Blackman, M.J., Harris, A., Shai, S., Grainger, M., and Holder, A.A. (1994). N-terminal amino acid sequence of the Plasmodium falciparum merozoite surface protein-1 polypeptides. Mol. Biochem. Parasitol. *66*, 157–160.

Stallmach, R., Kavishwar, M., Withers-Martinez, C., Hackett, F., Collins, C.R., Howell, S.A., Yeoh, S., Knuepfer, E., Atid, A.J., Holder, A.A., and Blackman, M.J. (2015). Plasmodium falciparum SERA5 plays a non-enzymatic role in the malarial asexual blood-stage lifecycle. Mol. Microbiol. *96*, 368–387.

Su, S., Sanadi, A.R., Ifon, E., and Davidson, E.A. (1993). A monoclonal antibody capable of blocking the binding of Pf200 (MSA-1) to human erythrocytes and inhibiting the invasion of Plasmodium falciparum merozoites into human erythrocytes. J. Immunol. *151*, 2309–2317.

Taylor, H.M., McRobert, L., Grainger, M., Sicard, A., Dluzewski, A.R., Hopp, C.S., Holder, A.A., and Baker, D.A. (2010). The malaria parasite cyclic GMP-dependent protein kinase plays a central role in blood-stage schizogony. Eukaryot. Cell *9*, 37–45.

Trucco, C., Fernandez-Reyes, D., Howell, S., Stafford, W.H., Scott-Finnigan, T.J., Grainger, M., Ogun, S.A., Taylor, W.R., and Holder, A.A. (2001). The merozoite surface protein 6 gene codes for a 36 kDa protein associated with the Plasmodium falciparum merozoite surface protein-1 complex. Mol. Biochem. Parasitol. *112*, 91–101.

Withers-Martinez, C., Suarez, C., Fulle, S., Kher, S., Penzo, M., Ebejer, J.P., Koussis, K., Hackett, F., Jirgensons, A., Finn, P., and Blackman, M.J. (2012). Plasmodium subtilisin-like protease 1 (SUB1): insights into the active-site structure, specificity and function of a pan-malaria drug target. Int. J. Parasitol. *42*, 597–612.

Yeoh, S., O'Donnell, R.A., Koussis, K., Dluzewski, A.R., Ansell, K.H., Osborne, S.A., Hackett, F., Withers-Martinez, C., Mitchell, G.H., Bannister, L.H., et al. (2007). Subcellular discharge of a serine protease mediates release of invasive malaria parasites from host erythrocytes. Cell *131*, 1072–1083.

Zhang, Y., Jiang, N., Lu, H., Hou, N., Piao, X., Cai, P., Yin, J., Wahlgren, M., and Chen, Q. (2013). Proteomic analysis of Plasmodium falciparum schizonts reveals heparin-binding merozoite proteins. J. Proteome Res. *12*, 2185–2193.

Cell Host & Microbe
Supplemental Information

Processing of *Plasmodium falciparum* Merozoite Surface Protein MSP1 Activates a Spectrin-Binding Function Enabling Parasite Egress from RBCs

Sujaan Das, Nadine Hertrich, Abigail J. Perrin, Chrislaine Withers-Martinez, Christine R. Collins, Matthew L. Jones, Jean M. Watermeyer, Elmar T. Fobes, Stephen R. Martin, Helen R. Saibil, Gavin J. Wright, Moritz Treeck, Christian Epp, and Michael J. Blackman

Supplemental Figures and legends

Figure S1

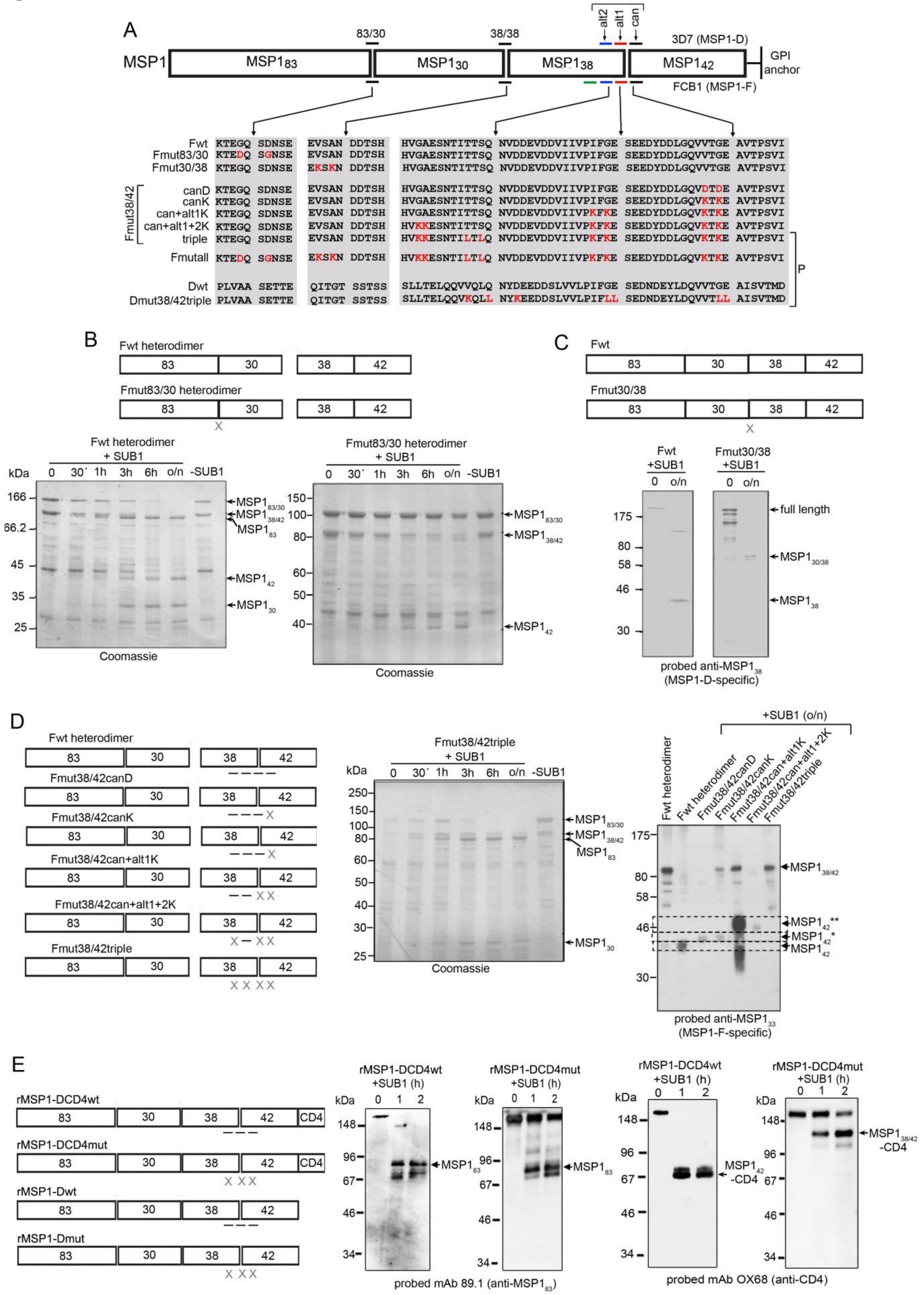

Figure S1, related to Figure 1. Mutagenesis of Processing Sites blocks rPfSUB1-mediated Cleavage of Recombinant MSP1

(A) Schematic of MSP1 processing products and primary processing sites, above a ClustalW2 alignment of flanking sequences in the recombinant wild-type proteins and mutants used in this study. Experimentally confirmed cleavage sites are arrowed and indicated by gaps. Substitutions to block cleavage (red) simultaneously replaced 2 residues at the P4, P2, P1, P2′ or P3′ positions important for recognition by PfSUB1. Mutations additionally studied for impact on parasite growth by transgene expression in the parasite are indicated on the right (P).

(B) Mutagenesis of the 83/30 site blocks PfSUB1-mediated cleavage. Top, schematic of the recombinant Fwt heterodimer and Fmut83/30 heterodimer mutant. Positions of mutations (see panel A) are indicated (grey cross). Below, typical time-courses of digestion of both proteins. For each, 50 µg of protein was incubated with rPfSUB1. Samples taken at intervals were analysed by SDS-PAGE and Coomassie staining alongside samples incubated overnight (o/n) in the absence of rPfSUB1. Positions of parental and product proteins are indicated. Note the lack of digestion of the $MSP1_{83/30}$ fragment in the case of the Fmut83/30 heterodimer mutant.

(C) Mutagenesis of the 30/38 site blocks cleavage. Top, schematic of the Fwt recombinant and Fmut30/38 mutant. Positions of mutations (see panel A) are indicated (grey cross). Below, Western blot analysis of undigested (0) or digested (o/n) proteins. Positions of parental and product proteins are indicated. Note the appearance of a stable $MSP1_{30/38}$ intermediate and absence of a $MSP1_{38}$ product in the case of Fmut83/30.

(D) Mutagenesis of at least three sites is required to block cleavage in the 38/42 region of MSP1-F. Left-hand side, schematics of the Fwt heterodimer and 38/42 region mutants. Positions of known or predicted 38/42 cleavage sites are shown by short horizontal lines, whilst positions of mutations (see panel A) are indicated (crosses). Middle, SDS-PAGE analysis of a time-course of digestion of the Fmut38/42triple mutant, performed as described in (C). Whilst cleavage took place normally at the

83/30 site, the $MSP1_{38/42}$ fragment was completely stable, indicating complete blockade of cleavage in the 38/42 region by the introduced mutations. Right hand side, Western blot analysis of cleavage of the various proteins. Note the increase in the apparent mass of the $MSP1_{42}$ fragments produced when mutations were successively introduced along the 38/42 region. The $MSP1_{42}$, $MSP1_{42}$*, and $MSP1_{42}$** processing products are predicted to result from cleavage at the canonical, alt1, and alt2 38/42 sites of the Fwt heterodimer. Positions of migration of full-length Fwt heterodimer fragments are indicated (with arrows), as are the various processing products.

(E) Mutagenesis of the canonical, alt1 and alt2 38/42 sites in MSP1-D completely blocks cleavage in the 38/42 region. Left-hand side, schematics of rMSP1-DCD4wt and its mutant derivative rMSP1-DCD4mut, which contains mutations at the canonical, alt1, and alt2 38/42 sites (grey crosses; equivalent to mutant Dmut38/42triple, panel A). Proteins rMSP1-Dwt and rMSP1-Dmut were identical except that they lacked the C-terminal CD4 fusion partner. Right hand side, analysis of cleavage. The indicated proteins were incubated with rPfSUB1 for 0-2 h then analysed by Western blot, probing either with monoclonal antibody (mAb) 89.1 which recognises the $MSP1_{83}$ product, or mAb OX68 which recognises the CD4 fusion partner. Cleavage of both proteins (at the 83/30 site) to produce $MSP1_{83}$ occurred as expected. However, whereas cleavage to produce the C-terminal $MSP1_{42}$-CD4 product occurred as expected in rMSP1-DCD4wt, this was completely ablated in rMSP1-DCD4mut due to the mutant 38/42 site being completely refractory to cleavage; a much larger C-terminal fragment ($MSP1_{38/42}$-CD4) was instead produced, likely resulting from cleavage at the 30/38 junction.

Figure S2

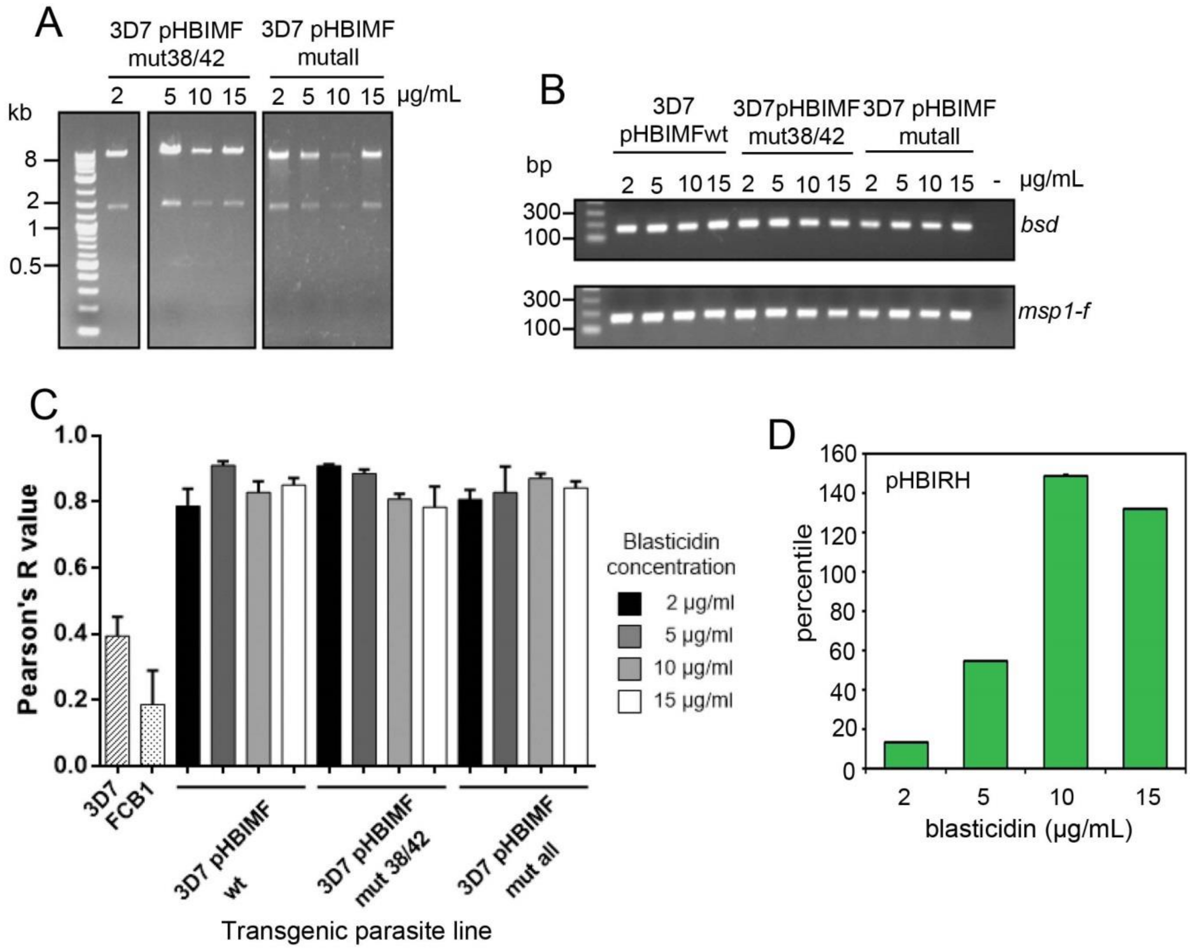

Figure S2, related to Figure 2. Stable Carriage of Episomal Constructs for Transgenic Expression of MSP1 in *P. falciparum*

(A) Plasmid rescue from *P. falciparum* 3D7 parasites stably transformed with pHBIMFmut38/42 or pHBIMFmutall detects no signs of plasmid rearrangement. Genomic DNA was prepared from parasite lines maintained in the presence of the indicated blasticidin concentrations, then episomal plasmids rescued by transformation of *E. coli*. Digestion of DNA from randomly-selected individual colonies with *Bgl II* and *Xho I* reproducibly yielded the expected diagnostic fragments of 8,494 bp and 1,874 bp.

(B) PCR analysis of genomic DNA isolated from the indicated parasite lines grown under varying blasticidin concentrations detected no signs of plasmid rearrangement following carriage in *P. falciparum*. The primer pairs used (bsd3up plus bsd3down, or f83up plus f83down) each specifically

amplify a ~180 bp fragment from the *bsd* and *msp1-f* genes (see *Supplementary Experimental Procedures* for a list of all primers used in this study). The extreme right-hand lane of each gel (labelled "-") is a no DNA template control.

(C) Quantitative analysis shows correct co-localization of endogenous MSP-1D and transgenic MSP-1F. A minimum of ten schizont IFA images per parasite line (similar to those shown in Figure 2B) were analysed using the Coloc2 Plugin of ImageJ. Pearson's R values are shown plotted as mean values. Error bars, SEM. Pearson's R values close to 1.0 indicate co-localization of the MSP-1D and MSP-1F signals. The parental 3D7 and FCB1 parasites show very low Pearson's R values indicating no co-localization, while all the transgenic parasite lines have a Pearson's R value of ~0.8 indicating good co-localization of the endogenous MSP1-D and transgene-derived MSP1-F at the parasite plasma membrane, irrespective of the blasticidin concentration used for selection.

(D) Increasing blasticidin concentrations lead to increased expression of the *Renilla* luciferase gene from 3D7 parasites transfected with the control episome pHBIRH. Shown are transgene RNA transcript levels measured by qRT-PCR, as a percentile of endogenous *msp1-d* transcript levels (100%). SEM values in all cases were <0.1%.

Figure S3

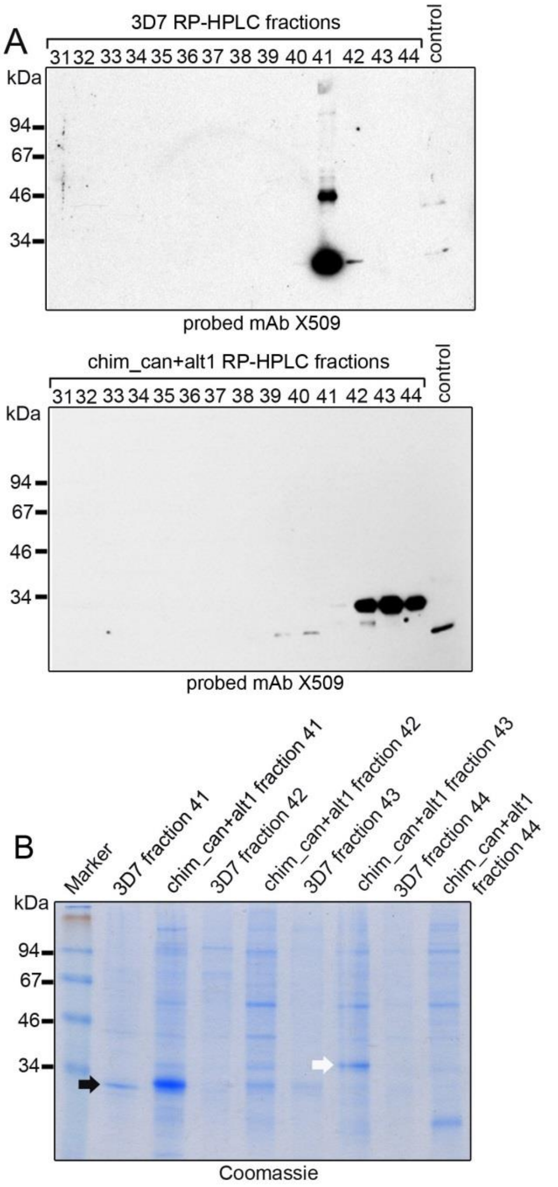

Figure S3, related to Figure 3. N-terminal Sequencing of the MSP1$_{33}$ Shedding Product from chim_can+alt1 clone B2 Parasites Confirms the 38/42alt2 Processing Site**

(A) Mature schizonts of parental 3D7 *P. falciparum* parasites or transgenic chim_can+alt1 clone B2 were cultured overnight with fresh erythrocytes in protein-free medium to allow merozoite egress and release of shed MSP1 fragments. Supernatants from the two cultures were harvested, concentrated, clarified, and separately fractionated on a C4 RP-HPLC column (Vydac) as described in *Supplemental Experimental Procedures*. Eluate fractions (1 ml) were dried, solubilised in 100 μl of

SDS sample buffer, and samples analysed by Western blot, probing with the $MSP1_{33}$-specific mAb X509. The majority of the $MSP1_{33}$ from parental 3D7 parasites was recovered in eluate fraction number 41 (upper blot). In contrast, the slightly slower-migrating modified $MSP1_{33}$** fragment purified from the chim_can+alt1 clone B2 was recovered in fractions 42 to 44 (lower blot). The lane marked 'control' in both blots is a sample of parental 3D7 culture supernatant, showing the position of migration of wild-type $MSP1_{33}$. Small amounts of $MSP1_{42}$ are also evident in the 3D7-derived material.

(B) SDS-PAGE and Coomassie blue staining of RP-HPLC fractions 41-44 from both fractionation experiments. Identity of the $MSP1_{33}$ fragment from wild type 3D7 culture supernatants (black arrow) was confirmed by Edman degradation, which identified its N-terminal sequence as AISVT. Edman degradation of the corresponding $MSP1_{33}$** shed fragment purified from supernatants of chim_can+alt1 clone B2 (white arrow) identified its N-terminus as NYDEE, confirming that it resulted from cleavage at the 38/42alt2 site.

Figure S4

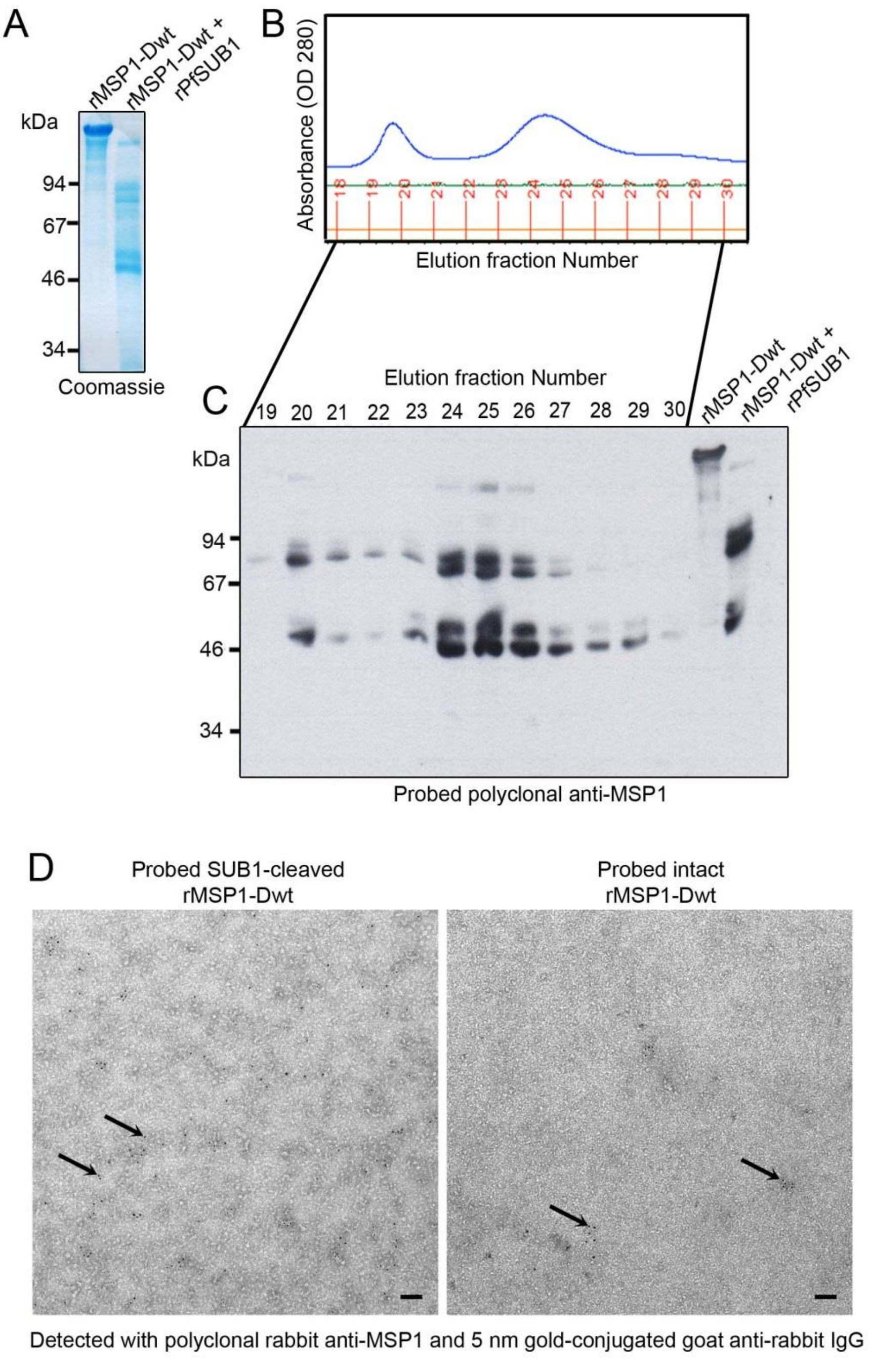

Figure S4, related to Figure 4. Proteolytic Processing *in vitro* of Recombinant MSP1-D with rPfSUB1 Produces a Non-covalently-associated Protein Complex that binds to the Erythrocyte Cytoskeleton

(A) SDS-PAGE and Coomassie blue-stained purified rMSP1-Dwt before and after *in vitro* digestion with rPfSUB1. The four dominant cleavage products are evident.

(B) Size-exclusion chromatography elution profile of rPfSUB1-digested rMSP1-Dwt (blue trace) fractionated on a Superdex 200 HR 10/30 column (GE Healthcare) equilibrated in 25 mM HEPES pH 7.4, 150 mM NaCl.

(C) Western blot analysis of the indicated elution fractions in parallel with intact protein and unfractionated total digestion products (two right-hand lanes), probing with a rabbit polyclonal anti-MSP1 antibody that detects all the processing products. Most of the cleavage products co-elute between fractions 24 to 26, indicating that they migrate predominantly as a single protein complex. The earlier eluting peak at fractions 19-20 likely represents protein aggregates. Similar results were obtained with rPfSUB1-digested rMSP1-DCD4wt (not shown).

(D) Immunolabelling of TX-100-extracted RBC cytoskeletons incubated with rPfSUB1-cleaved or intact rMSP1-Dwt. Shown are typical fields of view, with 5 nm gold beads visible as small black dots (some examples are arrowed). Scale bars, 100 nm.

Figure S5

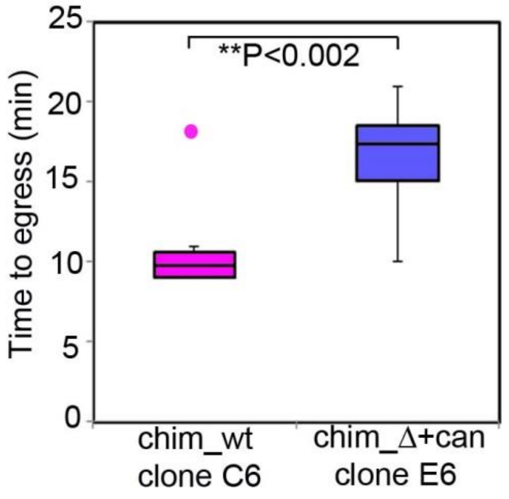

Figure S5, related to Figure 5. Egress of chim_Δ+can Parasites is Delayed relative to chim_wt Parasites

Graphical summary of egress data from Movie S2, in which egress of a 1:1 mixture of Hoechst-labelled chim_Δ+can clone E6 parasites and unlabelled chim_wt clone C6 parasites was observed by time-lapse DIC microscopy. Time to individual egress events was recorded by visual examination of movie frames. The box plot shows time to egress from the start of imaging. Horizontal lines, median. Whiskers indicate the range, with a single outlier point (>1.5x the interquartile range) indicated. The mean delay in time to egress for the chim_Δ+can schizonts in this experiment relative to the chim_wt clone was 5.7 min (P<0.002, Student's t-test), in agreement with the results presented in Figure 5B. A similar delay was observed in chim_Δ+can parasites in reciprocal experiments in which the chim_wt schizonts were labelled with Hoechst 33342 (not shown), showing that the delay in egress was not caused by the labelling. Separate analysis of DAPI-labelled segmented chim_Δ+can clone E6 and chim_wt clone C6 schizonts showed no significant differences in number of nuclei formed per schizont (mean values of 20.5±4.0 and 21.0±5.0 nuclei per schizont, respectively, n=16).

Figure S6

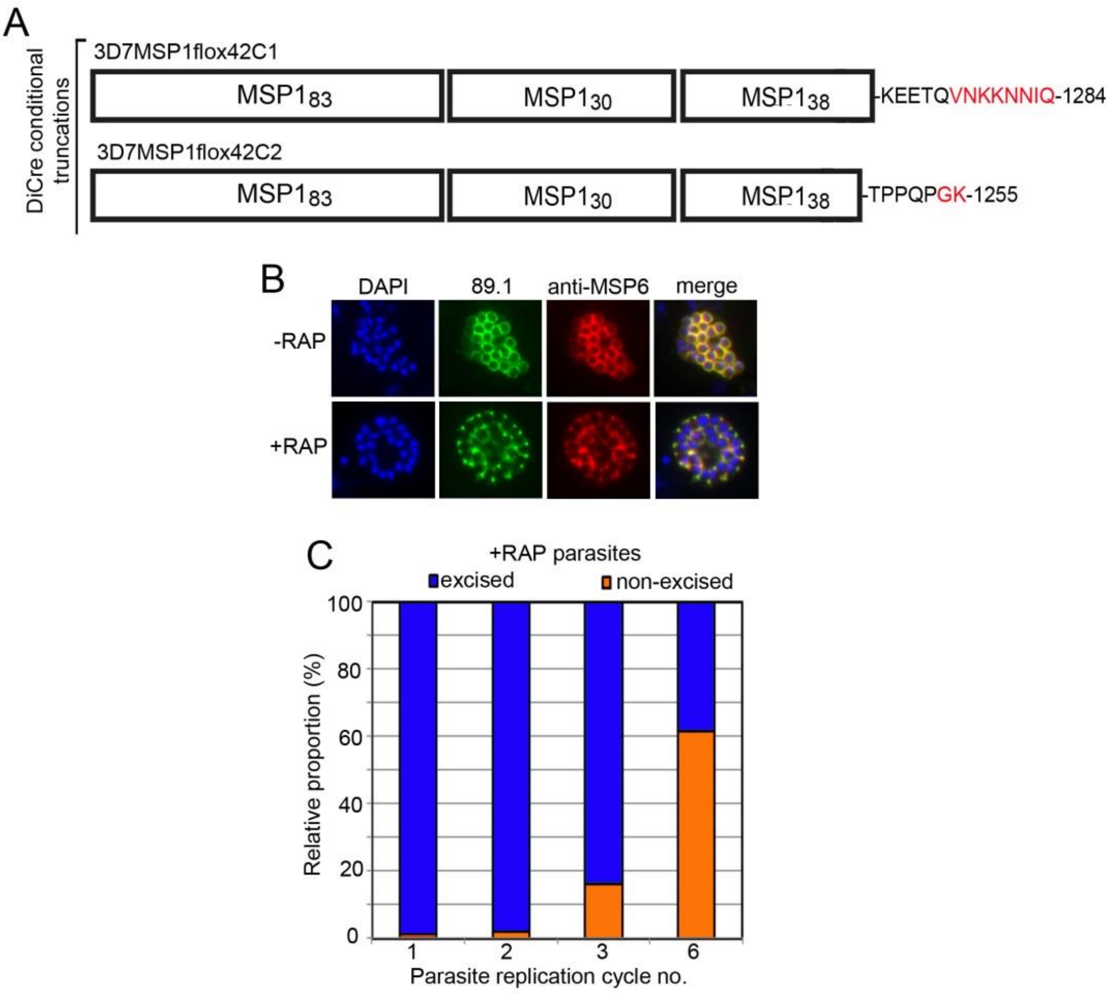

Figure S6, related to Figure 6. Truncation of MSP1 results in Loss of Merozoite-bound MSP1 complex and a Severe Fitness Defect

(A) Schematic of truncation mutants produced by DiCre-mediated conditional removal of the C-terminal segment of MSP1 and its GPI anchor. Full details of the method used to produce the two parasite lines, called 3D7MSP1flox42C1 and 3D7MSP1flox42C2 are provided in *Supplementary Experimental Procedures*. Two different strategies for truncation of the *msp1-d* gene produced slightly different C-termini (red). The predicted amino acid length of each gene product is indicated.

(B) IFA showing co-localisation of MSP1 (recognised by mAb 89.1) and the partner protein MSP6 (recognised by polyclonal anti-MSP6 antibodies) in 3D7MSP1flox42C1 clone E3 parasites. The pattern seen in the control (-RAP) parasites is typical of a normal merozoite surface localisation, whereas that observed in the RAP-treated parasites is typical of a PV 'waggonwheel' localisation.

(C) Results of a single typical experiment monitoring relative proportions of parasites expressing truncated and full-length MSP1 in a population of RAP-treated 3D7MSP1flox42C2 clone B51 parasites over the course of the ensuing 6 erythrocytic cycles (12 days). Phenotype was determined by IFA, probing thin films of the culture with both mAb 89.1 and mAb X509. The numbers of parasites expressing full-length MSP1 (reactive with both mAb 89.1 and mAb X509; non-excised) or truncated MSP1 (reactive with mAb 89.1 but not with mAb X509; excised – see Figure 6) were determined by microscopic analysis of at least 100 schizonts in each sample. Relative proportions are represented as bar graphs. The non-excised population initially formed ~1% of the population, but rapidly out-competed the excised population by the end of cycle 6 (reaching 61%), indicating that truncation of MSP1 results in a severe loss of fitness. The replication rate per cycle of excised parasites was calculated to be 2.7 fold less than that of non-excised parasites.

Supplemental Movie legends

Movie S1, related to Figure 5. chim_Δ+can Parasites Display an Egress Delay compared to chim_wt parasites

Synchronous schizonts of chim_wt clone C10 (left) and chim_Δ+can clone D2 (right) were Percoll-enriched then returned to culture and allowed to mature for 4-5 h in the presence of the PKG inhibitor compound 1 (C1, 2 μM). The parasites were washed in fresh warm medium without C1 and observed by time-lapse DIC microscopy, taking images at 5 sec intervals. Imaging commenced precisely 4 min 20 sec following C1 removal. Time after start of microscopy is indicated (top left). The mean delay in time to egress for chim_Δ+can clone D2 schizonts relative to chim_wt clone C10 in this experiment was 5.2 min ($P<0.001$, Student's t-test) (biological replicate no. 3 in Figure 5). Similar results were obtained for separate distinct clones (derived from a separate transfection) of each transgenic parasite line (chim_wt clone C6 and chim_Δ+can clone E6; not shown).

Movie S2, related to Figure 5. chim_Δ+can Parasites Display an Egress Delay compared to chim_wt parasites

Synchronous schizonts of chim_wt clone C6 and chim_Δ+can clone E6 were Percoll-enriched then returned to culture and allowed to mature for 4-5 h in the presence of C1. Just before use, the chim_Δ+can clone E6 parasites were treated for 5 min with Hoechst 33342 (1 μg mL^{-1}). The labelled parasites were then washed in warm medium containing C1 to remove excess dye prior to mixing at a 1:1 ratio with the chim_wt parasites. The schizont mixture was washed in fresh warm medium without C1 and observed by time-lapse DIC microscopy (left), taking images at 5 sec intervals. Imaging commenced exactly 5 min following C1 removal. A single fluorescence image together with a DIC image (right) was recorded just prior to starting the time-lapse imaging, enabling the chim_wt (red circles) and chim_Δ+can (blue circles) schizonts to be identified by overlaying the images. Time after start of microscopy is indicated (top left). The mean delay in time to egress for the chim_Δ+can clone E6 schizonts in this video relative to chim_wt clone C10 was 5.7 min ($P<0.002$) (Figure S5). A

similar delay was observed in chim_Δ+can parasites in reciprocal experiments in which the chim_wt schizonts were instead labelled with Hoechst 33342 (not shown), showing that the delay in egress was not caused by the labelling.

Movie S3 and S4, related to Figure 6. Dysregulation of Egress in Parasites Expressing Truncated, non merozoite-bound MSP1

Synchronised, newly-invaded forms of 3D7MSP1flox42C1 clone E3 were treated with RAP (100 nM) or vehicle only (DMSO, 1% v/v) for 4 h, then washed and cultured for a further ~44 h to allow schizont development. The schizonts were Percoll-enriched, returned to culture and allowed to mature for 4-5 h in the presence of C1, then washed in warm medium without C1 and immediately observed by time-lapse DIC microscopy, taking images at 5 sec intervals. Imaging commenced precisely 4 min 20 sec following C1 removal. Control-treated parasites (left) underwent normal 'explosive' egress with rapid dissemination of daughter merozoites. In contrast, RAP-treated parasites (right) appeared to undergo normal PVM rupture (e.g. arrowed schizont in Movie S3) but abortive RBC membrane rupture, with inefficient release of merozoites. Identical results were observed for 3DMSP1flox42C2 clone B51 (not shown).

Movie S5, related to Figure 7. *P. falciparum* Egress May Involve Shear Forces Induced by Movement of Intracellular Merozoites

Time-lapse DIC video microscopy of wild-type 3D7 *P. falciparum* showing a single egress event selected because it clearly shows that egress is a two-step process. Rupture of the PVM at ~14.1 min is followed by transient intracellular movement of the merozoites. This allows repeated impingement of the free merozoites upon the inner face of the host erythrocyte membrane before final membrane rupture and egress. Images were taken at 5 sec intervals and the movie frame rate is 10 frames/second.

Supplemental Tables

Table S1 DNA oligonucleotide primers used in this study

Primer Name	Sequence (5´-3´)
endo3D7-MSP1-BglII-targ-F	CCAACAAAGATCTGCATCCTCTACCAATACCC
endo3D7-MSP1-R	GGGTTTCTTCTTTTGTGGATCCTGAACTACCAC
syn3D7-MSP1-F	GTGGTAGTTCAGGATCCACAAAAGAAGAAACCC
syn3D7-MSP1+PstI-R	GCATGTCCTGCAGCTTGCCCTCTATGAGCTTTGATATGATGG
SDM-PIFLLSEDND-FOR	GCCAATCTTCCTCCTATCAGAAGACAACGACG
SDM-PIFLLSEDND- REV	CGTCGTTGTCTTCTGATAGGAGGAAGATTGGC
SDM-VVTLLAISVT-FOR	GTGACACTAATCGCTAGCAGGGTGACCACTTGG
SDM-VVTLLAISV-REV	CCAAGTGGTCACCCTGCTAGCGATTAGTGTCAC
SDM-FOR-NYDEE- 3rdsite	GCAGCAGGTCAAGCAACTCCTAAATTATAAGGAGGAAGACG
SDM-REV-NYDEE- 3rdsite	CGTCTTCCTCCTTATAATTTAGGAGTTGCTTGACCTGCTGC
Deletion-lowcomplexity-REV2	GACCACTTGGTCTAGAGTTACAACTGTTGTAACTTTTGCTTCTGGG
Deletion-lowcomplexity-FOR3	CTAGACCAAGTGGTCACCGGGGAAGCGATTAGTGTCAC
Deletion-lowcomplexity-VVTLL-FOR3	CTAGACCAAGTGGTCACCCTGCTAGCGATTAGTGTCAC
nearREV3	GGGTAATACAACTAAGGAATCATCTTCTTCG
Integration_UOT_FOR	GGAACATCATCTACATCCAGTCCTGG
REV2.5	GCATCAAATCCGACTCCAGTACGTCGAGG
NH1	GTGCGGCCGCGCTGTCACTCCTTCCGTG
NH2	GTGAGCTCCTATTAGATGAAGCTGTACAGGATCAG
NH3	GAGCGGCCGCATGAAAATCATTTTCTTCCTCTGTTC
NH4	GTGAGCTCCTATTAGATGAAGCTGTACAGGATCAG
f8330-mut-DG-For	CATTAAAACTGAAGACCAGTCAGGTAACTCCGAGCC
f8330-mut-DG-Rev	GGCTCGGAGTTACCTGACTGGTCTTCAGTTTTAATG
f3038-KK-For	GACAAGCCCGAAAAGAGCAAGAACGACGACACC
f3038-KK-Rev	GGTGTCGTCGTTCTTGCTCTTTTCGGGCTTGTC
f3842can-mutDD-For	CTCGGCCAGGTGGACACCGATGAGGCTGTCACTCC
f3842can-mutDD-Rev	GGAGTGACAGCCTCATCGGTGTCCACCTGGCCGAG
f3842can-mutKK-For	CTCGGCCAGGTGAAGACCAAGGAGGCTGTCACTCC
f3842can-mutKK-Rev	GGAGTGACAGCCTCCTTGGTCTTCACCTGGCCGAG
f3842alt-mutKK-For	CATCATTGTGCCTAAGTTCAAGGAGAGCGAGGAG
f3842alt-mutKK-Rev	CTCCTCGCTCTCCTTGAACTTAGGCACAATGATG
f3842-2ndalt-mutKK-For	CCTGCATCTACTCATGTCAAAAAAGAGTCCAATAC
f3842-2ndalt-mutKK-Rev	GTATTGGACTCTTTTTTGACATGAGTAGATGCAGG
CB-mutLL-P2P4-For	GTCCAATACAATTCTCACATTGCAGAACGTCGACGATG
CB-mutLL-P2P4-Rev	CATCGTCGACGTTCTGCAATGTGAGAATTGTATTGGAC
bsd-3-up	TGGCAACCTGACTTGTATCG
bsd-3-down	AGCAATTCACGAATCCCAAC
f83-up	AACCAATCCATCTGACAAC
f83-down	ATTTGAAGCCATGAATGTT

Table S2 Primary antibodies used in this study

mAb X509 (human)	Monoclonal antibody recognising an epitope within the N-terminal 100 amino acid residues of the *P. falciparum* 3D7-type $MSP1_{42}$. The antibody therefore recognises both $MSP1_{42}$ and $MSP1_{33}$ (Blackman et al., 1991).
mAb 89.1 (mouse)	Monoclonal antibody recognising an epitope within the repeat regions in 3D7-type *P. falciparum* $MSP1_{83}$ (Holder and Freeman, 1982).
mAb 111.4 (mouse)	Monoclonal antibody recognising an epitope within the C-terminal 100 amino acid residues of the FCB1-type $MSP1_{42}$ (Holder et al., 1985).
Polyclonal anti-AMA1 (mouse)	Polyclonal antiserum raised against recombinant *P. falciparum* 3D7 AMA1 (Collins et al., 2009)
Polyclonal anti-MSP1 (rabbit)	Rabbit polyclonal antiserum raised against parasite-derived full-length *P. falciparum* MSP1 (T9/94 clone) (M. Blackman, unpublished)
mAb OX68 (mouse)	Monoclonal antibody against rat CD4 (domains 3 and 4). (Santa Cruz Biotech)
Polyclonal anti-PfSUB1 (rabbit)	Rabbit antiserum raised against a recombinant fusion protein extending from residues Ser^{330} to His^{690} of PfSUB1 (Blackman et al., 1998).
Polyclonal anti-$MSP1_{33}$, MSP1-F specific antiserum (rabbit)	Rabbit antiserum raised against a recombinant protein corresponding to a 93 amino acid residue region of the $MSP1_{33}$ fragment of the FCB1-type *P. falciparum* MSP1 (Blackman et al., 1993)
Polyclonal MSP1-D specific antiserum (rabbit)	Anti-3D7-MSP-$1D_{42\Delta EGF}$ rabbit serum. Kind gift of H. Bujard, ZMBH, Heidelberg, GER
Polyclonal anti-$MSP1_{38}$, MSP-1D specific (rabbit)	Kind gift of Prof. H. Bujard, ZMBH, Heidelberg, Germany
Polyclonal anti-MSP6 (rabbit)	Kind gift of Prof. H. Bujard, ZMBH, Heidelberg, Germany

Supplemental Experimental Procedures

Peptide cleavage assays

N-terminally acetylated synthetic decapeptides were procured from Biomatik at a purity of at least 95%. Peptides were dissolved in DMSO and concentrated stock solutions (100 mM) stored at -20°C. For digestion assays, peptide stocks were diluted to 5 mM in a total volume of 100 µL in SUB1 digestion buffer (25 mM HEPES pH 7.4, 12 mM $CaCl_2$, 25 mM CHAPS). This was divided into two tubes, each containing 50 µL diluted peptide. To one tube, 5 µL recombinant PfSUB1 (rPfSUB1) (specific activity 1500 U/mL) was added. Both tubes were incubated at 37°C for 2 h. Cleavage of peptides was then assessed using gradient elution reversed phase high pressure liquid chromatography (RP-HPLC). 10 µL samples of digested or undigested peptides were fractionated on a 4.6 mm x 25 cm C18 RP column (Vydac) eluting at 1 mL/min with a 0-45% (v/v) gradient of acetonitrile in 0.1% trifluoroacetic acid (TFA) over 35 min. Digestion products were identified by electrospray mass spectrometry as previously described (Blackman et al., 2002; Withers-Martinez et al., 2002). Peptide products of digestion were identified as Ac-PIFGE (predicted *m/z* 604.298, observed 604.303), SEEDY (predicted *m/z* 642.225, observed 642.232), NYDEE (predicted *m/z* 628.367, observed 628.4) and Ac-VVQLQ (predicted *m/z* 669.236, observed 669.2).

Recombinant expression in *Escherichia coli* of wild-type and mutant MSP1-F proteins

Design of plasmid expression constructs. Expression constructs for full length MSP1-F (Fwt) or heterodimeric MSP1-F (Fwt heterodimer) were engineered using pZ vectors as the backbone and expressed as described previously (Kauth et al., 2003). Mutations were introduced into the synthetic *msp1-f* gene by QuikChange II site-directed mutagenesis (SDM). The construct to produce mutant Fmut83/30 (Figure S1) was generated by introducing mutations to create pZ-Fmut83/30 by using primers f8330-mut-DG-For and f8330-mut-DG-Rev. Construct pZ-Fmut30/38 was similarly created

using primer pair f3038-KK-For and f3038-KK-Rev, and pZ-Fmut38/42canD was created using primer pair f3842can-mutDD-For and f3842can-mutDD-Rev. Mutations at the canonical 38/42 site were introduced to create pZ-Fmut38/42canK using primers f3842can-mutKK-For and f3842can-mutKK-Rev. This plasmid was then modified to pZ-Fmut38/42can+alt1K by performing SDM PCR using primers f3842alt-mutKK-For and f3842alt-mutKK-Rev. The plasmid was further modified to create pZ-Fmut38/42can+alt1+2K using primers f3842-2ndalt-mutKK-For and f3842-2ndalt-mutKK-Rev. To introduce mutations at all 38/42 sites, construct pZ-Fmut38/42triple was created by SDM of pZ-FCB1mut38/42can+alt1+2K using primers CB-mutLL-P2P4-For and CB-mutLL-P2P4-Rev.

Recombinant protein purification and processing assays. Recombinant protein aggregated in inclusion bodies and was refolded by pulse renaturation according to (Rudolph and Lilie, 1996). The two separately produced halves of the Fwt heterodimeric protein and corresponding heterodimeric mutants called Fmut83/30 and Fmut38/42 were re-associated during refolding by addition in equal amounts (0.1 mg/mL) at each step. Purified Fwt, Fwt heterodimer and modified versions were digested *in vitro* by rPfSUB1 as described previously (Koussis et al., 2009) with some modifications to the protocol. The protein concentration was adjusted to 0.5 mg/mL with SUB1 digestion buffer along with addition of protease inhibitors E64 (10 µM), leupeptin (10 µg/mL) and Pefabloc (100 µg/mL). 1.5 units of rPfSUB1 was added per 100 µL of volume and the reaction incubated at 16°C. For time-course digests, seven reactions were set up and the reaction was stopped after 0, 30 min, 1 h, 3 h, 6 h and overnight by boiling in SDS sample buffer for 10 min at 80°C. The 16°C incubation temperature was used in order to reduce protein precipitation during the long incubation periods.

Transgenic expression of MSP1-F in 3D7 parasites

Creation of plasmid transfection constructs. The R*enilla luciferase* gene from expression plasmid pHBIRH (Epp et al., 2008) was replaced by the synthetic sequence encoding the entire *msp1-f* gene from *P. falciparum* FCB-1 including sequences for the signal peptide and GPI anchor signal (Pan et al.,

1999) to yield pHBIMHFwt after several cloning steps. First, the *Renilla luciferase* gene of pHBIRH was replaced via restriction sites NotI (5´) and SacI (3´) by a region encoding MSP1-$F_{42,}$ which was previously amplified via PCR using primers NH1 and NH2, resulting in plasmid pHBI-f42. Synthetic full-length *msp1-f* (Pan et al., 1999) was PCR amplified using primers NH3 and NH4 and inserted into pHBI-f42 using NotI and BstBI restriction sites. The *msp1-f* sequence in the resulting expression vector pHBIMHFwt was verified by nucleotide sequencing on both strands. Mutations Fmut38/42triple and Fmutall were further introduced by SDM into pHBIMFwt to create vectors pHBIMFmut38/42 and pHBIMFmutall respectively, which were also verified by nucleotide sequencing on both strands.

Transfection into P. falciparum 3D7 parasites. The created constructs were transfected into *P. falciparum* 3D7 parasites by DNA loading of red blood cells (RBC). 2 mL of RBC (50% haematocrit) were mixed with 6 mL incomplete cytomix and centrifuged for 2 min at 800 g. The supernatant was removed and the blood cell pellet was washed once. 400 μL of the RBC pellet was mixed with 400 μL incomplete cytomix and 100 μg of plasmid DNA. The mixture was transferred to two pre-chilled cuvettes and incubated on ice. Electroporation was performed at 0.31 KV and 960 μFD capacitance. The time constant was in the range of 10 to 13 ms. The cuvette was put back on ice for 5 min and the cells then transferred to a 15 mL tube containing 4 mL RPMI-Albumax medium. The cells were pelleted by centrifugation at 800 g for 2 min and supernatant containing lysed red blood cells was discarded. The transfected erythrocytes were then mixed with 100 μL schizont-infected red blood cells (1% parasitemia) to achieve a final parasitemia of 0.2–0.3%. Culture medium (10 mL) was added and the petri dish was returned to the incubator, with daily changes of culture medium. Selection with blasticidin was performed initially at 2 μg/mL concentration, which was subsequently increased when blasticidin resistant parasites reached a parasitemia of at least 1%.

Growth rate determination. Parasitaemia measurements were carried out using FACSCalibur (Becton-Dickinson). As negative controls, stained uninfected erythrocytes as well as unstained infected erythrocytes were used. For determination of parasitaemia, 10^5 erythrocytes per sample were counted. Results were displayed in a histogram plotting the fluorescence FL3 (x-axis) against the total cell count (y-axis). This representation allowed the discrimination of different parasite stages by their DNA content, which was proportional to fluorescence intensities. For the determination of relative parasite replication rates, expansion of parasitemia over one life cycle was measured. Mature schizonts were set to 0.5% parasitemia and measured by flow cytometry. After one growth cycle (approximately 44 h) the parasitemia was measured again. The growth factor was calculated by dividing the parasitemia at 44 h by the starting parasitemia at 0 h. Significance was determined using Kruskal-Wallis test.

Plasmid rescue. Parasite lines maintained in the presence of a range of blasticidin concentrations, were used for preparation of genomic DNA and episomes rescued by transformation of *E. coli*. Randomly-selected individual bacterial colonies were expanded in LB, plasmid DNA extracted and digested with BglII and XhoI. Digestion products were analysed by agarose gel electrophoresis to visualise diagnostic bands.

Quantitative Real time PCR (qRT-PCR). First strand synthesis of cDNA was performed using the SuperScript® II First-Strand Synthesis Kit (Invitrogen) according to the manufacturer's instructions. Briefly, reverse transcription of 0.5 µg RNA was carried out in a thermal cycler by incubating the reaction for 10 min at 22°C followed by 50 min at 42°C and terminating the reaction by heating to 70°C for 15 min. The reverse transcription product was used directly in PCR reactions or stored at -20°C. qRT-PCR was performed with gDNA and cDNA preparations using the ABI 7500 sequence detection system and SensiFAST™SYBR® Lo-ROX Kit (Bioline) according to the manufacturer's

instructions. The concentration of gDNA templates was adjusted to 30 ng/µL while cDNA was diluted 1:5 prior to use as template. qRT-PCR was performed in triplicate. Data were analysed with SDS 1.3.1 software (Applied Biosystems). In order to determine the absolute copy number of genes or transcripts number, standard curves were generated. The relative gene copy or transcript number was determined by normalisation with the respective data for actin (p100). Transgene expression was displayed as a percentile of *msp1-d* expression (100%). Each gene used for absolute quantification was amplified using serial dilutions from either genomic DNA (*msp-1d*, *actin*) or from plasmid DNA (*msp-1d*, *msp-1f*, *bsd, Renilla luciferase*). The DNA concentration was determined by spectrophotometry and adjusted to 10^8 copies per 20 µL solution. Serial 10-fold dilutions ranging from 10^7 to 10^{-1} copies per real-time PCR reaction were made in H_2O. Triplicate measurements were made for each dilution. The obtained Ct values were plotted against the copy number and a best-fit standard curve was generated. The standard curves were linear across a range of seven logs of DNA concentrations. The detection limit varied between the analysed genes but usually was 10 copies. Standard curve equations were used to calculate the copy numbers obtained by analysing the transgenic parasite lines.

Modification by homologous recombination of the *P. falciparum* 3D7 *msp1* gene

Design of targeting constructs. Construct pHH1-3D7wt was designed to integrate by single-crossover homologous recombination into the 3D7 *msp1-d* locus, reconstituting the coding sequence of the endogenous gene with a gene that expresses a chimeric MSP1 protein. This encodes the 3D7 type MSP1 sequence except for the C-terminal 19 kDa region which is of the FCB1/Wellcome type ($wMSP1_{19}$). To reliably introduce mutations at the 38/42 position, the DNA sequence in the entire C-terminal region (except that encoding the 19 kDa fragment), from the SUB2 cleavage site near the 3′ end to 168 bp upstream of the 38/42 canonical cleavage site at the 5′ end was recodonized in the resultant plasmid. Upstream of the recodonized sequence was 998 bp of endogenous 3D7 sequence (target sequence) to drive single cross-over homologous recombination at the *msp1-d* locus. The 998

bp targeting fragment was created by amplifying from *P. falciparum* 3D7 genomic DNA using primers endo3D7-MSP1-BglII-targ-F and endo3D7-MSP1-R. The recodonized fragment was created by amplifying recodonized sequence from plasmid pZ-3D7-MSP138/42 (Kauth et al., 2003; Pan et al., 1999) using primers syn3D7-MSP1-F and syn3D7-MSP1+PstI-R. The fragments were then joined together by overlapping PCR (using primers endo3D7-MSP1-BglII-targ-F and syn3D7-MSP1+PstI-R) to create a fragment with 5´ BglII and 3´ PstI restriction site overhangs. This was ligated into the pHH1-$MSP1_{19}$ backbone, which has the $wMSP1_{19}$ sequence at the 3´ end (created by digesting plasmid pMSP1chimWT (Child et al., 2010) with BglII and PstI). The resultant plasmid construct was called pHH1-3D7wt, which was used for transfection into 3D7 parasites, or further modified to create mutations in the 38/42 cleavage region.

For creation of the mutant constructs, pHH1-3D7wt was digested with BglII and PstI and the insert sub-cloned into the pSL1180 vector. The coding sequence flanking the 38/42alt1 site was then mutagenized using the Quikchange® site-directed mutagenesis kit (Agilent) by SDM from PIFGESEDND to PIFLLSEDND using primers SDM_PIFLLSEDND_FOR and SDM_PIFLLSEDND_REV, to create construct pHH1-3D7mut38/42alt1. The 38/42 canonical site was similarly mutagenized from VVTGESEDND to VVTLLSEDND with primers SDM-VVTLLSEDND-FOR and SDM-VVTLLSEDND-REV, to create construct pHH1-3D7mut38/42can. The double mutant construct pHH1-3D7mut38/42double was created by introducing the PIFLLSEDND mutation in the plasmid pHH1-3D7mut38/42can. The alt2 site mutant construct pHH1-3D7mut38/42alt2 and the triple mutant construct pHH1-3D7mut38/42triple were similarly created by introducing the VVQLQNYDEE→VKQLLNYKEE mutation in the plasmids pHH1-3D7wt and pHH1-3D7mut38/42double respectively, using primers SDM-FOR-NYDEE-3rdsite and SDM-REV-NYDEE-3rdsite. All final construct sequences were verified by nucleotide sequencing on both strands.

All three putative MSP1 38/42 cleavage sites are contained within a predicted unstructured region (not shown) in the 38/42 cleavage region of MSP1. It was reasoned that this region could be deleted so long as at least a single 38/42 cleavage site (the canonical 38/42 site VVTGE↓AISVT) was

left intact. In order to delete this region, plasmids pHH1-3D7mut38/42Δ+can (which encodes an MSP1 protein product with 69 amino acids deleted but retains the downstream canonical 38/42 site unmodified) and pHH1-3D7mut38/42Δ+mut (which combines the 69 amino acid deletion with the canonical site mutation VVTGE↓AISVT → VVTLLAISVT) were both designed to integrate into the *msp1-d* locus by single cross over homologous recombination. Using construct pHH1-MSP1wt as template, a targeting region for homologous recombination (fragment A) was amplified by PCR using primers endo-3D7-MSP1-BglII-targ-F and Deletion-lowcomplexity-REV2. In order to ensure integration upstream of the deletion to be introduced, the deletion was engineered into the 3D7 recodonized sequence. The recodonized fragment containing the deletion, "B_{VVTGE}", was created by PCR using primers Deletion-lowcomplexity-FOR3 and syn-3D7-MSP1+PstI-R. Another recodonized fragment containing additional point mutations to render the canonical site uncleavable by PfSUB1 (VVTGE↓AISVT → VVTLLAISVT), called "B_{VVTLL}" was generated using primers Deletion-lowcomplexity-VVTLL-FOR3 and syn-3D7-MSP1+PstI-R. Fragments A and B_{VVTGE} (or B_{VVTLL}) were stitched together by a second step of overlapping PCR using the outside primers endo-3D7-MSP1 BglII-targ-F and syn-3D7-MSP1+PstIR. The resulting A+B_{VVTGE} and A+B_{VVTLL} fragments contained a 207 bp deletion encoding the following 69 amino acid residues (alt1 and alt2 SUB1 cleavage sites are shown in bold):

PPQPDVTPSPLSVRVSGSSGSTKEETQIPTSGSLLTELQQ**VVQLQNYDEE**DDSLVVL**PIFGESEDND**EY

The 5' and 3' extremities of the two stitched fragments were digested with restriction enzymes BglII and PstI and ligated into the parental plasmid pHH1-MSP1wt, thus creating plasmid constructs pHH1-3D7mut38/42Δ+can and pHH1-3D7mut38/42Δ+mut. The presence of the deletion and mutations was confirmed by nucleotide sequencing on both strands.

Transfection of 3D7 P. falciparum for homologous integration into the msp1 locus. Approximately 10 μg of plasmid DNA was ethanol precipitated and resuspended in 10 μL sterile buffer TE (Qiagen). The Amaxa™ P3 primary cell 4D Nucleofector™ X Kit L (Lonza) was used for transfections. The input DNA was added to 100 μL P3 primary cell solution, mixed with 10-20 μL of packed synchronous mature

schizonts and added to the cuvette, which was electroporated in a 4D-Nucleofector machine (Lonza) using program FP158. The transfected schizonts were rapidly added to 2 mL of complete medium (RPMI Albumax supplemented with glutamine) containing erythrocytes at a haematocrit of 15%, and transferred to a shaking incubator at 37°C for 30 min. Finally the cultures were supplemented with 7 mL of complete RPMI medium to obtain a final haematocrit of 3% and incubated overnight at 37°C in a small angle-necked flask (Nunc™). The presence of the human dihydrofolate reductase (*hdhfr*) selectable marker in the transfection plasmids allowed selection of integrants with the antifolate WR99210 (Jacobus Pharmaceuticals, New Jersey, USA), added to 2.5 nM 20 h after transfection. The culture medium was subsequently exchanged every day for the next 4 days to remove cell debris which accumulates during electroporation and then twice a week until parasites were detected by Giemsa smear. Drug-resistant parasites were generally detectable in thin blood films 2-3 weeks post transfection. After this, parasite stocks (at ~5% ring parasitaemia) were cryopreserved in liquid nitrogen and genomic DNA was prepared for parasites containing integration vectors. Integrants were selected by drug cycling as follows. Drug was removed from the medium and parasites cultured in its absence for 3-4 weeks, after which the drug was added back and the medium changed daily for 2 days. Once parasitaemia was re-established, parasites were cryopreserved in liquid nitrogen and genomic DNA was prepared. The above cycling process was repeated until integration was detectable by PCR analysis. Integration was confirmed by diagnostic PCR. Integrant lines were then cloned by limiting dilution. For this, the parasite culture was diluted in medium containing fresh red cells at a haematocrit of 2% in order to dispense 0.3 parasite per 100 μL per well in a 96-well microplate (Nunc™). The medium was changed every two or three days until growth was detectable by eye due to a change in the colour of the medium (about two weeks after cloning). Clones were expanded and cryopreserved.

Diagnostic PCR to detect integration of targeting constructs. To assess whether transfection constructs had integrated into the *msp1-d* genomic locus, diagnostic PCR was performed using extracted genomic DNA as template. Primer pairs specific for detection of integration, namely Integration-UOT-FOR and REV3 (or REV2.5 for deletion constructs) were designed such that the forward primer hybridised in a genomic region upstream of the targeting region present in the plasmid and the second primer hybridised in a region unique to the introduced plasmid. Primer pairs Integration-UOT-FOR and nearREV3 were designed to detect presence of the unmodified *msp1-d* locus.

Purification and N-terminal sequencing of the shed MSP1$_{33}$ fragment

Culture supernatants (~50 ml) collected in Albumax-free medum from wild-type 3D7 or transgenic parasite schizonts allowed to undergo egress for 5 h were clarified, then concentrated to 1 mL using 10 kDa cut off ultrafiltration Centricon filters (Millipore). The concentrated culture supernatants were acidified by the addition of 20 μL TFA and loaded onto a Vydac 4.6 mm x 150 mm 214TP C4 RP-HPLC column. Bound proteins were eluted with an acetonitrile gradient, eluting at 1 mL/min with a 0-40% (v/v) gradient of acetonitrile in 0.1% (v/v) TFA over 20 min, followed by 40-55% (v/v) gradient of acetonitrile in 0.1% (v/v) TFA over 30 min. Collected eluate fractions (1 mL each) were dried overnight in a Speedvac. Dried fractions were each resuspended in 100 μL of 1x SDS reducing sample buffer. Small samples of each of these fractions were electrophoresed on 12.5% SDS PAGE gels and analysed by Western blot, probing with mAb X509 to determine the position of elution of the MSP1$_{33}$ fragment or the equivalent fragments from the mutant clones. Proteins of interest were then subjected to N-terminal sequencing. For this, remaining samples of fraction of interest were transferred from a 8-16% precast gradient gel (Invitrogen) onto a PVDF membrane (Immobilon P, Millipore) by overnight transfer (150 mA current) using CAPS buffer (10 mM CAPS, 5 mM DTT, 10% methanol, pH 11). Transferred proteins were visualised with Coomassie brilliant blue. N-terminal

sequencing analysis was carried out by the Protein and Nucleic Acid Chemistry Facility (University of Cambridge, UK).

Purification of recombinant full length recombinant MSP1-D

Recombinant full length MSP1-D proteins (rMSP1-Dwt, rMSP1-DCD4wt and rMSP1-DCD4mut) with an N-terminal secretory signal and a C-terminal hexahistidine (His_6) tag was expressed in HEK293E cells and secreted into culture supernatant as described previously (Crosnier et al., 2013). For purification, ~1 litre of culture supernatant was buffered by the addition of 1 M Tris-HCl, pH 8.2 to 20 mM, then incubated with Ni-NTA agarose beads (15 mL of slurry), and washed with equilibration buffer (20 mM Tris-HCl pH 8.2, 300 mM NaCl). Unbound material was removed by passing the suspension through a sintered glass funnel and the beads were washed with 200 mL of equilibration buffer. The Ni-NTA agarose bound rPfMSP1 was eluted into 45 mL of elution buffer (250 mM imidazole in 20 mM Tris-HCl , 300 mM NaCl). The eluted material was concentrated to 250 μL using a Centricon™ -70 Plus (30 kDa cut off) tube then further purified by size exclusion chromatography using a HiLoad Superdex®200 26/60 column equilibrated in 25 mM HEPES pH 7.4, 150 mM NaCl, collecting 5 mL fractions. Purity and yield was assessed by subjecting 20-30 µL samples of the eluted fractions to SDS PAGE and staining with Coomassie brilliant blue. Purified proteins were stored at -80°C until required for further experiments.

Circular dichroism studies on recombinant MSP1

Full length rMSP1-Dwt or mutant forms at 0.156 mg/mL concentration in 500 μL buffer (25 mM HEPES, pH 7.4, 150 mM NaCl, 15 mM CaCl2) was equilibrated at 37°C and monitored for 5 h for any structural changes on a Jasco J-715 spectropolarimeter. To examine the effects of digestion with PfSUB1, 6 μL of rPfSUB1 (0.84 mg/mL in the sample) was added to another identical sample and monitored for structural changes under identical conditions. The secondary structure composition was averaged using the algorithms CONTINLL and SELCON3 (Sreerama and Woody, 2004).

Heparin binding assays

Heparin-agarose beads (Sigma Aldrich) suspended in a 50% slurry in 20% ethanol were washed twice with distilled water and then three times with heparin assay buffer (25 mM HEPES, pH 7.4, 15 mM NaCl, 0.07% Tween® 20). The beads were resuspended in an equal volume of heparin assay buffer and 50 μL of the resuspended slurry dispensed into 1.4 mL Eppendorf tubes for the binding experiment. 50 μL of purified rMSP1-Dwt (cleaved with rPfSUB1 or uncleaved) was added to a final concentration of 0.1 μg/μL to the heparin beads. Control tubes were set up where binding was tested in the presence of 1 mg/mL heparin sodium salt solution (Sigma). The beads were incubated with proteins of interest for 20 min with shaking at room temperature. Unbound supernatant was collected and the required amount of SDS sample buffer (+DTT) was added. The beads were washed 5 times with 500 μL heparin assay buffer and bound proteins finally eluted into SDS sample buffer (containing DTT). All samples were heated for 5 min at 90°C then electrophoresed on a 4-16 % BioRad® MiniProtean™ TGX gradient gel. The gel was Coomassie stained and imaged on the BioRad® Chemidoc™ MP system. Total protein intensity in each lane was estimated using Image Lab software.

Overlay immunofluorescence for investigating MSP1 interactions with erythrocytes

Erythrocytes were washed, resuspended in RPMI-Albumax medium and smeared on a glass slide. The thin films were air dried, fixed in 4% paraformaldehyde (in PBS) for 30 min at RT and permeabilised in 0.1% (v/v) Triton X-100 (Sigma) for 10 min. Fixed slides were then washed three times with PBS for 10 min and blocked overnight at 4°C in 3% (w/v) bovine serum albumin (BSA) in PBS. The following day, slides were probed with purified rMSP1-Dwt (previously cleaved with rPfSUB1 or uncleaved) at 25 ng/μL in 1% BSA in PBS 0.005% (v/v) Tween 20 (PBST). The slides were then washed three times with PBS for 10 min. Next, the slides were probed with an anti-MSP1 primary antibody (purified mAb X509 at a dilution of 1:2000) for 30 min at 37°C and then washed three times for 10 min in PBS. Slides were then probed with Alexafluor conjugated goat anti-human

IgG (1:1000) and washed three times with PBS. Slides were mounted in PBS/Glycerol and images collected using AxioVision 3.1 software on an Axioplan 2 Imaging system (Zeiss) using a Plan-APOCHROMAT 100x/1.4 oil immersion objective (Harris et al., 2005). Mean pixel intensity was determined for the fluorescence images using the Histogram tool in Adobe Photoshop.

Overlay (Far Western) assay for investigating spectrin binding by MSP1

Erythrocyte ghosts were prepared and overlay assays performed as described previously (O'Donnell et al., 2006). Briefly, human RBC were washed in RPMI medium (without Albumax) and lysed in 5 mM ice cold sodium phosphate buffer, pH 7 (diluted from a 1 M stock prepared by mixing 57.7 mL of 1 M Na_2HPO_4 and 42.3 mL of 1 M NaH_2PO_4 solutions). Ghosts were centrifuged (13,000 rpm in a Heraeus Biofuge Fresco) and washed repeatedly until the supernatant appeared clear and the erythrocytes ghosts appeared a pale white colour. Ghosts were stored at - 80°C until further use. The ghosts were thawed, solubilized in SDS-sample buffer and heated for 4-5 min at 95°C. Ghost preparations were run on a 4-16% gradient gel and transferred to a nitrocellulose membrane by overnight Western transfer. The blot was then blocked in 5% milk in PBST for 1 h. The blot was washed 3 x 5 min with PBST and probed with rMSP1-Dwt, rMSP1-DCD4wt, or rMSP1-DCD4mut protein (previously digested with rPfSUB1 or undigested). For this a 6 mg/mL solution of purified protein in 25 mM HEPES pH 7.4, 150 mM NaCl was diluted 1:400 into 2 mL of PBST containing 1% (w/v) BSA and incubated with the blot for 2 h. The blot was then washed 3 x 5 min in PBST before probing with a primary anti-MSP1 antibody (purified mAb X509 diluted 1:2000 in PBST 1% BSA, or mAb OX68 diluted 1:1000 in PBST 1% BSA) for 1 h. MAb OX68 was used if the recombinant MSP1 possessed a C-terminal CD4 tag. This was followed by washing and 1 h incubation in an appropriate HRP-conjugated secondary antibody (goat anti human IgG for mAb X509, goat anti mouse IgG for mAb OX68). Both secondary antibodies were used at 1:10,000 dilution in PBST 1% BSA. The blot was developed using ECL reagents and either visualised using photographic films or using a Biorad Chemidoc™ MP system.

Binding of rMSP1 to inside-out vesicles (IOVs)

Preparation of IOV. IOVs were prepared from human RBC as described previously (Kilili and LaCount, 2011). Briefly, RBC from 40 ml of whole blood were collected by centrifugation and washed at least three times in five volumes of cold RPMI medium without Albumax, calcium or magnesium. After each wash, cells were pelleted and the supernatant, along with any visible buffy coat, was removed. After the final wash, RBCs were resuspended in RPMI without Albumax at 50% hematocrit and stored at 4°C. RBC ghosts were prepared from 1 ml of packed RBCs by adding 30 ml of ice-cold lysis buffer (5 mM phosphate buffer, pH 8.0, protease inhibitor (PI) cocktail, and 0.1 mM EDTA), mixing several times by inversion, and incubating on ice for 10 min. Lysed cells were centrifuged at 48,000 x g for 10 min. After the removal of approximately 95% of the supernatant, the pellet was dislodged by slowly swirling in the remaining buffer and transferred to a fresh tube, leaving behind the contaminating intact RBC pellet. Fresh buffer was added, and the centrifugation and pellet transfer steps were repeated two to three times until the pellet appeared creamy white and the supernatant was free of haemoglobin.

To prepare IOVs, the pellet was resuspended in 30 ml of vesiculation buffer (0.5 mM phosphate buffer, pH 8.5), incubated on ice for 1 h, and centrifuged at 48,000 x g for 15 min. The pellet was resuspended in 1 ml of vesiculation buffer, and the suspension was passed through a 27-gauge needle at least 10 times. Vesiculation buffer was added to adjust the volume to 30 ml, and the suspension was subjected to centrifugation as described above. The pellet, which contained the IOVs, was resuspended in 1.5 to 2 ml of IOV storage buffer (138 mM NaCl, 5 mM KCl, 6.1 mM Na_2HPO_4, 1.4 mM NaH_2PO_4, 5 mM glucose) and stored at 4°C for less than 1 week until use.

IOV pulldown assays. IOVs (specific amounts used are indicated in the figure legends) were incubated overnight without mixing at 4°C in 250 μl of cold IOV blocking buffer (1x PBS, 2% w/v BSA, PI cocktail). The IOVs were collected by centrifugation at 16,000 x g for 10 min at 4°C, and the

supernatant was removed by aspiration. The pellet was resuspended in 250 μl of freshly made ice-cold IOV binding buffer (1x PBS, 1% BSA, PI cocktail). Equal amounts of rPFSUB1-cleaved or uncleaved rMSP1-Dwt (see figure legends for details) were added to the IOVs, mixed briefly, and incubated on ice overnight at 4°C with occasional mixing during the first 2 to 3 h. The IOVs then were washed twice with cold IOV wash buffer (1x PBS, 0.25 mM KCl) by completely suspending the pellet and incubating it for 5 min on ice. After the last wash, the pellet was resuspended in 3x SDS sample loading buffer and processed for SDS-PAGE followed by Western blotting probing with the MSP1-specific mAb 89.1.

Immuno-electron microscopic analysis of binding of recombinant MSP1 to RBC cytoskeletons

Erythrocyte cytoskeletons were immobilised on carbon-coated gold grids and binding by recombinant MSP1 was assessed as follows. Cytoskeletons of uninfected erythrocytes were prepared *in situ* on grids as follows. Grids were glow-discharged, coated with 0.01% (w/v) poly-*L*-lysine (Sigma) for 30 seconds, washed once with water and blotted dry. Erythrocytes in PBS (~20% haematocrit) were applied to the grids, allowed to adhere for 1 minute, then blotted from the back and washed once with PBS. Grids carrying cells were dipped sequentially into low salt lysis buffer (1 mM Tris pH 4.7, 1 mM KCl, 0.2 mM $MgCl_2$), low salt lysis buffer containing 2% Triton X-100, and then low salt lysis buffer without detergent to wash, for 60 seconds in each solution, passing through the meniscus several times. The resulting cytoskeletons were then incubated for 30 minutes in blocking buffer (0.5% w/v cold water fish skin gelatine, 1% normal goat serum), followed by incubation for 30 min with rMSP1-Dwt (rPfSUB1-cleaved or uncleaved) in blocking buffer. The grids were then probed with a rabbit polyclonal anti-MSP1 antiserum diluted 1:250 or 1:2500 in blocking buffer, followed by probing with gold-conjugated secondary antibody (goat anti-rabbit Alexa488 conjugated to 5 nm gold) diluted 1:10 in blocking buffer. Grids were washed with PBS between incubation steps, and finally washed with lysis buffer and stained with 2% sodium silicotungstate. Controls included no-protein controls and no primary antibody controls. At least 8-10 separate RBC ghost skeletons and 8-

10 nearby similar areas of carbon support for each condition were imaged using a Tecnai 10 transmission microscope. Numbers of gold beads were counted and gold bead density was expressed as the average of the densities in collected images.

Time lapse video microscopy of *P. falciparum* egress

Video microscopy of *P. falciparum* schizont egress was performed as described previously (Collins et al., 2013b). Synchronised schizonts were Percoll-enriched and further cultured in the presence of the *P. falciparum* PKG inhibitor compound 1 (C1, 2 μM) in RPMI Albumax medium for 4 h. Microscopy chambers (internal volume ~80 μl) for observing live schizonts were constructed by adhering 22 × 64 mm borosilicate glass coverslips to microscope slides with strips of double-sided tape, leaving ~4 mm gaps at each end. The schizonts were pelleted, washed once in warm (37°C) fresh RPMI Albumax without C1 then immediately resuspended into fresh warm medium and introduced by capillary action into the pre-warmed chamber. The chamber was immediately transferred to a temperature-controlled microscope stage at 37°C on an Axio Imager M1 microscope (Zeiss) equipped with an EC Plan-Neofluar 100×/1.3 oil immersion differential interference contrast (DIC) objective and an AxioCam MRM camera. Images were routinely collected at 5 s intervals; beginning 4 min 20 sec after washing off C1, over a total of 30 min. For each experiment, videos of the chim_Δ+can and chim_Δ+mut parasite populations were made alternately over the course of an afternoon in order to ensure that differences in rate of egress were not a result of variation in maturity of the parasite populations. The images were then annotated using Axiovision 3.1 software and exported as AVI movie files. Individual egress events were then annotated by detailed visual analysis of the movies using Microsoft PowerPoint and the delay to the time of egress was recorded for each schizont for subsequent statistical analysis. Mean time to egress was thus determined for each schizont population and plotted as bar graphs for the two different mutants.

In order to further reduce difference of conditions during video microscopy and for better comparison of the chim_Δ+can and chim_Δ+mut parasites, purified schizonts from both populations

were mixed together in the same microscopy chamber prior to imaging. To discriminate between the two transgenic parasite clones, the chim_Δ+mut population was labelled with the DNA labelling dye Hoechst (1 µg/mL) for 5 min prior to washing off the C1. An initial fluorescence image was collected (exposure of 10 ms) and this overlayed with a DIC image. Time lapse video microscopy and analysis of annotated videos were then performed as described earlier. The reciprocal experiment of labelling the chim_Δ+can parasites was then performed to rule out any effects of dye incorporation on time to egress.

Conditional truncation of *P. falciparum* MSP1 using the loxPint/DiCre system

Generation of constructs and transfection of P. falciparum for conditional truncation of the msp1 gene. To obtain conditional truncation of the *msp1* gene we used an approach which uses silent *loxP* sites within a heterologous *P. falciparum* intron (called loxPint). The system is described in detail in a different manuscript (Matthew Jones, Sujaan Das, Michael Blackman and Moritz Treeck, in preparation). Briefly, the loxPint module was introduced by homologous recombination into the *P. falciparum* 3D7 genomic *msp1* gene in two different locations, allowing the creation of two slightly different truncations of MSP1-D (3D7MSP1flox42C1 and 3D7MSP1flox42C2; see Figure S6). For this, two ~400 bp sequences corresponding to the loxPint fragment flanked by targeting sequence at the 5´ and 3´ends (-AGAAACCC**AG**-loxPint-**AT**CCCCACAT- and -CCTCAACC**AG**-loxPint-**AT**GTAACTCC-; letters in bold indicate a naturally occurring AGAT motif which effectively serves as the intron-exon boundary) were synthesised (Geneart®) and introduced into construct pHH1-3D7wt using restriction sites HpaI and BstEII to generate plasmid constructs pMSP1_loxPint-1 and pMSP1_loxPint-2. The structure of each construct was confirmed by nucleotide sequencing on both strands. The constructs were independently transfected into the DiCre-expressing *P. falciparum* 1G5DiCre clone (Collins et al., 2013a) and parasites selected in which the constructs had integrated into the *msp1* genomic locus by single cross-over homologous recombination. The parasite lines were cloned by limiting

dilution to obtain two distinct parasite clones corresponding to each of the different placements of the loxPint, called 3D7MSP1flox42C1 clone E3 and 3D7MSP1flox42C2 clone B51.

To obtain rapamycin-induced excision of the 3′ end of the modified *msp1* gene, deleting coding sequence for the $MSP1_{42}$ fragment and GPI anchoring sequence (Figure 6 and Figure S6), cultures containing synchronised, newly invaded ring forms of clones 3D7MSP1flox42C1 clone E3 and 3D7MSP1flox42C2 clone B51 were divided into two and treated with rapamycin (final concentration 100 nM, diluted 1:100 from a 10 μM stock in DMSO) or mock treated (1:100 dilution of DMSO) for 4 h, then washed to remove the rapamycin and returned to culture as described previously (Collins et al., 2013a). Schizonts purified from the cultures at the end of the same replicative cycle were used in PCR, IFA, or Western blot analysis or for time-lapse video microscopy.

IFA of free merozoites. Purified 3D7MSP1flox42C1 clone E3 or 3D7MSP1flox42C2 clone B51 schizonts from rapamycin-treated or mock-treated control cultures were incubated in medium containing 2 μM C1 for 4 h, then washed and resuspended in warm C1-free medium to allow egress (or abortive egress in the case of the rapamycin-treated mutants). The resulting preparations, which contained free merozoites, were used to prepare thin films for IFA as described above.

Growth assays to determine the effects of MSP1 truncation on parasite replication. Cultures (2% haematocrit) containing synchronous ring stages of clones 3D7MSP1flox42C1 clone E3 and 3D7MSP1flox42C2 clone B51 at 1% parasitaemia were divided into two flasks and either rapamycin treated (100 nM in DMSO) or mock treated with DMSO for 4 h. The parasites were then washed and returned to culture for a total of 4-6 erythrocytic cycles. Samples were collected at the end of every replicative cycle for IFA and parasitemia counts. At the end of the second replicative cycle (day 4) and the fourth cycle in the case of the longer experiments, each cultures was diluted 1:10 by diluting into a 2% haematocrit suspension of fresh erythrocytes in fresh medium. Parasitaemia was determined by microscopic counting of Giemsa-stained thin films and plotted after adjustment to

take dilution factors into account. The parasites in at least 1000 erythrocytes counted at each time point in three independent counts, and the mean parasitaemia values was plotted with SD as error bars. Exponential growth curves were fitted to the data and replication rates per erythrocytic cycle determined from the slopes. To assess relative fitness of the truncated mutants, IFA was performed probing with mAb X509 and mAb 89.1, which gave an estimate of the proportion of non-excised (mAb X509 reactive) parasites as a proportion of all MSP1-positive (mAb 89.1 reactive) parasites in the rapamycin-treated population over several growth cycles.

Supplemental References

Blackman, M.J., Chappel, J.A., Shai, S., and Holder, A.A. (1993). A conserved parasite serine protease processes the Plasmodium falciparum merozoite surface protein-1. Mol Biochem Parasitol *62*, 103-114.

Blackman, M.J., Corrie, J.E., Croney, J.C., Kelly, G., Eccleston, J.F., and Jameson, D.M. (2002). Structural and biochemical characterization of a fluorogenic rhodamine-labeled malarial protease substrate. Biochemistry *41*, 12244-12252.

Blackman, M.J., Fujioka, H., Stafford, W.H., Sajid, M., Clough, B., Fleck, S.L., Aikawa, M., Grainger, M., and Hackett, F. (1998). A subtilisin-like protein in secretory organelles of Plasmodium falciparum merozoites. J Biol Chem *273*, 23398-23409.

Blackman, M.J., Whittle, H., and Holder, A.A. (1991). Processing of the Plasmodium falciparum major merozoite surface protein-1: identification of a 33-kilodalton secondary processing product which is shed prior to erythrocyte invasion. Mol Biochem Parasitol *49*, 35-44.

Child, M.A., Epp, C., Bujard, H., and Blackman, M.J. (2010). Regulated maturation of malaria merozoite surface protein-1 is essential for parasite growth. Mol Microbiol *78*, 187-202.

Collins, C.R., Das, S., Wong, E.H., Andenmatten, N., Stallmach, R., Hackett, F., Herman, J.P., Muller, S., Meissner, M., and Blackman, M.J. (2013a). Robust inducible Cre recombinase activity in the human malaria parasite Plasmodium falciparum enables efficient gene deletion within a single asexual erythrocytic growth cycle. Mol Microbiol *88*, 687-701.

Collins, C.R., Hackett, F., Strath, M., Penzo, M., Withers-Martinez, C., Baker, D.A., and Blackman, M.J. (2013b). Malaria parasite cGMP-dependent protein kinase regulates blood stage merozoite secretory organelle discharge and egress. PLoS Pathog *9*, e1003344.

Collins, C.R., Withers-Martinez, C., Hackett, F., and Blackman, M.J. (2009). An inhibitory antibody blocks interactions between components of the malarial invasion machinery. PLoS Pathog *5*, e1000273.

Crosnier, C., Wanaguru, M., McDade, B., Osier, F.H., Marsh, K., Rayner, J.C., and Wright, G.J. (2013). A library of functional recombinant cell-surface and secreted P. falciparum merozoite proteins. Mol Cell Proteomics *12*, 3976-3986.

Epp, C., Raskolnikov, D., and Deitsch, K.W. (2008). A regulatable transgene expression system for cultured Plasmodium falciparum parasites. Malar J *7*, 86.

Harris, P.K., Yeoh, S., Dluzewski, A.R., O'Donnell, R.A., Withers-Martinez, C., Hackett, F., Bannister, L.H., Mitchell, G.H., and Blackman, M.J. (2005). Molecular identification of a malaria merozoite surface sheddase. PLoS Pathog *1*, 241-251.

Holder, A.A., and Freeman, R.R. (1982). Biosynthesis and processing of a Plasmodium falciparum schizont antigen recognized by immune serum and a monoclonal antibody. J Exp Med *156*, 1528-1538.

Holder, A.A., Lockyer, M.J., Odink, K.G., Sandhu, J.S., Riveros-Moreno, V., Nicholls, S.C., Hillman, Y., Davey, L.S., Tizard, M.L., Schwarz, R.T., *et al.* (1985). Primary structure of the precursor to the three major surface antigens of Plasmodium falciparum merozoites. Nature *317*, 270-273.

Kauth, C.W., Epp, C., Bujard, H., and Lutz, R. (2003). The merozoite surface protein 1 complex of human malaria parasite Plasmodium falciparum: interactions and arrangements of subunits. J Biol Chem *278*, 22257-22264.

Kilili, G.K., and LaCount, D.J. (2011). An erythrocyte cytoskeleton-binding motif in exported Plasmodium falciparum proteins. Eukaryot Cell *10*, 1439-1447.

Koussis, K., Withers-Martinez, C., Yeoh, S., Child, M., Hackett, F., Knuepfer, E., Juliano, L., Woehlbier, U., Bujard, H., and Blackman, M.J. (2009). A multifunctional serine protease primes the malaria parasite for red blood cell invasion. Embo J *28*, 725-735.

O'Donnell, R.A., Hackett, F., Howell, S.A., Treeck, M., Struck, N., Krnajski, Z., Withers-Martinez, C., Gilberger, T.W., and Blackman, M.J. (2006). Intramembrane proteolysis mediates shedding of a key adhesin during erythrocyte invasion by the malaria parasite. J Cell Biol *174*, 1023-1033.

Pan, W., Ravot, E., Tolle, R., Frank, R., Mosbach, R., Turbachova, I., and Bujard, H. (1999). Vaccine candidate MSP-1 from Plasmodium falciparum: a redesigned 4917 bp polynucleotide enables synthesis and isolation of full-length protein from Escherichia coli and mammalian cells. Nucleic acids research *27*, 1094-1103.

Rudolph, R., and Lilie, H. (1996). In vitro folding of inclusion body proteins. FASEB journal : official publication of the Federation of American Societies for Experimental Biology *10*, 49-56.

Sreerama, N., and Woody, R.W. (2004). Computation and analysis of protein circular dichroism spectra. Methods in enzymology *383*, 318-351.

Withers-Martinez, C., Saldanha, J.W., Ely, B., Hackett, F., O'Connor, T., and Blackman, M.J. (2002). Expression of recombinant Plasmodium falciparum subtilisin-like protease-1 in insect cells: Characterization, comparison with the parasite protease, and homology modelling. J Biol Chem *277*, 29698-29709.
